# Splicing and the Evolution of Proteins in Mammals

**DOI:** 10.1371/journal.pbio.0050014

**Published:** 2007-02-06

**Authors:** Joanna L Parmley, Araxi O Urrutia, Lukasz Potrzebowski, Henrik Kaessmann, Laurence D Hurst

**Affiliations:** 1 Department of Biology and Biochemistry, University of Bath, Bath, United Kingdom; 2 Center for Integrative Genomics, Genopode, University of Lausanne, Lausanne, Switzerland; University of Dublin, Ireland

## Abstract

It is often supposed that a protein's rate of evolution and its amino acid content are determined by the function and anatomy of the protein. Here we examine an alternative possibility, namely that the requirement to specify in the unprocessed RNA, in the vicinity of intron–exon boundaries, information necessary for removal of introns (e.g., exonic splice enhancers) affects both amino acid usage and rates of protein evolution. We find that the majority of amino acids show skewed usage near intron–exon boundaries, and that differences in the trends for the 2-fold and 4-fold blocks of both arginine and leucine show this to be owing to effects mediated at the nucleotide level. More specifically, there is a robust relationship between the extent to which an amino acid is preferred/avoided near boundaries and its enrichment/paucity in splice enhancers. As might then be expected, the rate of evolution is lowest near intron–exon boundaries, at least in part owing to splice enhancers, such that domains flanking intron–exon junctions evolve on average at under half the rate of exon centres from the same gene. In contrast, the rate of evolution of intronless retrogenes is highest near the domains where intron–exon junctions previously resided. The proportion of sequence near intron–exon boundaries is one of the stronger predictors of a protein's rate of evolution in mammals yet described. We conclude that after intron insertion selection favours modification of amino acid content near intron–exon junctions, so as to enable efficient intron removal, these changes then being subject to strong purifying selection even if nonoptimal for protein function. Thus there exists a strong force operating on protein evolution in mammals that is not explained directly in terms of the biology of the protein.

## Abstract 2

Intron-exon boundaries, once fixed in proteins, are found to be subject to purifying selection, even if they are not optimal for protein function.

## Author Summary

Most of the DNA in our genes is actually not involved in the specification of proteins. Rather, the bits with the protein-coding information (exons) are separated from each other by noncoding bits, introns. Before a gene can be translated into protein these introns are removed and the exons are spliced back together to be translated into protein. While information about which DNA to remove is largely in the introns themselves, parts of the exons near the intron–exon boundary can, for example, function as splice enhancer elements. In principle, then, these parts of exons have two functions: to specify the amino acids of the resulting protein and to enable the correct removal of introns. What impact might this have on a gene's evolution? We show that near intron–exon boundaries, amino acid usage is biased towards nucleotides involved in splice control. Moreover, these parts of genes evolve especially slowly. Indeed, we estimate that a gene with many exons would evolve at under half the rate of the same gene with no introns, simply owing to the need to specify where to remove introns. Likewise, genes that have lost their introns evolve especially fast near the former intron's location. Thus, human proteins may not be as optimised as they could be, as their sequence is serving two conflicting roles.

## Introduction

Why do some parts of proteins evolve more slowly than others? Why, in turn, do some proteins evolve more slowly than others? Intragenic conserved regions are typically considered to reflect domains of functional importance to the protein [1]. Similarly, proteins with a high density of important functional sites should evolve slowly. There are, however, potentially multiple other correlates to rates of protein evolution [1]. The expression parameters of a gene (rate of expression, protein abundance, and number of tissues in which a gene is expressed) are consistently reported to be important predictors [2–5]. This may in part reflect selection to resist mistranslation [6]. Other possible covariates include essentiality and the number of protein interactions, but the issues here are more contentious, not least because of covariance with expression parameters [7–17]. Here we test the hypothesis that selection acting to ensure that introns are correctly removed skews amino acid content in predictable ways and imposes constraints on rates of protein evolution.

In mammalian genes, which are rich in introns [18], correct removal of introns often requires the presence, in the flanking exons, of splice-enhancer domains, these being short (six nucleotide) blocks required for binding of serine/arginine-rich proteins [19]. The need for splice enhancers can impact the use of synonymous codons in the domains flanking intron–exon junctions, such that when a synonymous codon is used commonly in splice enhancers it is preferred over its less commonly used synonym [20,21]. Moreover, selection to preserve splice enhancers affects both the synonymous single nucleotide polymorphism profile [22,23] and the rate of evolution at synonymous sites of splice-enhancer-associated domains [24].

Might the same forces also act to cause skews in amino acid usage in the vicinity of intron–exon junctions? In a preliminary analysis, we showed that there is a tendency for enrichment near boundaries of an amino acid whose codons are common in splice enhancers: lysine is coded by AAA and AAG, both of which are common in splice enhancers, and at both 5′ and 3′ ends of exons, lysine's proportional usage increases [24]. Is it more generally the case that an amino acid's usage increases near intron–exon junctions if it commonly features in splice enhancers? Conversely, are some amino acids avoided near such boundaries if they are rare in splice-enhancer domains? To address these issues, we derive patterns of amino acid preference in the vicinity of intron–exon boundaries and compare these patterns with a metric of enrichment of amino acids in splice enhancers relative to rates of usage in the genome. In turn, we ask whether selective constraints are stronger near intron–exon boundaries, and whether such constraints explain much of the variation between proteins in their rate of evolution.

## Results

### Amino Acid Preferences near Intron–Exon Junctions Are Common

For 178,382 human exons we considered the trends in amino acid composition as one approaches the intron–exon boundary, as assayed by the rank correlation, rho, between distance from the boundary and proportional usage of the amino acid. Considering the 2-fold and 4-fold blocks of the 6-fold degenerate amino acids as different groupings, we found that of 46 independent comparisons (23 amino acid groups 5′ and 3′ prime), 34 showed significant trends for enrichment or avoidance near intron–exon boundaries (Table 1). After Bonferroni correction 26 remained significant (with 46 comparisons *p* < 0.001 indicates significance). For all plots for individual amino acids see Figure S1. We repeated the analysis for 115,466 exons from 14,005 mouse genes and found that patterns of preference are strikingly similar between the two species (Table 1). In mice, 34 amino acids again showed significant trends, and the correlation of rho values for 46 comparisons in mice versus human was extremely high (Pearson product moment correlation, *r* = 0.96, *p* < 0.0001).

**Table 1:**
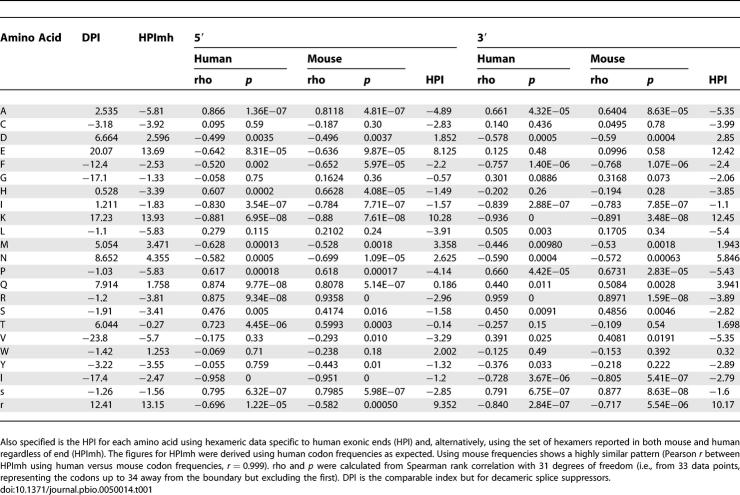
Trends in Avoidance of (rho > 0) or Preference for (rho < 0) Amino Acids as a Function of Distance from the Intron–Exon Junction

Do these effects necessarily relate to the nucleotide content of the codons, as the splice-regulation model requires? One might conjecture instead that these effects reflect some coincidence of exon boundaries with protein substructures having unusual amino acid contents. Several facts strongly support the hypothesis that the trends seen are at least in part driven by effects at the nucleotide level. Notably, while the 2-fold block of arginine (amino acid r in Table 1) was strongly preferred near boundaries at both 3′ and 5′ ends, the 4-fold redundant block (amino acid R) showed the reverse pattern. A comparable difference was seen for the 2-fold (amino acid l) and 4-fold (amino acid L) blocks for leucine. The same pattern was seen in mouse genes. A preference for certain amino acids, regardless of the nucleotide content of their codons, would not have predicted this.

### Amino Acid Preferences near Intron–Exon Junctions Are Predicted by Involvement in Splice-Enhancer Domains

If splice-enhancer domains impact amino acid usage near intron–exon boundaries, we expect that those amino acids preferred in splice enhancers should be preferred near junctions (i.e., rho < 0). To test this we developed a metric of involvement of codons in splice-enhancer hexamers, which we term the hexamer preference index (HPI). Using hexamers found both in mouse and human to define the HPI (and ignoring 3′ and 5′ differences), we found a striking predictability of patterns of preference near boundaries (Spearman rank correlation between HPI and rho for preference/avoidance near boundaries, rho = −0.54, *p* < 0.0001, *n* = 46). As an alternative to rho, we can employ the slope of the best-fit regression line between proportional usage of an amino acid and distance from intron–exon junctions. A negative slope, like a negative rho, indicates preferential usage near junctions. Using this slope on the best-fit regression line revealed, as expected, the same trend (Spearman rank correlation, slope versus HPI = −0.57, *p* < 0.0001; Figure S2). The trend for preference of high HPI amino acids near boundaries was also seen in mice (e.g., using mouse–human overlap set of hexamers, correlation of rho with HPI = −0.49, *p* = 0.0005; correlation of slope with HPI = −0.52, *p* = 0.0002).

**Figure 1:**
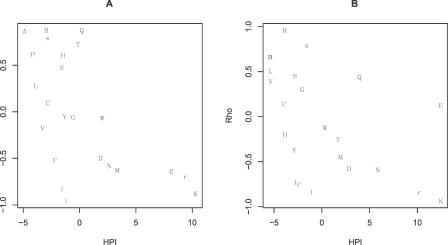
The Relationship between Tendency for an Amino Acid to Be Preferred near Exon–Intron Junctions (rho < 0) or Avoided (rho > 0) and the HPI (A) 5′ exonic ends and (B) 3′ ends.

These results are not greatly affected by considering 5′ and 3′ ends separately (Spearman rank correlation between rho 5′ and HPI 5′ using human 5′-specific hexamers = −0.59, *p* = 0.003, *n* = 23, Figure 1A; between rho 3′ and HPI 3′ using human 3′-specific hexamers = −0.57, *p* = 0.004, *n* = 23, Figure 1B). This is reflected in the fact that trends in usage (rho) and patterns of HPI are similar 5′ and 3′ (Pearson correlation, *r*, between rho 5′ and rho 3′ for the 23 amino acid classes = 0.80, *p* < 0.0001; Pearson correlation between HPI 5′ and HPI 3′ for the 23 amino acid classes = 0.95, *p* < 0.0001).

**Figure 2:**
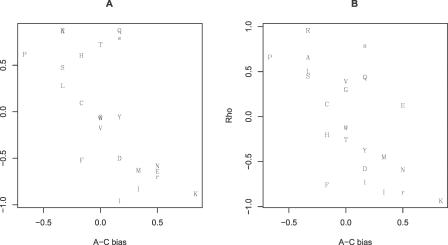
AC Bias in the Codon Set of a Given Amino Acid and Its Relationship to Amino Acid Usage near Exon–Intron Junctions (A) 5′ exonic ends and (B) 3′ ends.

One might suppose that our measure of HPI might be biased by incomplete knowledge of enhancers. We can control for this, in part, by recognizing that splice enhancers tend to be adenine rich and cytosine poor. Consider then the composite measure AC bias = frequency of adenine in synonymous codon set – frequency of cytosine. For example, in the 4-fold degenerate set for alanine (GCN), of the 12 bases in four possible synonymous codons, adenine and thymine both featured 1/12 of the time, and guanine and cytosine both featured 5/12 of the time. So AC bias for alanine is 1/12 − 5/12 = −1/3. This AC bias was a robust predictor of preference/avoidance near boundaries (Spearman rank correlation, AC bias versus rho = −0.67, *p* < 0.0001) (Figure 2). Avoidance of cytosine in the synonymous codons appeared to be a somewhat stronger predictor of patterns of avoidance or preference of amino acids than was preference for adenine (Spearman rank correlation, cytosine content of codons versus rho = 0.67, *p* < 0.0001; adenine content of codons versus rho = −0.37, *p* = 0.01). Neither thymine nor guanine content showed any trends (*p* >> 0.05). These results suggest that the general profile of enhancers and the specifics employed to define HPI are about equally good predictors of patterns of preference/avoidance.

### Rates of Evolution Are Reduced near Intron–Exon Boundaries and in Genes Rich in Introns

The above results suggest that selection acts to prefer nucleotides that permit efficient intron removal. Does this in turn affect rates of protein evolution? Were there such an effect, we should expect that smaller exons should evolve more slowly, as a higher proportion of sequence is near (e.g., within 70 bp) boundaries. Indeed, from a set of 36,683 mouse–human aligned exons, we found that small exons do tend to have low rates of evolution (Spearman rank correlation between the number of nonsynonymous substitutions per nonsynonymous site [*K*_A_] and exon length, rho = 0.15, *p* < 0.0001). This might, however, be owing to a trend for genes with small exons to be disproportionately in functional classes of protein that have intrinsically low rates of evolution. To control for this we considered, for all genes with more than two internal exons, the Spearman rank correlation between exon *K*_A_ and the size of the exon. As each correlation coefficient is derived from a given gene, between-gene variation in *K*_A_ (and indeed the number of synonymous substitutions per synonymous site [*K*_S_]) is controlled for in any such analysis. If splice control impacts rates of exon evolution we expect that on the average this correlation should be positive, while the null hypothesis, that small exons have low rates of evolution because they derive from classes of genes with intrinsically low *K*_A_, predicts a mean rho of zero. The distribution of rho was very strongly skewed to positive values (median rho = +0.14, Wilcoxon rank test, *p* < 0.0001, *n* = 3,629). Restricting analysis to genes with ten or more exons only strengthened this conclusion (median rho = +0.16, *p* < 0.0001, *n* = 1,286).

**Figure 3:**
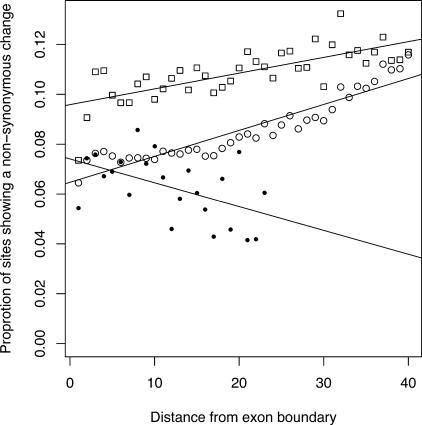
Rate of Nonsynonymous Evolution as a Function of the Distance from an Intron–Exon Boundary The proportion of informative sites in intron-containing genes showing a nonsynonymous change in the human–mouse comparison (all exons, circles; exons > 80 codons, squares), and the proportion of informative sites in retrogene sequences showing retrogene-specific changes as a function of distance from what was originally the exon–intron boundary (black spots) and as a function of the distance from the real exon boundary.

Is there also a trend for lower rates of evolution near boundaries? Using all exons, asking about the proportion of all sites a given distance from a boundary (5′ or 3′) in which we see a nonsynonymous change, we observed the predicted low rate of amino acid evolution near boundaries (Spearman rank correlation, proportion of aligned sites showing nonsynonymous change versus distance from boundary, rho = 0.955, *p* < 0.0001) (Figure 3, circles). Might this result simply be an artefact of the possibility that small exons might both come disproportionately from a class of slow-evolving genes and contribute more data to the estimate of divergence near the exon–intron junctions than they do to the more distant sites? To control for this, we again considered divergence rates within 40 codons of boundaries (5′ and 3′) but considered only the 1,836 exons that are at least 80 codons long. This way all exons contribute approximately the same amount of data at all distances from the junction. We found that the lower rate of evolution near the boundary remained highly robust (rho = 0.7685, *p* < 0.0001) (Figure 3, squares).

Note, however, that absolute rates of evolution, at any given distance from the boundary, were higher in this long exon set. This is consistent either with reduced density of splice-control elements near boundaries in long exons or with a splice-unrelated force acting more profoundly on long exons. There is good evidence for the former. When we examined the density of putative exonic splice enhancers (ESEs) in the exon span within 100 bp of a boundary at either end (or all of the exon in the case of exons shorter than 200 bp), we found a robust negative correlation between enhancer density and exon size (rho = −0.18, *p* < 0.0001). Comparably, when we considered exons longer than 200 bp to be long exons and those shorter than this to be short exons, we found that ESEs occupy a median of 31% of the short exons, but only 21% of the 200 bp near the boundaries (100 bp 5′ and 100 bp 3′) of the long exons. This is consistent with the idea that there is less space in short exons to pack in the information necessary to enable proper splicing.

**Figure 4:**
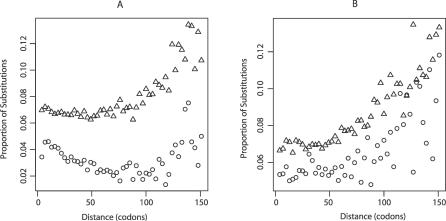
Rate of Nonsynonymous Evolution as a Function of the Distance from an Intron–Exon Boundary for ESS and Non-ESS Sequence The rate of evolution of sequences defined as part of ESSs (circles) and those not in enhancers (triangles) is shown as a function of the distance from exon boundaries in the mouse–human analysis at (A) the 5′ end of exons and (B) the 3′ end of exons. To define putative enhancer sequence the mouse and human sequence was matched to the set of species-specific, exon-end-specific set of hexamers. Any part of the alignment not found to be enhancer in either species was considered nonenhancer. Any part of the alignment found to be enhancer in both was considered to be enhancer sequence. As can be seen, exonic enhancer sequence evolves more slowly than nonenhancer. Given that functional splice enhancers are rare more than 100 bp from a boundary, it is expected that the further into the exon, the less the difference between enhancer and nonenhancer.

As expected, *K*_A_ was lower in ESEs than in nonenhancers (Figure 4) (see also [24]). This was also true if we restricted analysis to exons longer than 200 bp (paired test, *p* < 0.0001) (Figure S3). These results tally with the finding that genes with long introns tend to have low rates of evolution [12], as exons flanked by long introns tend to be richest in ESEs [25].

**Figure 5:**
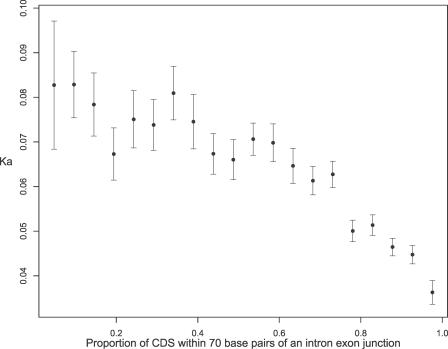
The Relationship between *K*_A_ in the Mouse–Human Comparison and the Proportion of Sequence within 70 bp of an Exon–Intron Junction Error bars show standard error of the mean. CDS, coding sequence.

As expected from the above results, genes with a high proportion of sequence within, for example, 70 bp of an intron–exon junction showed lower *K*_A_ (Table 2; Figure 5). Using alternative bounds (50 or 100 bp) did not qualitatively affect conclusions (Table 2). The difference between a gene with all sequence within 70 bp of exon boundaries and one with very little (<10%) was striking (mean *K*_A_ = 0.032 for those with small exons and 0.083 for those with less than 10% of sequence near junctions). Were all things equal, this result suggests that the rate of evolution of an intron-rich gene is on average approximately under 40% that of an intron-poor gene.

**Table 2:**
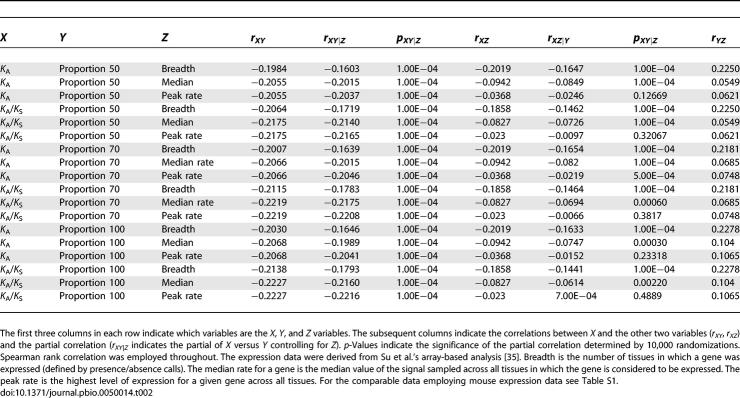
Correlations and Partial Correlations between Rate of Protein Evolution (*K*_A_ or *K*_A_/*K*_S_), Proportion of Sequence within 50, 70, or 100 bp of an Intron–Exon Junction, and Measures of Expression of the Relevant Gene in Humans

It is, however, unlikely that all things are equal. To allow for this, we performed a paired test. For each gene we concatenated all sequences in the alignment flanking (within 72 nucleotides) intron–exon boundaries, both 5′ and 3′, and concatenated all of the middle sections of exons (defined as anything beyond 72 nucleotides). As before we considered only internal exons. We then calculated *K*_A_ for the concatenated flanks and the concatenated middle sections and considered the gene-specific ratio of the two. We then considered the mean of the gene-specific ratio for all genes.

By necessity we had to eliminate all genes with no exon larger than 144 bp, leaving 3,058 genes. Moreover, as accurate estimation of *K*_A_ probably requires a minimum of 100 codons, we restricted analysis to those genes with at least 300 bp in the concatenated flanks and in the concatenated middle of exons. We found that the mean ratio of the rate of evolution (*K*_A_) of the middle part of exons to the flanks within the same gene was 1.93 (Wilcoxon signed rank test, *p* < 0.0001, *n* = 666). Requiring at least 600 bp in both flanks and middle sections, the middle was estimated to evolve 2.3 times faster than the flanks. When we considered the exon flanks to be 102 bp, the mean ratio of middle to flank was 2.5 when requiring a minimum of 300 bp in each class (*n* = 368). Requiring a minimum of 600 bp, the middle parts of exons evolved on average 2.7 times faster than the exon flanks from the same genes (*n* = 167). Overall, then, it seems safe to conclude that exon centres evolve at about 2.3 times the rate of exon flanks from the same gene, the precise estimate depending on parameter choices.

These results demonstrate that exon flanks evolve more slowly than exon centres, regardless of the functional class of the protein. The mean *K*_A_ of flanking domains was around 0.04 in the above samples. A gene with short exons should then have approximately a *K*_A_ of 0.04, controlling for between-gene heterogeneity. By contrast one with 90% of sequence not near boundaries should have a *K*_A_ of on average around 0.086, assuming exon centres of such long exons evolve 2.3 times faster than flanks (0.04 × 2.3 × 0.9 + 0.04 × 0.1 = 0.086). Controlling then for functional class, we estimated that a gene with all sequence near intron–exon boundaries should evolve at about 46% (0.04/0.086) the rate of one with proportionally little sequence near boundaries.

This estimate can be downwardly adjusted if we consider that some of the genes with long exons have more than 90% of sequence near boundaries: at the limit intronless genes should evolve with *K*_A_ ≅ 0.092, i.e., at 2.3 times the rate of small exon genes. Likewise, if our estimate of the ratio of rates of evolution is higher, then the discrepancy between intron-poor and intron-rich genes will be greater. Using the 2.7 ratio, for example, intron-rich genes evolve at 37% of the rate of intronless genes, controlling for protein function. Equally, the estimate can be upwardly adjusted if we presume a more modest ratio of rates of evolution of internal parts of exons to flanks. Overall, it seems fair to suppose that constraints imposed in the proximity of intron–exon boundaries can reduce the rate of evolution of a gene by a half or more, if the gene is full of small exons rather than lacking introns. That this is similar to the prior estimate, not controlling for between-gene heterogeneity, suggests that selection on exon flanks is a major determinant of rates of evolution.

### Comparing Constraints Owing to Splicing with Other Correlates of Rates of Evolution

How does the effect of selection in the vicinity of intron–exon junctions compare with and covary with other strong predictors of rates of protein evolution? In principle any relationship between rate of protein evolution and proportion of sequence near a boundary might in part be because genes with many introns tend to be housekeeping genes [26], and housekeeping genes (those expressed in many tissues) tend to have low rates of evolution [4,27,28]. The two parameters (expression breadth and proportion of sequence near boundaries) both appear, however, to be good predictors when controlling one for the other (Table 2). Use of alternative metrics of gene expression (mean rate and peak rate) (see Table 2) make no qualitative difference to the conclusion that, before and after control for covariates, the proportion of sequence near intron–exon junctions is at least as strong a predictor of rates of evolution as expression parameters, if not stronger.

After expression parameters, the dispensability of a protein may, in mammals, also be a good predictor [12]. From a sample of 1,198 mouse genes for which knockout experiments have resolved whether they are essential or not, and for which we have orthologues, we can ask whether essential and nonessential genes (a) differ in their proportion of sequence near intron–exon junctions and (b) differ in their rate of evolution. Confirming the prior report [12], we found that essential proteins evolve at about two-thirds the rate of nonessential ones (mean *K*_A_ for nonessential proteins, 0.07; for essential proteins, 0.049; *p* < 0.0001, Mann-Whitney U test). However, the two classes are no different as regards the proportion of sequence near intron–exon boundaries (mean proportion of sequence near boundaries for nonessential proteins, 0.618; for essential proteins, 0.607; *p* = 0.67, Mann-Whitney U test). There is, therefore, no reason to suppose that the lower rate of evolution of genes with much sequence near intron–exon boundaries is owing to their being more likely to be essential. Equally, there is no reason to suppose that the lower rate of evolution of essential genes is owing to their having more sequence near intron–exon boundaries. Note too that the difference in evolutionary rate between essentials and nonessentials is more modest than that between genes with high and low proportion of sequence near intron–exon junctions. The majority of our sample is of unknown dispensability. These genes have a mean *K*_A_ of 0.059, more or less as expected, given the means for the essential and nonessential genes and assuming that 30% of mouse genes are essential [12].

### Retrogenes and Loss of Selective Constraint near Intron–Exon Junctions

Let us now consider two models for what might happen after a new intron has been inserted. In the first, a new intron might be favoured only if enough splice-enhancer domains in adequate proximity are already present to enable efficient removal of the intron (model 1). An alternative model (model 2) might suppose that immediately after introduction of a new intron, proper excision, owing to a dearth of local splice enhancers, is not always possible. If, then, some transcripts preserve the original mRNA by proper excision, but others fail to so do, the new intron would effectively reduce the rate of protein production for a given transcription rate. Such a mutation might be weakly deleterious such that fixation through drift is still possible. Selection may then favour shifts in amino acid usage to enable more efficient splicing. The second model is especially interesting as it suggests that intra-protein amino acid usage is not dictated simply by protein requirements alone.

Both models predict that should enhancer domains be employed, they may then be under selection to preserve functionality. Both also predict that amino acids that feature commonly in the hexameric sequences describing splice enhancers should be more common near intron–exon junctions, as observed. How they differ is in the prediction of subsequent evolution following gain/loss of introns. Model 1 supposes that if an intron inserts but is not successfully removed owing to a dearth of splice-enhancer domains in the near vicinity, the insertion may simply be too deleterious to be tolerated and is hence lost from the population. By contrast, model 2 considers the possibility that compensatory nonsynonymous changes can further occur that permit more efficient intron removal.

To discriminate these two classes, one needs a sufficiently sized dataset of intron losses or gains in humans. Unfortunately, intron gain appears to be vanishingly rare in humans and mammals more generally. However, functional retroposed genes do provide a means to ask about the consequences of intron loss. Is it then the case that, after retroposition, the residues that, in the original parental copy of the gene, flanked intron–exon junctions are more prone to change?

We examined a set of 49 old functional retroposed genes for which, in all cases, there existed mouse and human parent and retroposed sequences. For all sites in the alignment that specified an amino acid in all four lineages, we considered the proportion of retrogene-specific changes (see Materials and Methods). We then considered how this varied as a function of the distance from what was, in the parental gene, the intron–exon boundary. Merging figures for 3′ and 5′ ends, we found that the rate of evolution in retrogenes is higher close to what was the boundary (Spearman rank correlation, proportion of sites subject to change in retrogenes versus distance from ancient boundary, rho = −0.48, *p* = 0.019) (Figure 3). Moreover, retrogenes that are derived from genes in which a high proportion of the sequence was near exon boundaries (genes with predominantly small exons) tended to have higher overall rates of evolution (proportion of parent sequence 70 bp from boundary versus number of retrogene-specific changes per base pair, rho = +0.38, *p* = 0.008, *n* = 49).

The difference in behaviour between genes that have lost their introns and intron-containing genes (Figure 3) suggests that constraints that exist near intron–exon boundaries have been released in the retrogenes, and, hence, that these sites are now free to change. This evidence, therefore, lends some support to the converse possibility, namely that, after intron insertion, exonic domains flanking the new boundary changed, probably to permit better splicing. The result does not specifically show that all the change involved the evolution of new splice enhancers; however, with the data showing that the HPI predicts trends in amino acid usage near junctions and low nonsynonymous rates in ESEs (Figure 4) [24], this is likely to explain much of the effect.

## Discussion

We have found that, in both mouse and human, most amino acids show skewed usage in the vicinity of intron–exon junctions. These patterns appear owing to preference at the nucleotide level, as evidenced by the different behaviours of the 2-fold and 4-fold blocks of leucine and arginine. To a first approximation, the patterns are well explained by the abundance of the relevant codons, relative to levels in the genome, in splice enhancers. The preferences are also reflected in reduced rates of evolution near intron–exon boundaries and in intron-rich genes more generally. Indeed, the proportion of sequence near intron–exon boundaries is, to the best of our knowledge, one of the strongest predictors to date of rates of protein evolution (for analysis of alternatives see [12]). That in retrogenes the domains that used to be near intron–exon junctions show increased rates of evolution supports the view that intron–exon junctions are domains on which constraint operates. Were it the case that new introns are only tolerated if the full repertoire of splice-control elements is already in place, we would not expect that, on loss of introns, these domains would show unusually high rates of evolution. Although by necessity our sample size of retrogenes is small, we suggest that model 2, evoking evolution to modify amino acid content after intron insertion, is more parsimonious.

Whether the elements being preferred are necessarily and exclusively splice enhancers remains uncertain. First, as can be seen in Figure 4, sequence putatively not in enhancers is more highly constrained near boundaries, at least at the 3′ end. This suggests the possibility of constraint imposed near boundaries independent of splice enhancers and/or inaccuracy in the definition of enhancers. Further, there are a few strong outliers in the distribution of HPI versus preference near boundaries (Table 1). In human sequences, of 46 comparisons, 14 fail to match with the expectation that if HPI is negative, rho should be positive and vice versa, of which nine are significant and six significant after Bonferroni correction: I5′, l5′, Q5′, F3′, I3′, and l3′ (Table 1). Glutamine (CAA and CAG) is unique in being preferred in splice enhancers and avoided both 3′ and 5′ at boundaries. Three amino acids are strongly preferred near boundaries (rho << 0) but disfavoured in splice enhancers (HPI < 0), these being the 2-fold degenerate codons of leucine (TTA and TTG), isoleucine (ATC, ATA, and ATT), and phenylalanine (TTC and TTT). Tyrosine (TAC and TAT) may be a weaker outlier (rho < 0 both 5′ and 3′, HPI < 0). The same outliers are seen in mouse genes (Table 1).

Are these apparent exceptions instructive of some other force driving amino acid choice near boundaries, or might they reflect limitations in our understanding of splice-enhancer hexamers? Were the latter the case we might expect that a surrogate measure of involvement in splice enhancers might reveal these exceptions to simply have poorly described roles in splice enhancers. As noted above adenine and cytosine content of the synonymous codon blocks of each amino acid well predicts HPI (Figure 2). Fitting the best-fit regression of AC bias to rho (using both 5′ and 3′ data), we indeed find from inspection of the standardised residuals (Figure S4) that, both 3′ and 5′, isoleucine and leucine usage now sit within the 95% confidence intervals, as does phenylalanine usage 5′. However, phenylalanine usage 3′ is a little outside the line, as is glutamine 5′ usage.

Another possibility is that the presence of exonic splice suppressors may impact amino acid usage. Wang et al. [29] have identified 131 decamers that function as splice suppressors. We therefore adapted our method to calculate a decamer preference index (DPI) to correspond with these splice suppressors (Table 1). DPI and HPI are not themselves correlated (for mouse–human set for HPI, Spearman rank correlation between HPI and DPI = −0.05, *p* = 0.7). Relating DPI scores to either the slope or the rho values for amino acid preference, we find only a marginal tendency for DPI to explain amino acid preferences (Spearman rank correlation, rho versus DPI, −0.27, *p* = 0.07; slope versus DPI, −0.26, *p* = 0.07). Splice suppressors hence appear to have less impact on amino acid usage than do splice enhancers. Taking a combined measure, the mean of DPI and HPI, marginally improves the fit between amino acid preference and involvement in splice regulation (Spearman rank correlation between mean of DPI and HPI and rho, −0.61, *p* < 0.0001; for HPI alone, −0.54, *p* < 0.0001). AC bias remains a better predictor. Involvement in splice suppressors may, however, explain some of our apparent exceptions. Notably, phenyalanine and the 2-fold block of leucine, while having a negative HPI, have a strongly positive DPI (9.8 and 14.9, respectively). Similarly, glutamine, while having a positive HPI, has a strongly negative DPI (−6.1). The converse roles of these amino acids in splice enhancers and splice suppressors may hence explain their apparently aberrant behaviour. Indeed, on a plot of the mean of DPI and HPI these amino acids no longer appear as outliers (Figure S5). Isoleucine remains an exception, being negative for both HPI and DPI but preferred near boundaries.

The only other model for selection near intron–exon junctions, the so-called cryptic splice-site avoidance model [21,30], does not predict any tendency for cytosine avoidance near boundaries. The relevance of this model is unclear as both AG[A|G] (arginine) and AG[C|T] (serine) appear to have patterns of usage near boundaries at both 5′ and 3′ ends as expected given their HPI scores, whereas the cryptic splice-site avoidance model would predict avoidance at 5′ ends. This model cannot also obviously explain why 3′ usage of phenyalanine might be discordant.

**Figure 6:**
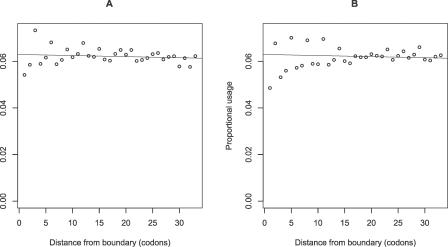
Glycine Usage as a Function of Distance from 5′ and 3′ Exonic Ends (A) 5′ and (B) 3′ exonic ends.

One further striking peculiarity is notable. The profile of usage of glycine (GGN) shows a curious pattern at both 3′ and 5′ ends (also seen in mouse, data not shown) (Figure 6): at every third codon the usage is much higher than at the intervening distances from the boundary. With the sample sizes in question (∼10,000 glycines at these positions), this is not a sample-size artefact. The effect is highly repeatable, being found regardless of the phase of the exon (Figure S6). At both the 3′ and 5′ ends, it is found for all of the four (GGN) codons when analysed separately, although it may be most pronounced for GGA (data not shown). This appears to reflect a pattern at the protein level, at least in part owing to collagens, whereby glycines are very commonly three apart (see Figure S7). Given that introns tend to prefer G|G insertion sites, codons starting GG may well be hot spots for insertion, potentially at all positions. This together with the apparent periodicity in the occurrence of glycine might explain the observations. We leave this to future analysis. Whatever the cause, it points to a limitation of our method, which assumes that trends towards boundaries are monotonic and consistent. For the most part (see Figure S1) these assumptions appear relatively sound, although 5′ usage of proline suggests a U-shaped function.

The hypothesis that the domains under constraint are uniquely splice enhancers might also predict that amino acids not having a role in splice enhancers tend to be gained in retrogenes in boundary proximal domains. Unfortunately, from a sample of 803 gains/losses for retrogenes and 229 in parental genes in regions near intron–exon junctions (<30 codons), we find no amino acid showing statistically significant differences between parental and retrogenes. However, the top three most discordant amino acids (judged by the chi-squared value) all show net gain in the retrogenes and net loss in the parental genes, and, as might be predicted, are all avoided in splice enhancers. These are the 4-fold block of leucine (49 gains to 39 losses in retrogenes; nine losses to 18 gains in parental genes; chi-squared = 4.13), histidine (20 gains to 13 losses in retrogenes; three gains to seven losses in parental genes; chi-squared = 2.89), and the 4-fold block of serine (48 gains to 29 losses in retrogenes; 14 gains to 17 losses in parental genes, chi-squared = 2.66; N.B., for three degrees of freedom *p* < 0.05 occurs at chi-squared > 7). It would be unwise to read too much into this observation, not least because there are several other amino acids with strong avoidance in splice enhancers that show no evidence of switching substitutional profile (notably alanine, cysteine, phenylalanine, and valine). No amino acids show any good evidence for being gainers in the parental gene but losers in the retrogene. Firmer conclusions regarding the patterns of amino acid loss and gain will require larger sample sizes.

Given the outliers being possibly explained by splice-suppressor roles and the strange behaviour of glycine, we do not wish to suggest that the need for splice enhancers determines all amino acid bias, nor all constraint, seen near intron–exon boundaries. Constraints operating near intron–exon boundaries not explained by splice enhancers may nonetheless reflect selection on splice regulation of some form (e.g., exonic splice suppressors). These caveats aside, it is notable that constraints in the vicinity of intron–exon boundaries appear to be one of the stronger, if not the strongest, predictors of rates of protein evolution in mammals. Naturally, for intron-poor genomes the same will not apply.

## Materials and Methods

### Amino acid preferences near intron–exon junctions.

We established a dataset of 178,382 human exons derived from the RefSeq track at the University of California Santa Cruz genome browser (http://genome.cse.ucsc.edu/cgi-bin/hgTables), March 2006 release. We obtained a set of 21,990 RefSeq files with the exon structure of the CDS specified. All files were checked to ensure that the coding sequence started with ATG, finished with a stop codon, had no internal stop codons, had no codons of uncertain translation, and was a multiple of three. This resolved to a dataset of 19,384 RefSeq files. We eliminated all first and last exons, leaving a sample of 178,382 exons. We trimmed all exons so that the first base was the first base of the first complete codon, and the last base the last of the final complete codon. As, to ensure correct splicing, first and last codons are by necessity highly skewed in usage, these too were eliminated. For each codon and in turn each amino acid, we considered proportional usage of that amino acid at a given distance from the junction both 3′ and 5′. All exons were divided in two, so a given codon never featured in both 3′ and 5′ calculations. This sample was not purged for duplicates. However, we repeated the analysis on a more stringently defined set of over 2,000 genes and 14,000 exons, previously purged for duplicates [21]. We confirmed that all qualitative trends are identical (data not shown).

We then considered the trend in usage of each amino acid as a function of the distance from the boundary. This we did by calculating Spearman rank correlations (rho) between the distance from the boundary (5′ or 3′) and proportional usage of the amino acid (i.e., in proportion to the number of residues at that given distance). Note that a negative rho implies an amino acid that is preferred near boundaries, and a positive rho implies a tendency to be avoided. To simplify numbering on the plots, we refer to amino acid positions by reference to the number of full codons between the given position and the relevant end of the trimmed exon. We split the three 6-fold degenerate amino acids into a block of four and a block of two. The block of two is specified by the usage of the lowercase letter (i.e., “S” implies TCA, TCC, TCG, and TCT, while “s” implies AGC and AGT). In relevant circumstances, the 2-fold and 4-fold blocks were treated as separate amino acids. Changes between the 2- and 4-fold blocks were not, however, treated as nonsynonymous changes.

### Mouse–human orthologous exon set.

As with the derivation of the human exon set, we obtained a set of mouse exons via the RefSeq track at the University of California Santa Cruz genome browser. For analysis of trends in amino acid preference near junctions, these exons were handled as described above. For analysis of orthologous exons, we obtained the human–mouse orthologue list from Mouse Genome Informatics (ftp://ftp.informatics.jax.org/pub/reports/index.html). We identified all pairs for which both mouse and human sequence had a RefSeq entry. As before, we eliminated all full coding sequences that were not well translated (more than one stop, ambiguous codons, etc.). We further eliminated those in which the number of exons differed between the orthologues. We then compared the phases of the putatively orthologous exons. Gene pairs in which any orthologous exon did not have the same phase in mouse and human were eliminated, leaving 7,767 genes. Any genes in which any orthologous exon differed by more than 5% in size were also eliminated, leaving 5,057 genes. First and last exons were removed, and all remaining orthologous exons were trimmed to start at the first full codon and end at the end of the last complete codon. They were then aligned at the peptide level using muscle v3.6 [31]. This left 36,683 aligned orthologous internal exons.

### HPI.

Burge and colleagues have characterised numerous hexameric sequences that function as splice enhancers [22,25,32,33]. For each hexamer we can then define a series of full codons that could potentially be present in the hexamer. If we consider a series of six nucleotides, n_1_n_2_n_3_n_4_n_5_n_6_, then codons n_1_n_2_n_3_, n_2_n_3_n_4_, n_3_n_4_n_5_, and n_4_n_5_n_6_ are specified in their entirety. We sum all such possible codons for all specified splice-enhancer hexamers. This provides a measure of ESE hexameric involvement of all possible codons, within any given hexamer dataset. The three stop codons were removed, and the proportions renormalised. To provide a metric of involvement of an amino acid in ESEs, we compared rates of involvement of codons in the hexamers with those in the genome as a whole. To this end, we normalised (after stop codon removal) the relative abundances of all codons as specified in the appropriate codon usage database (http://www.kazusa.or.jp/codon). We then generated 10,000 sets of random hexamers, each set being the same size as the input hexamer list. Hexamers were generated by joining two codons selected at random in proportion to their frequency in the appropriate genome. We parsed each random hexamer in the same manner as we parsed the input list, extracting all non-stop codons.

For each amino acid, given the frequencies of the relevant synonymous codons, we then determined the mean and standard deviation in relative abundance in the 10,000 random sets. The difference between the observed frequency of an amino acid in the real hexamer set and in the randomised sets, normalised by the standard deviation in the randomised sets, then is our HPI (i.e., a *Z* score). A high HPI value indicates that a given amino acid is enriched in ESEs compared with what is expected given its content in the genome, and given the underlying variance expected based on the number of hexamers used as input. Source code to calculate HPI is freely available from L. D. H.

In principle, the HPI score for an amino acid will change as a function of both input codon frequencies and with the input set of known ESE hexamers. In practice, we find that employing mouse rather than human codon frequencies makes little or no difference (data not shown). In this analysis we thus employed human codon frequencies to assemble random hexamers. As regards the input list for hexamers, we considered three sets: two sets specific to human 5′ and 3′ exonic ends (95 5′ enhancers and 177 3′ enhancers) and a set of 175 hexamers found both in mouse and human at either exonic end. We found that scores for 5′ and 3′ ends were very similar to each other. Unless otherwise stated, we employed the mouse–human conserved set. Use of this latter set is advantageous as it is most probably enriched for strong enhancers.

The splice-enhancing hexamers in all datasets have two striking features, notably an abundance of adenine and a dearth of cytosine, relative to their usage in the human genome. In the human genome, cytosine constitutes 26.0% of all nucleotides in coding sequences (derived from table of codon usage as noted above) but only 12.5% in splice enhancers, while adenine is 25.6% of all nucleotides in coding sequences but is 49.0% of the nucleotides in splice enhancers. Guanine is used in approximately the same amount in hexamers and in the genome (26.4% in genome and 25.7% in hexamers). Thymine is, like cytosine, underused in hexamers (12.4%), but its usage in the genome is just 22.0%, so its relative reduction in hexamers is less dramatic than that of cytosine. As expected, amino acids with few cytosine nucleotides in their codon set and many adenine residues tend to have positive HPI values (Spearman rank correlation, HPI versus cytosine content of codons, rho = −0.63, *p* = 0.0012; HPI versus adenine content of codons, rho = +0.71, *p* = 0.0002, *n* = 23). A composite measure of adenine and cytosine bias of codons (frequency of adenine in synonymous codon set minus frequency of cytosine) is a good predictor of HPI (Spearman rank correlation = 0.85, *p* < 0.0001, *n* = 23).

For the DPI pertinent to splice suppressors we extracted the 131 decamers provided by Wang et al. [29] from http://www.cell.com/cgi/content/full/119/6/831/DC1. The protocol to define DPI scores was identical to that to calculate HPI, except that random decamers were made by random selection of four codons and trimming off of the final two bases. The eight full codons in the decamers were employed to define expected frequencies.

### Establishing a set of ancient functional retroposed genes.

Mouse retroposed gene copies were identified using the procedure described in Vinckenbosch et al. [34]. For humans, we used a previously established retrocopy dataset [34]. To identify orthologous retrocopies shared between humans and mouse, we used human–mouse chained alignments available from the University of California Santa Cruz (hg17 versus Mm6). Similar to our previous procedure [34], we first extracted the best alignments that overlapped with the genomic location of human retrocopies and that were >15 kb (this length ensures that the alignment also covers surrounding, nonretrocopy-derived sequences in the two species). If no such alignments could be identified, presence/absence in mouse was not determined. We then scanned the chained alignments for an aligned block (putative orthologous sequence in the chain) that overlapped with the human retrocopy. If such a block was found, its corresponding mouse coordinates were compared to the mouse retrocopy set. Mouse retrocopies overlapping with these coordinates were considered orthologues of human retrocopies. In total, we identified 56 orthologous retrocopy pairs, of which 49 showed intact open reading frames in both species. The fact that these retrocopies emerged in the common ancestor of humans and mice (at least approximately 75–90 million years ago) and possess intact open reading frames strongly suggests that they have been selectively preserved by natural selection. Thus, they likely represent functional retroposed gene copies (retrogenes). Functionality of these human–mouse retrocopies is further supported by their generally higher transcription levels and lower *K*_A_/*K*_S_ values relative to younger, lineage-specific retrocopies [34].

To infer retrogene-specific changes, the sets of four sequences were aligned at the protein level using Muscle [31]. The sequences were then cut into individual exons by reference to the human annotation of parental genes. Exons were trimmed so as to contain only complete codons. The 5′ end of the first exon and the 3′ end of the last exon were ignored. All sites in the amino acid alignment that specified the same amino acid in three of the four sequences but a different amino acid in the third were considered, by parsimony, to be informative. That is, if the two human sequences specify amino acid X, as does the mouse parent gene at a given position, while the mouse retrogene is amino acid Y, then an X→Y change is inferred to have occurred in the mouse retrogene. The total number of retrogene changes is simply the sum of those in the mouse and those in the human retrogene, employing this strict 3:1 criterion.

### Expression data.

Gene expression estimates were obtained from Su and colleagues [35], employing the March 2006 annotation (http://wombat.gnf.org/index.html). Mas5 files with Affymetrix present/absent calls were used. Human gene expression data were obtained by merging U133A and GNF1h chip datasets. In both mouse and human, average expression was obtained from samples of the same tissues. Probes matching to more than one gene were eliminated from further analyses. Indexes of gene activity were obtained only from samples obtained from normal adult tissues. Levels and breadth of expression were calculated. Three indexes for expression levels were obtained: peak, average, and median expression. The peak level was the highest score across all analysed tissues. Breadth of expression was calculated from present/absent calls. For the analysis of mean/median levels, for each gene we considered only those tissues in which a gene was expressed (judged by present/absent call). When multiple probes matched the same gene we considered a gene to be expressed in a given tissue if half or more of the probes indicated presence.

## Supporting Information

Figure S1 Trends in Relative Levels of Amino Acid Usage as a Function of the Distance from Intron–Exon Boundaries at Both 5′ and 3′ Ends (183 KB PDF)

Figure S2 Relationship between Slope of Regression Line (between Proportion of Amino Acid and Distance from Boundary) and HPI Score For (A) 5′ and (B) 3′ ends.
(47 KB DOC)

Figure S3 Rates of Evolution in Enhancer and Nonenhancer Domains as a Function of Distance from the Boundary for Exons Longer than 200 bp All exons contribute equally to all data points. Here we merge 3′ and 5′ data.
(55 KB DOC)

Figure S4 Plot of Standardised Residuals for the Regression of AC Content Versus rho Grey lines indicate top and bottom 95% confidence intervals.
(41 KB DOC)

Figure S5 Relationship between the Correlation between Proportion of Amino Acid and Distance from Boundary (rho) and the Mean of HPI and DPI For (A) 5′ and (B) 3′ ends.
(43 KB DOC)

Figure S6 Glycine Usage as a Function of Frame of Exon and Exonic End The first number in the title is the exonic end (5′ or 3′); the second is the phase (0, 1, or 2).
(115 KB DOC)

Figure S7 Periodicity Analysis of Glycine and Proline For (A) glycine and (B) proline the distribution of homologous residues in the flanking sequence was determined. The first such residue in the sequence was taken as the reference point 0; once frequency data for the same amino acid were obtained, for the 150 flanking residues, the reference point moved along to the next homologous residue. The frequency of the residue in the flanking sequence was then determined by the absolute occurrence of the residue at this distance, divided by the number of informative sites. Glycine exhibits an unusual pattern where, following the use of a glycine, there is a preference for glycine to be used every third amino acid (top series of points in [A]). This is not an artefact of contamination by collagen transcripts (GPXn), as the distribution of proline indicates no such trend. This pattern in glycine usage is still strong over 500 residues away from the reference point.
(79 KB DOC)

Table S1 Correlations and Partial Correlations between Rate of Protein Evolution (*K*_A_ or *K*_A_/*K*_S_), Proportion of Sequence within 50, 70, or 100 bp of an Intron–Exon Junction, and Measures of Expression of the Relevant Gene in Mouse (36 KB DOC)

## Abbreviations

DPI: decamer preference index
ESE: exonic splice enhancer
HPI: hexamer preference index
*K*_A_: the number of nonsynonymous substitutions per nonsynonymous site
*K*_S_: the number of synonymous substitutions per synonymous site
