# Supplementary material for: Splicing and the Evolution of Proteins in Mammals
Source: PLoS Biol. 2007 Feb 6;5(2):e14. doi: 10.1371/journal.pbio.0050014 (PMC1790955; doi:10.1371/journal.pbio.0050014)
Supplement: Supplementary file 1 [file pbio.0050014.sg001.pdf]

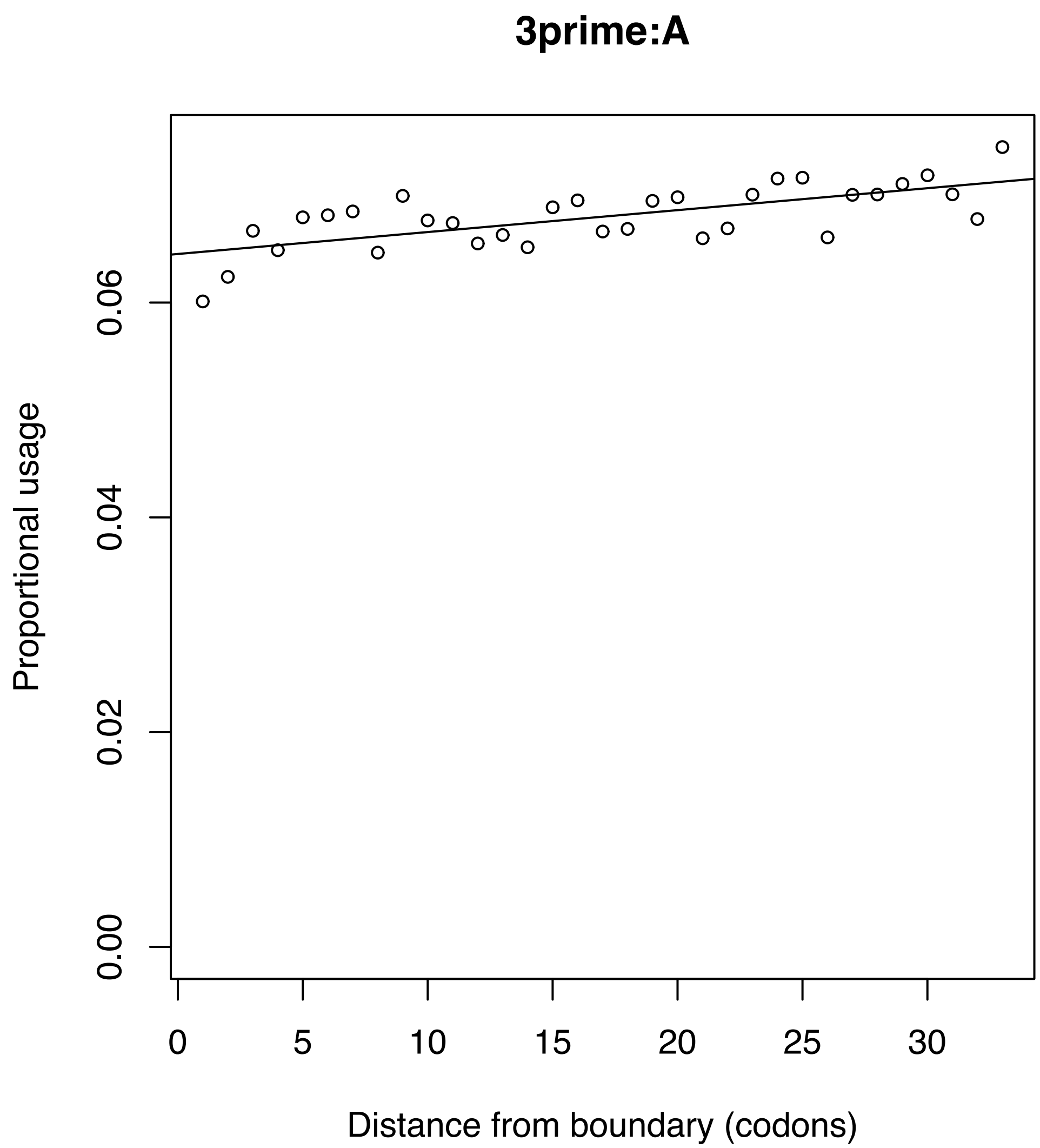
3prime:A
Proportional usage
0.00
0.02
0.04
0.06
0
5
10
15
20
25
30
Distance from boundary (codons)

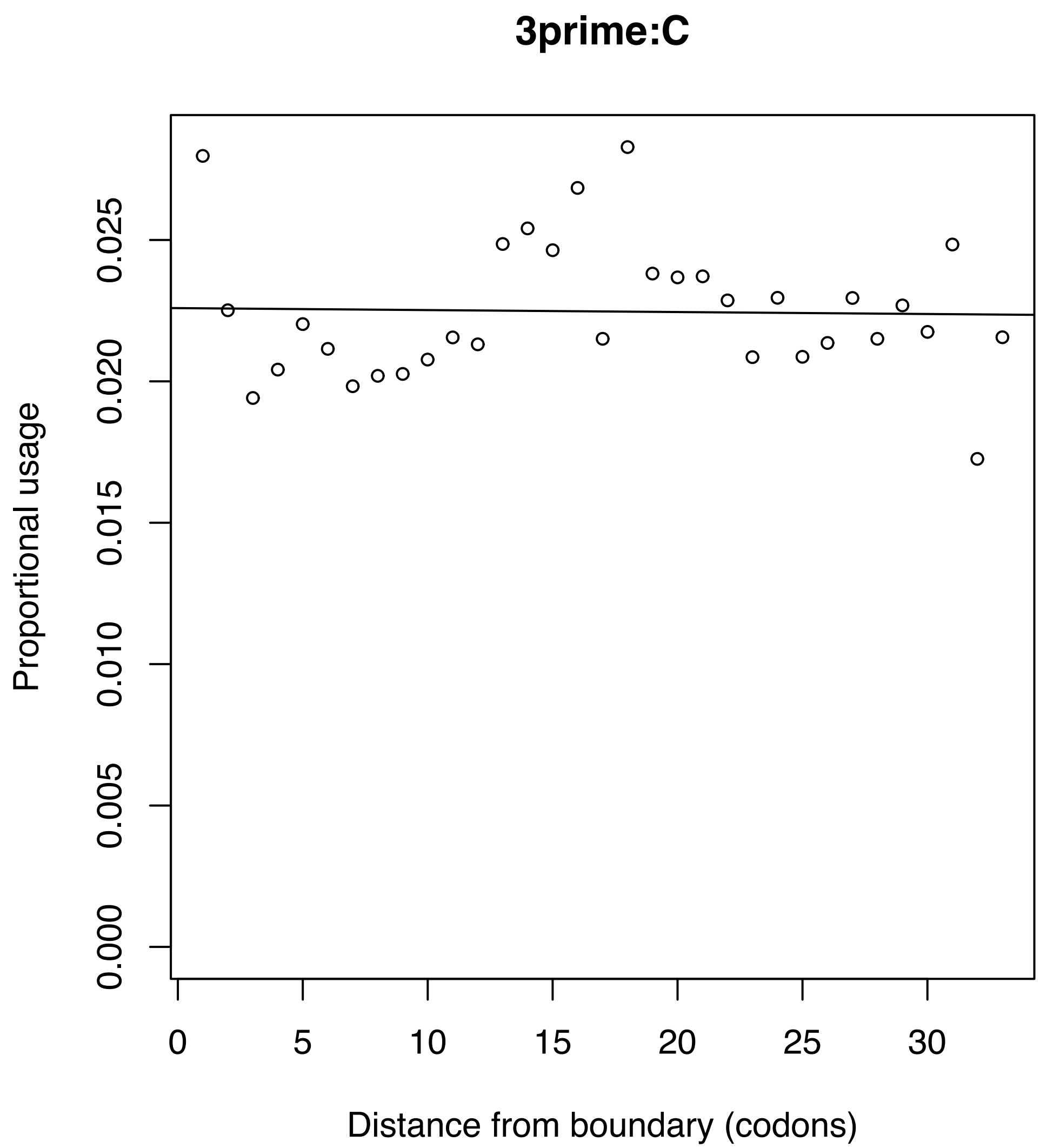
3prime:C
Proportional usage
0.000
0.005
0.010
0.015
0.020
0.025
0
5
10
15
20
25
30
Distance from boundary (codons)

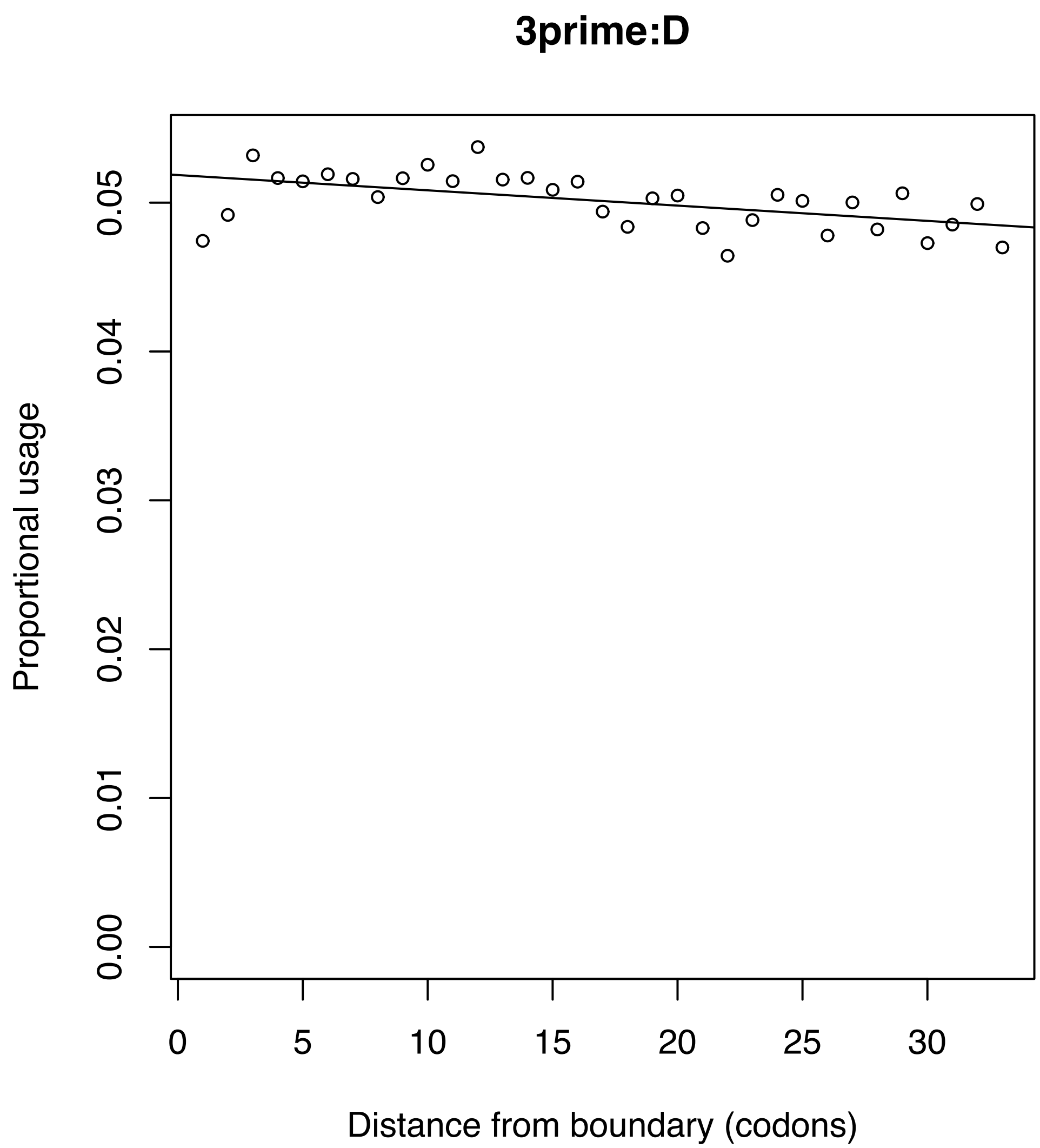
3prime:D
Proportional usage
0.00
0.01
0.02
0.03
0.04
0.05
0
5
10
15
20
25
30
Distance from boundary (codons)

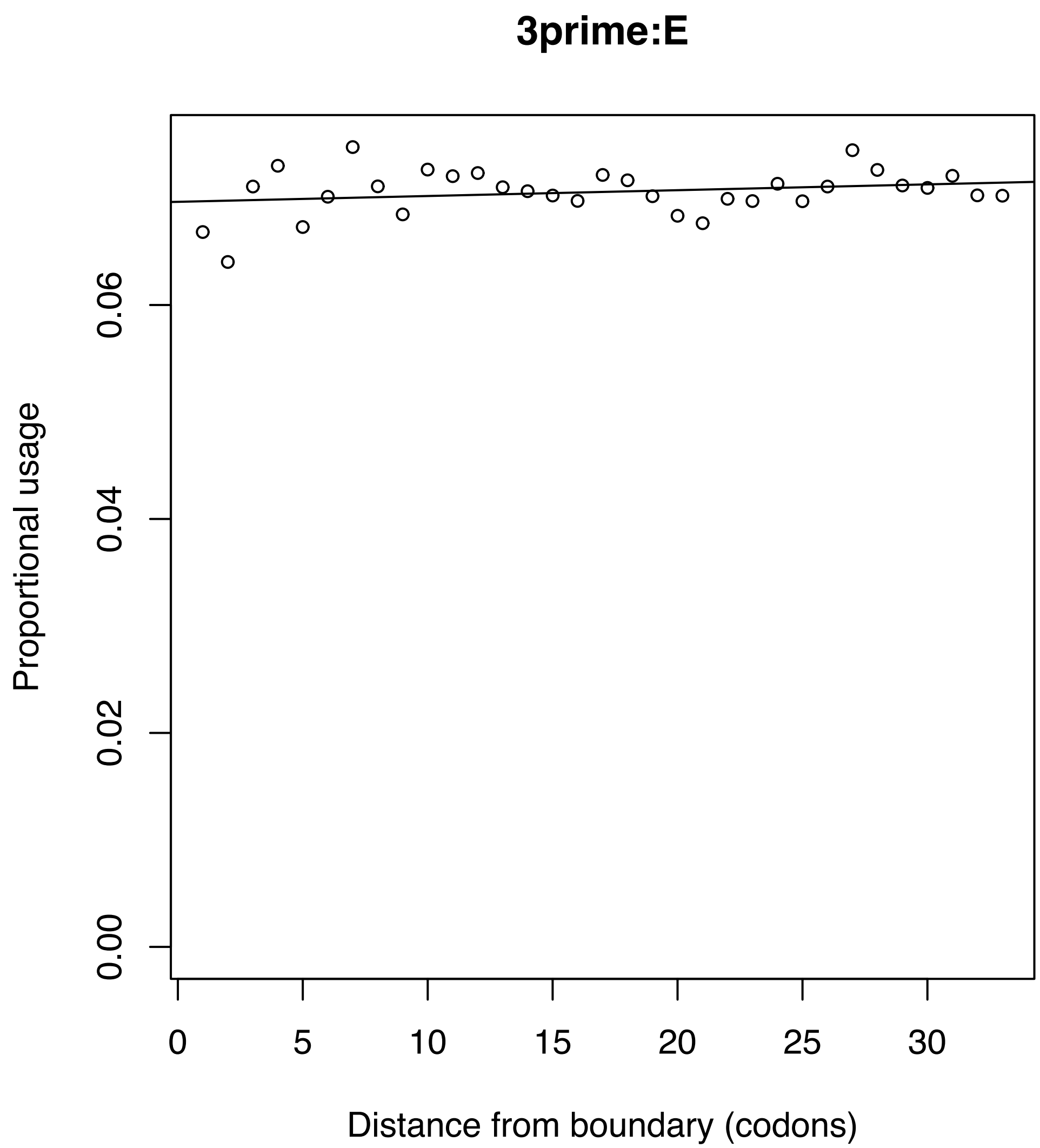
3prime:E
Proportional usage
0.00
0.02
0.04
0.06
0
5
10
15
20
25
30
Distance from boundary (codons)

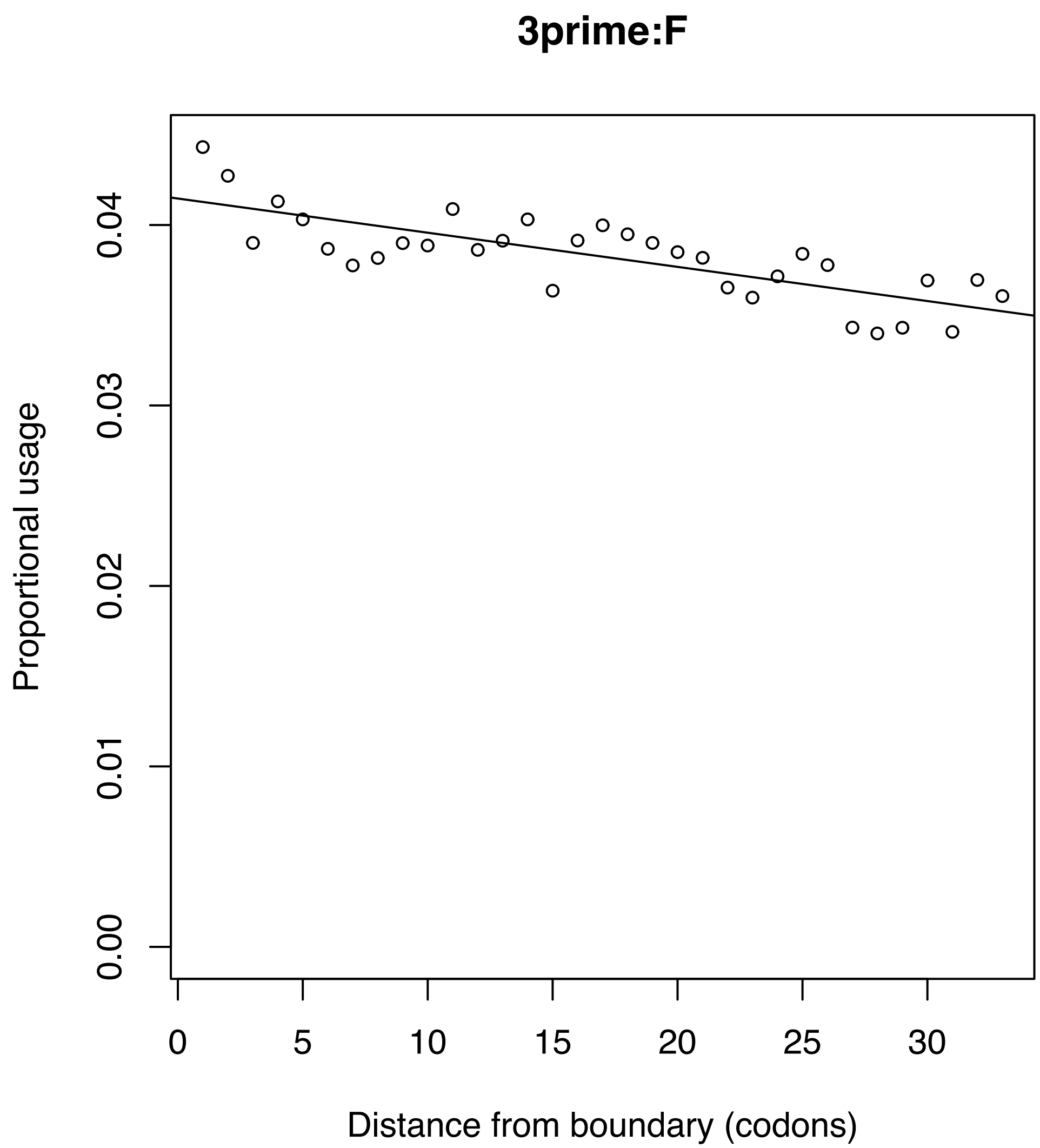

3prime:F
Proportional usage
0.00
0.01
0.02
0.03
0.04
0
5
10
15
20
25
30
Distance from boundary (codons)

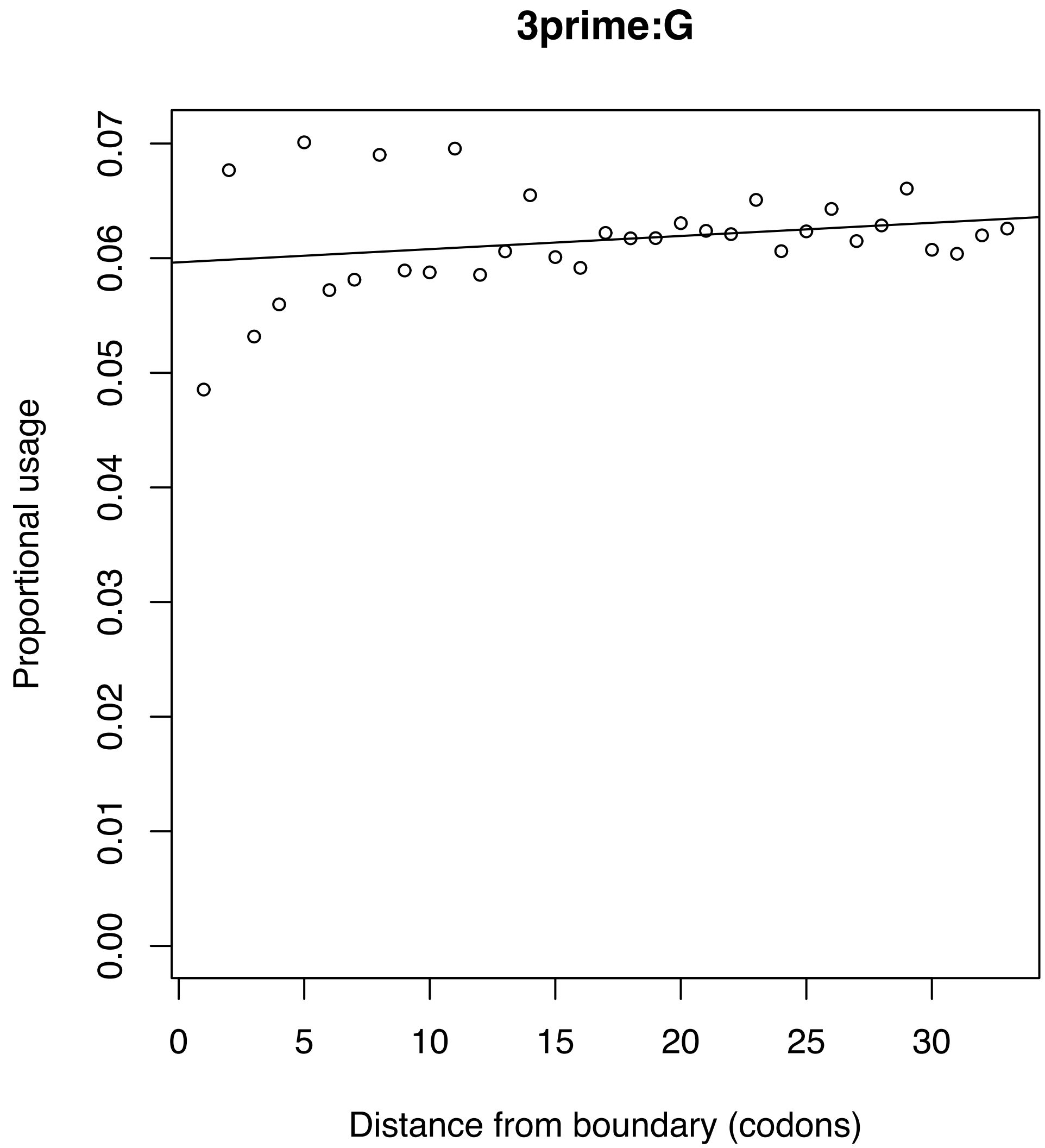
3prime:G
Proportional usage
0.00
0.01
0.02
0.03
0.04
0.05
0.06
0.07
0
5
10
15
20
25
30
Distance from boundary (codons)

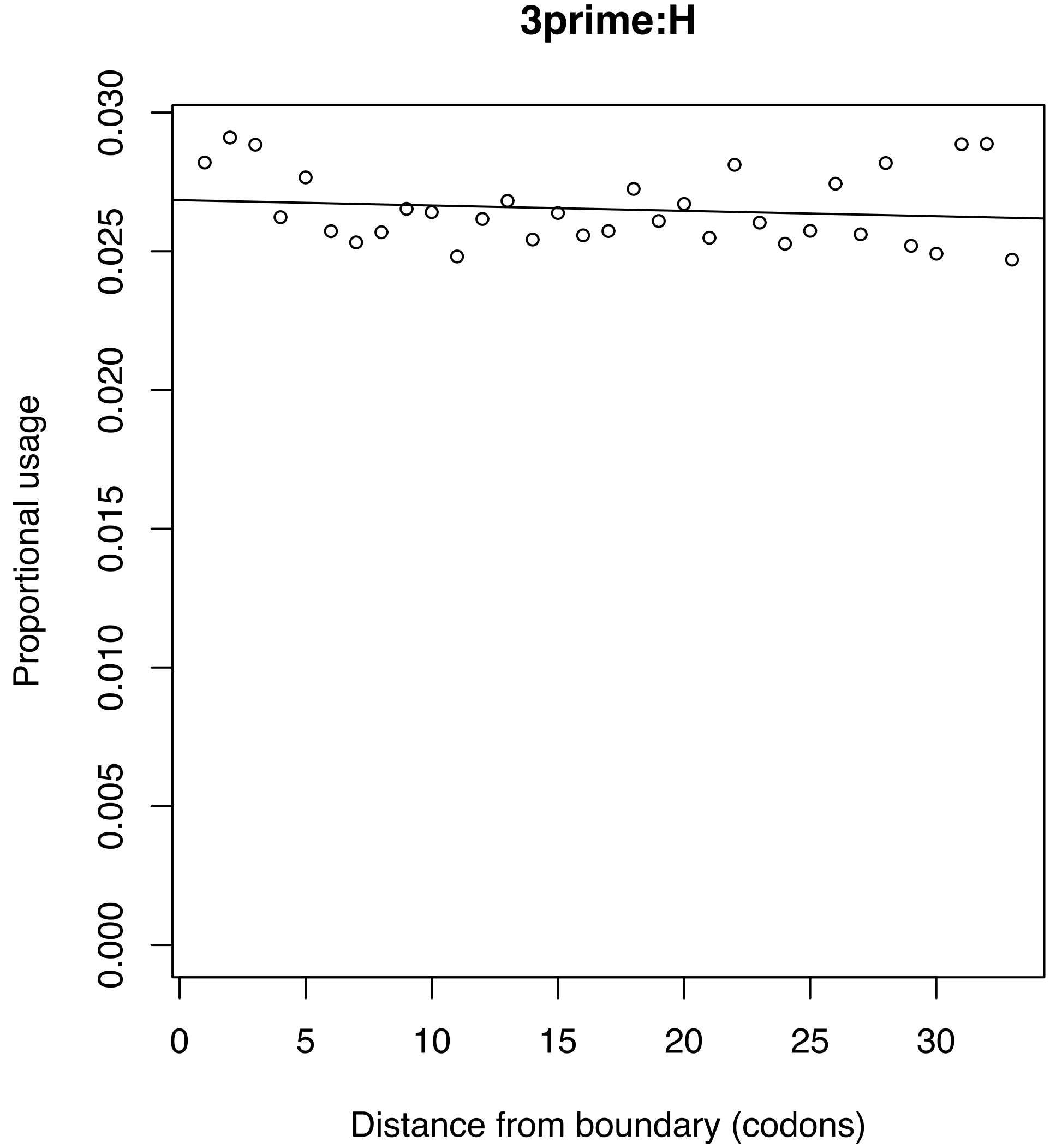
3prime:H
Proportional usage
0.000
0.005
0.010
0.015
0.020
0.025
0.030
0
5
10
15
20
25
30
Distance from boundary (codons)

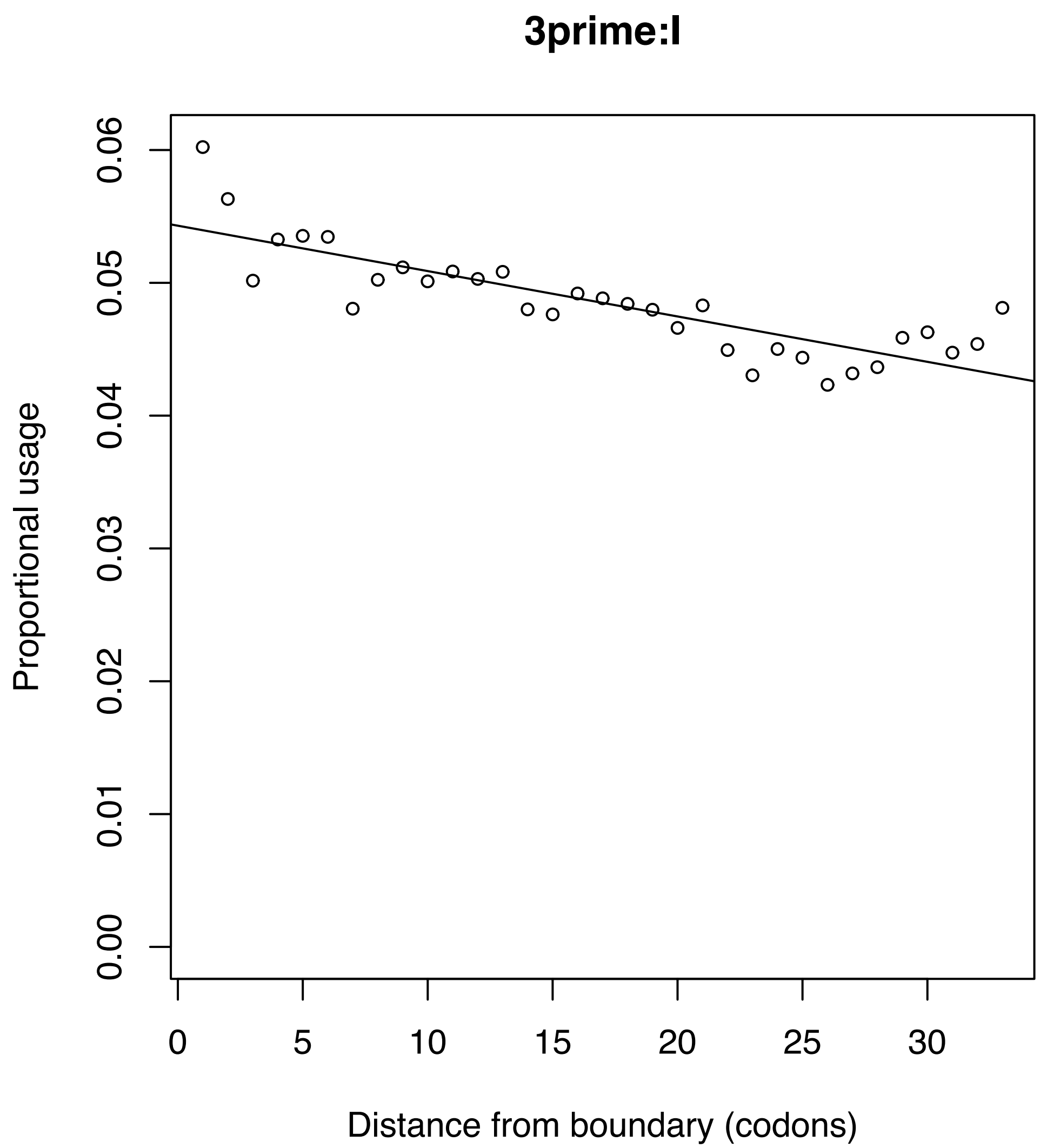
3prime:I
Proportional usage
0.00
0.01
0.02
0.03
0.04
0.05
0.06
0
5
10
15
20
25
30
Distance from boundary (codons)

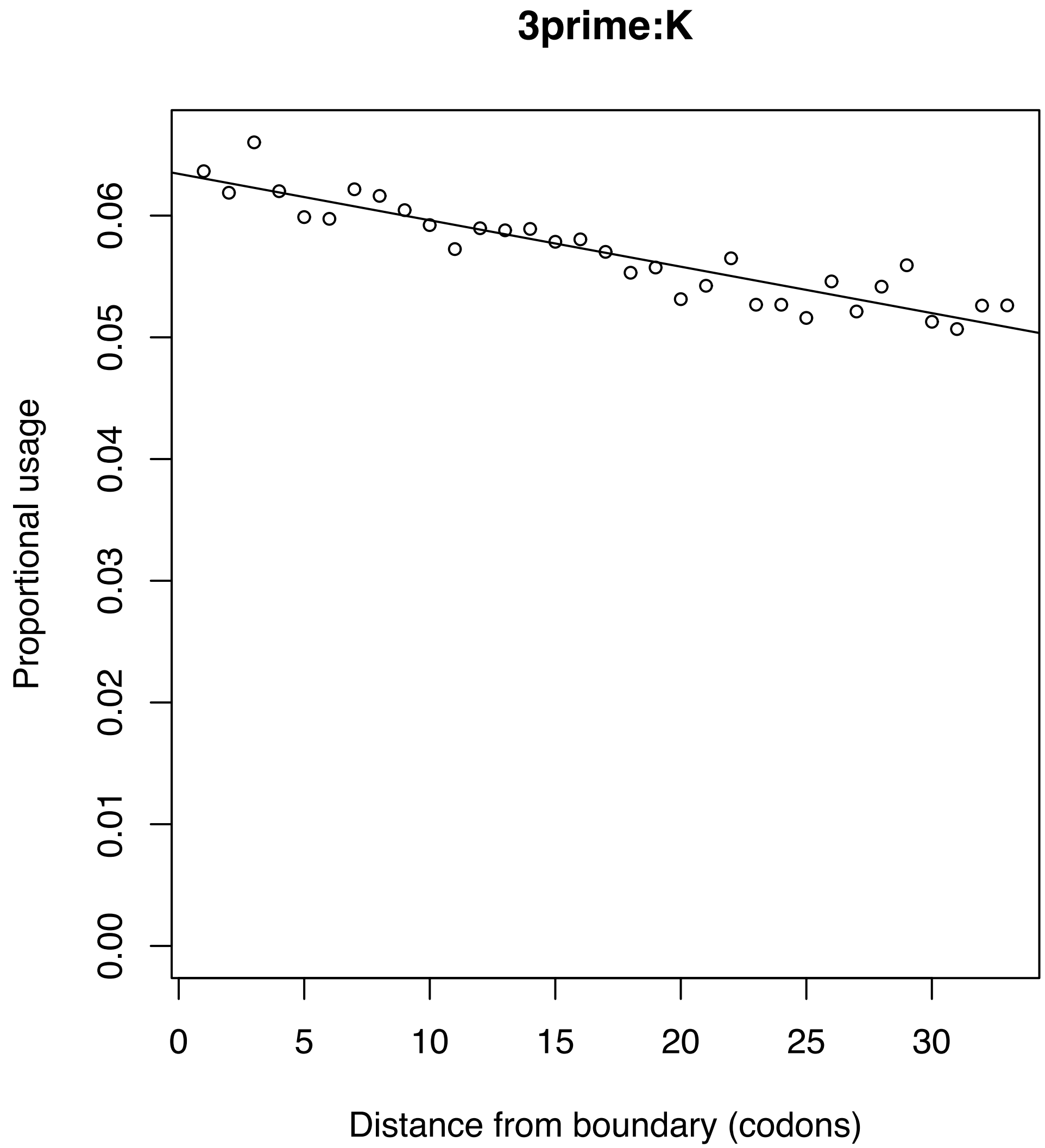
3prime:K
Proportional usage
0.00
0.01
0.02
0.03
0.04
0.05
0.06
0
5
10
15
20
25
30
Distance from boundary (codons)

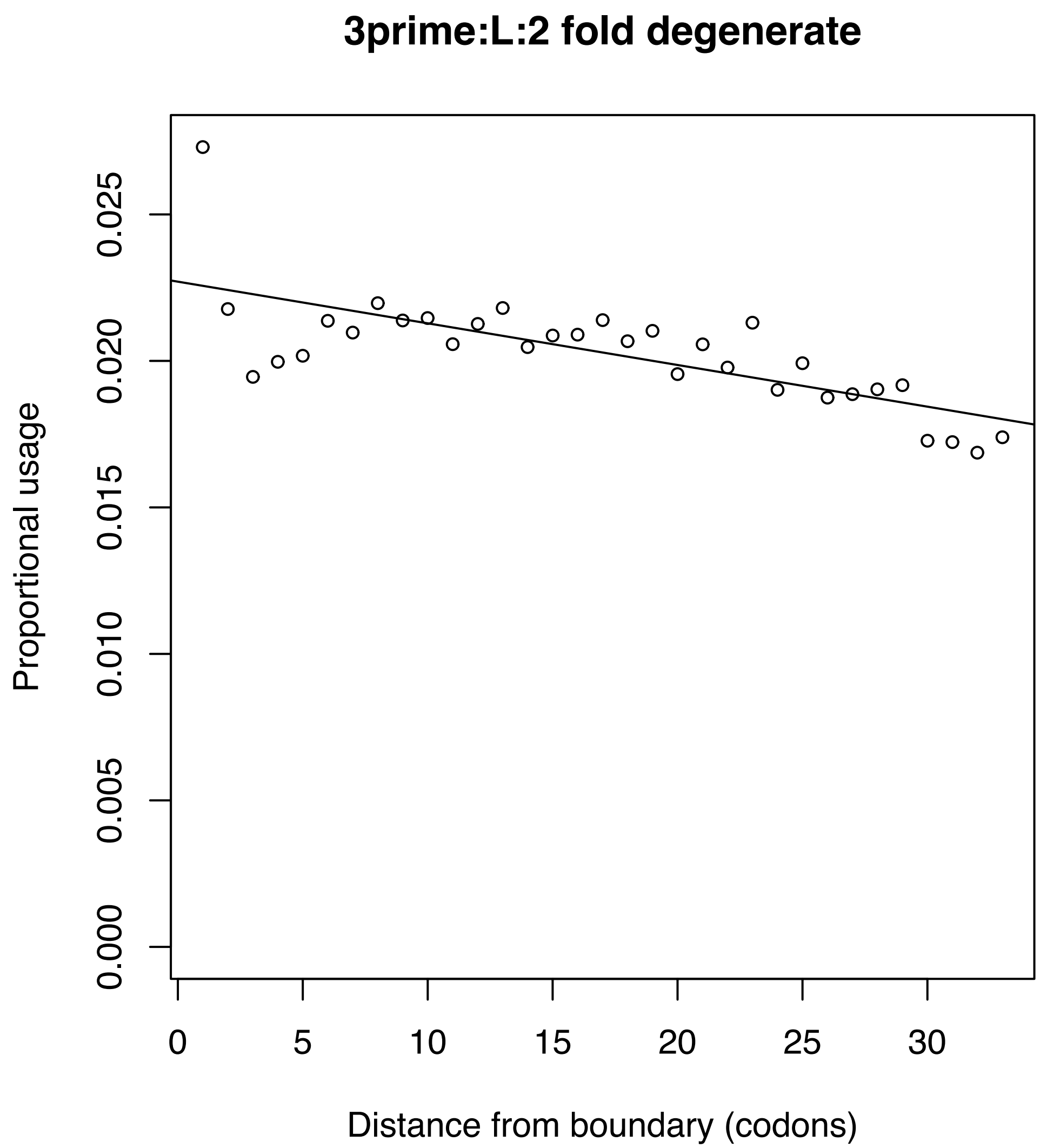
3prime:L:2 fold degenerate
Proportional usage
0.000
0.005
0.010
0.015
0.020
0.025
0
5
10
15
20
25
30
Distance from boundary (codons)

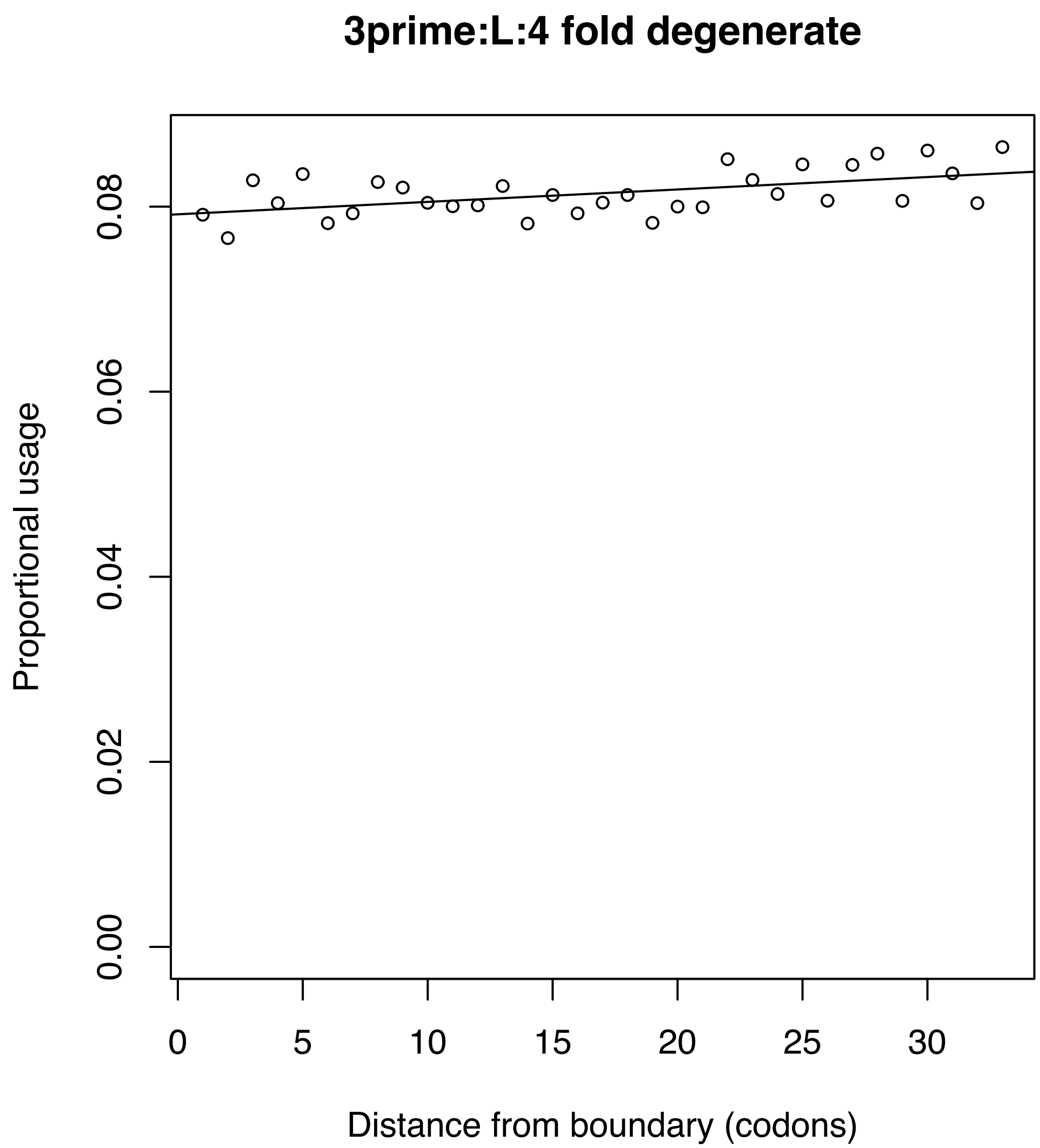
3prime:L:4 fold degenerate
Proportional usage
0.00
0.02
0.04
0.06
0.08
0
5
10
15
20
25
30
Distance from boundary (codons)

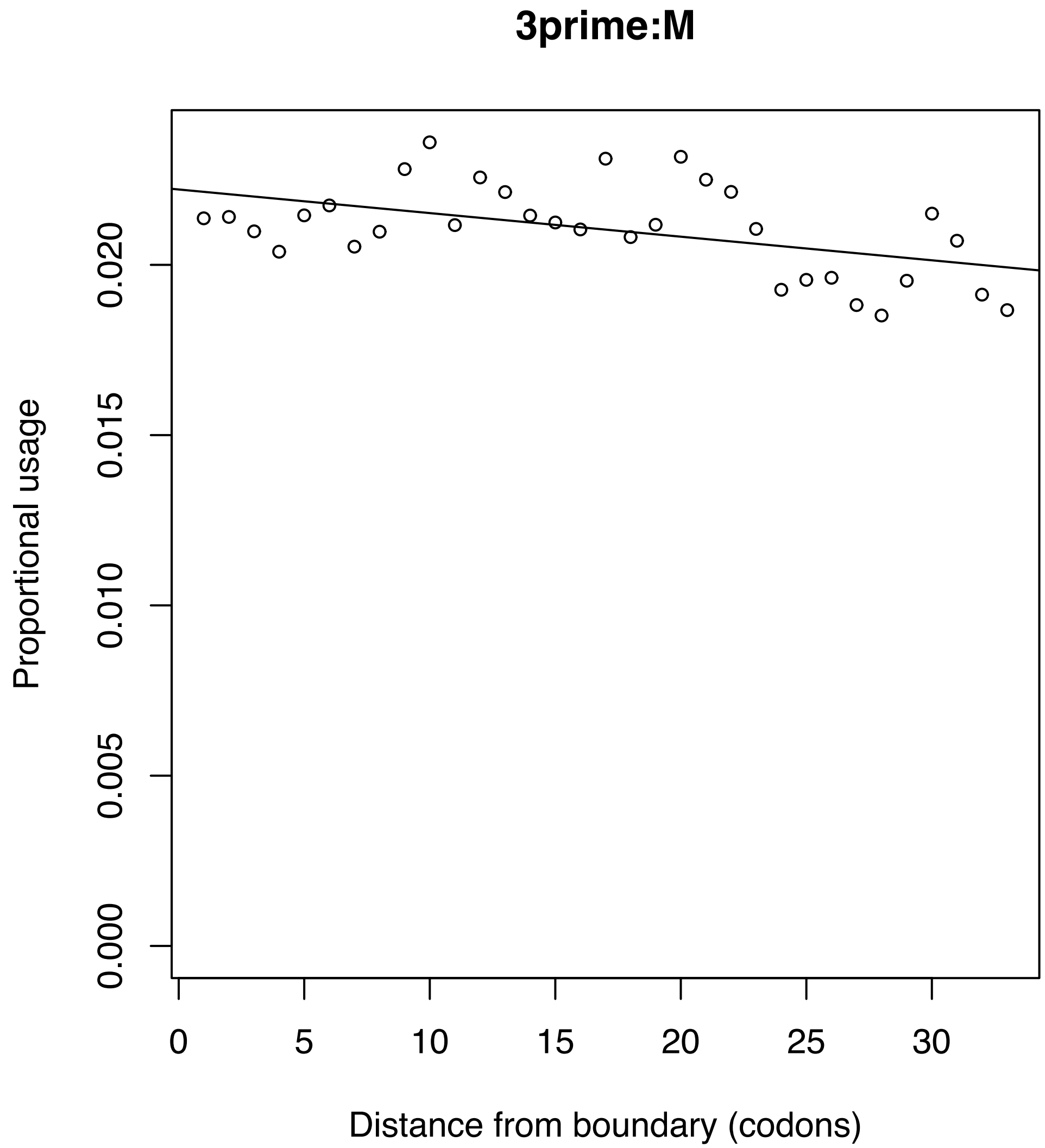
3prime:M
Proportional usage
0.000
0.005
0.010
0.015
0.020
0
5
10
15
20
25
30
Distance from boundary (codons)

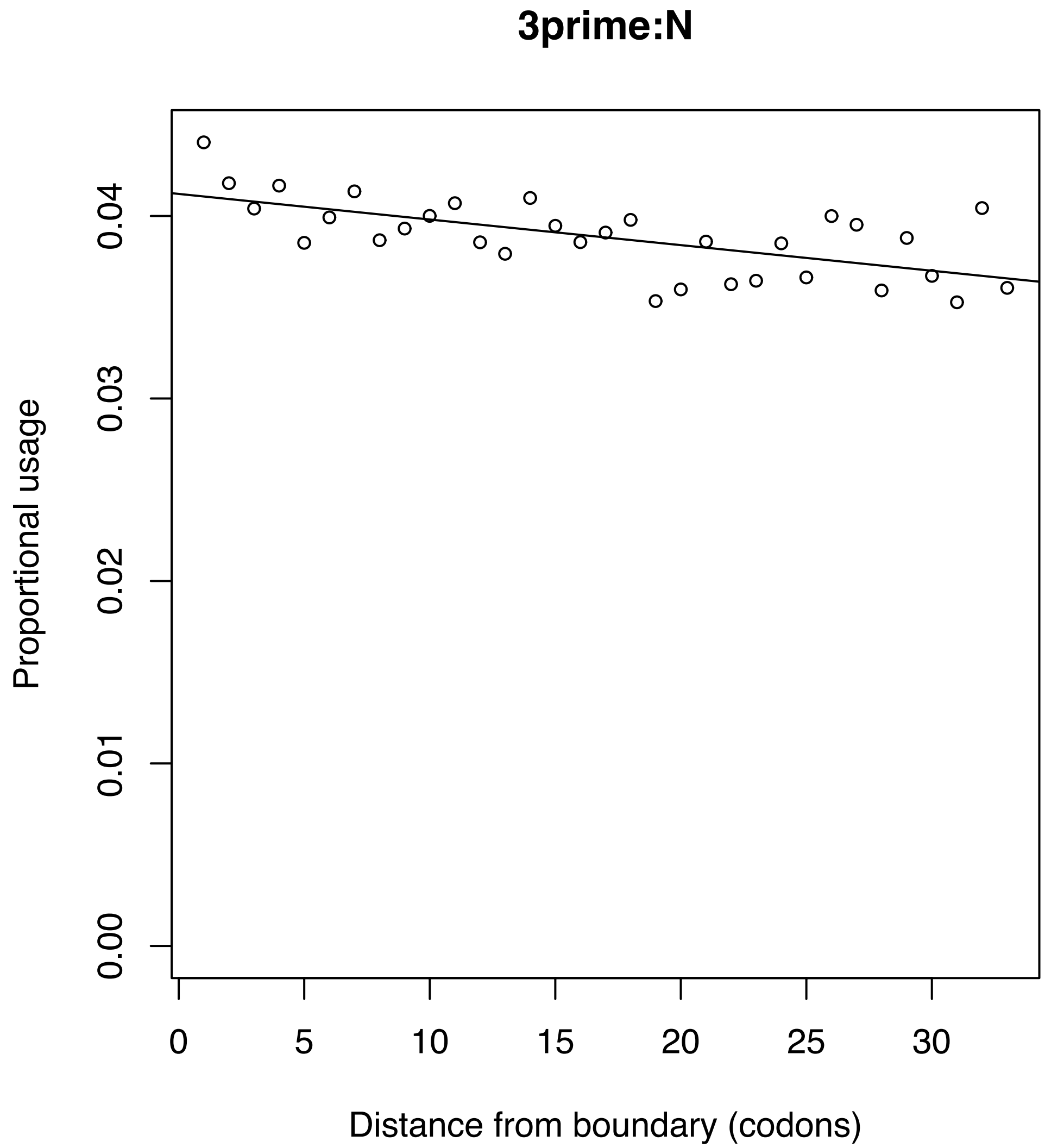
3prime:N
Proportional usage
0.00
0.01
0.02
0.03
0.04
0
5
10
15
20
25
30
Distance from boundary (codons)

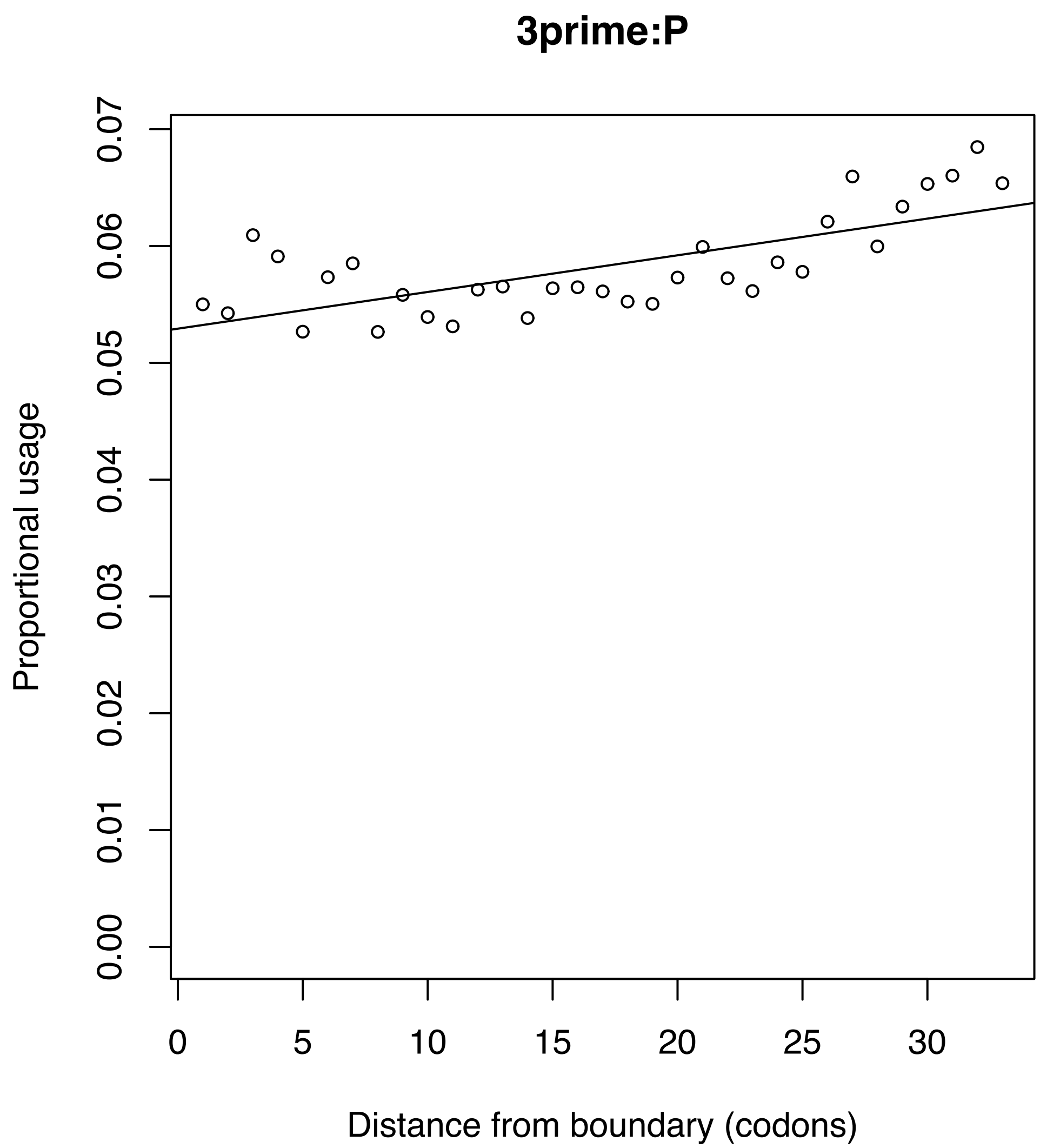

3prime:P
Proportional usage
0.00
0.01
0.02
0.03
0.04
0.05
0.06
0.07
0
5
10
15
20
25
30
Distance from boundary (codons)

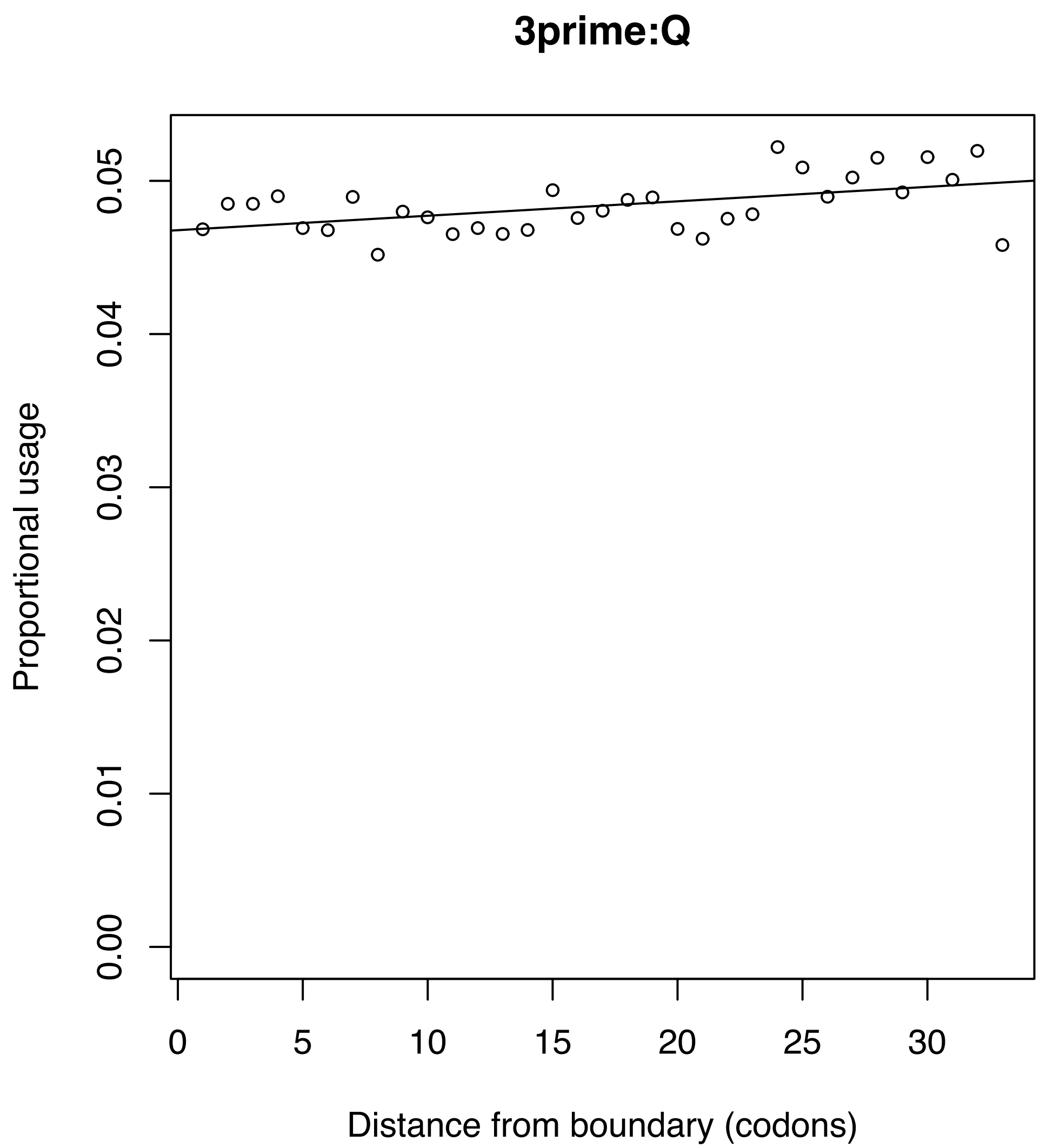

3prime:Q
Proportional usage
0.00
0.01
0.02
0.03
0.04
0.05
0
5
10
15
20
25
30
Distance from boundary (codons)

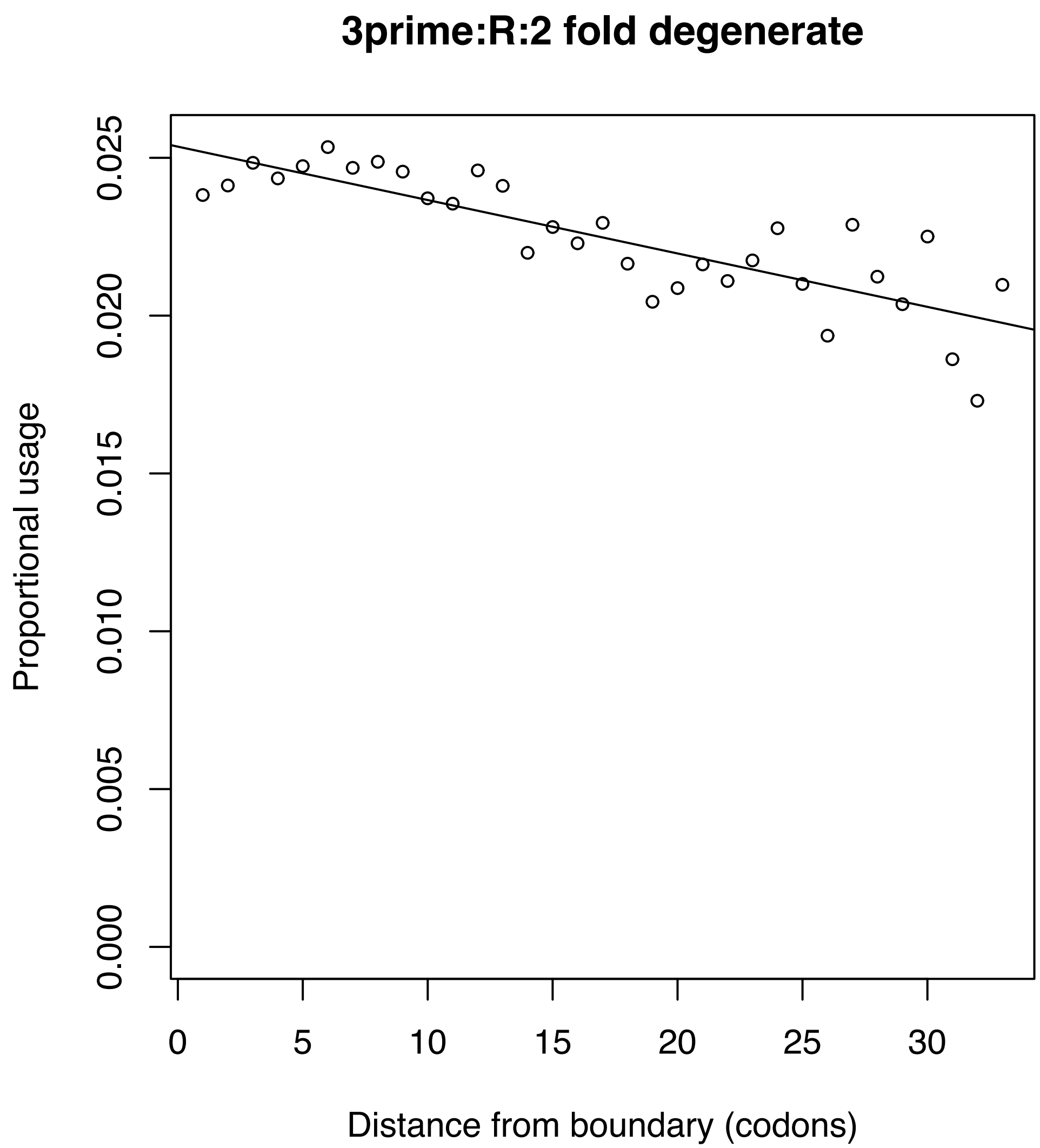
3prime:R:2 fold degenerate
Proportional usage
0.000
0.005
0.010
0.015
0.020
0.025
0
5
10
15
20
25
30
Distance from boundary (codons)

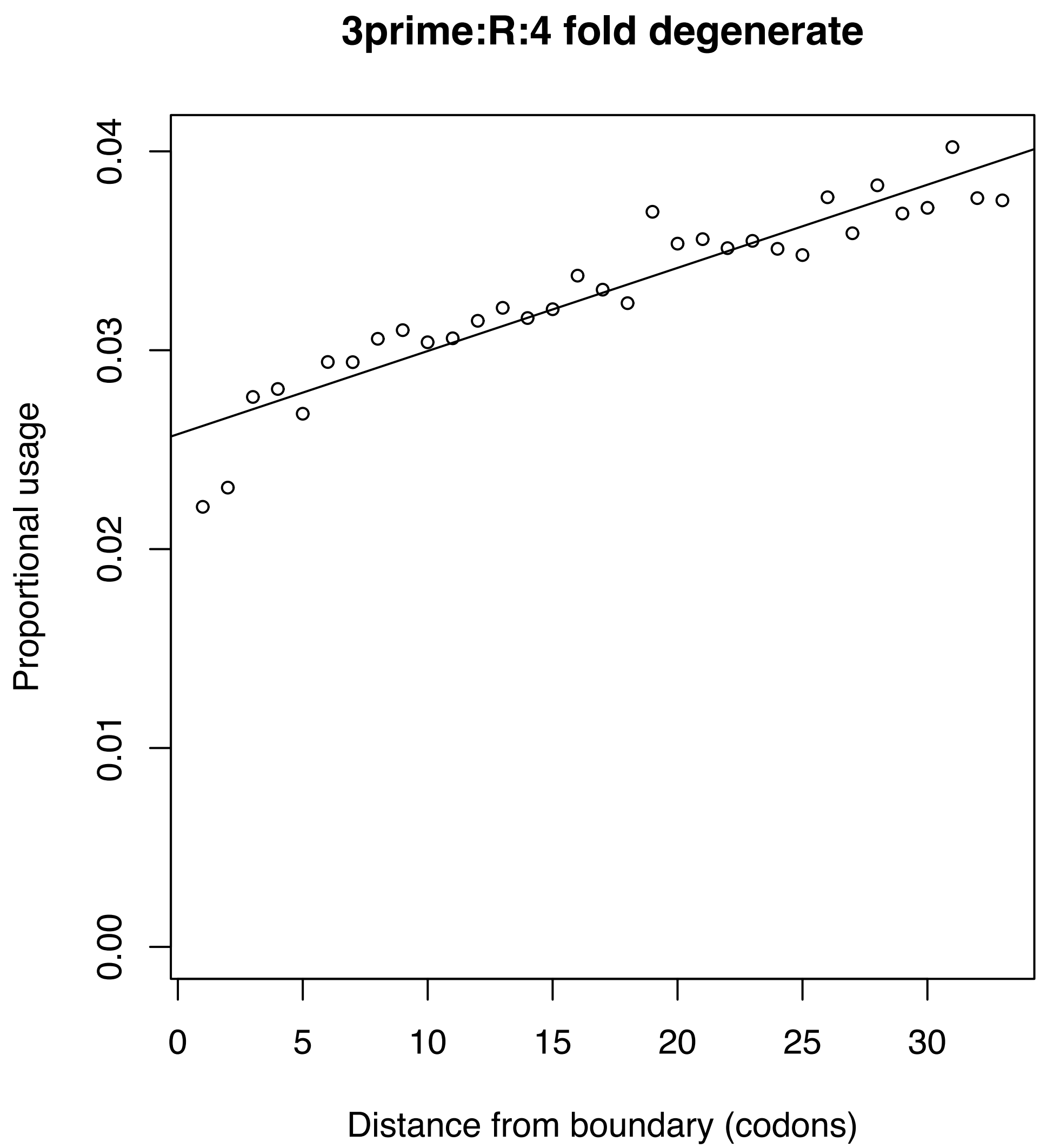
3prime:R:4 fold degenerate
Proportional usage
0.00
0.01
0.02
0.03
0.04
0
5
10
15
20
25
30
Distance from boundary (codons)

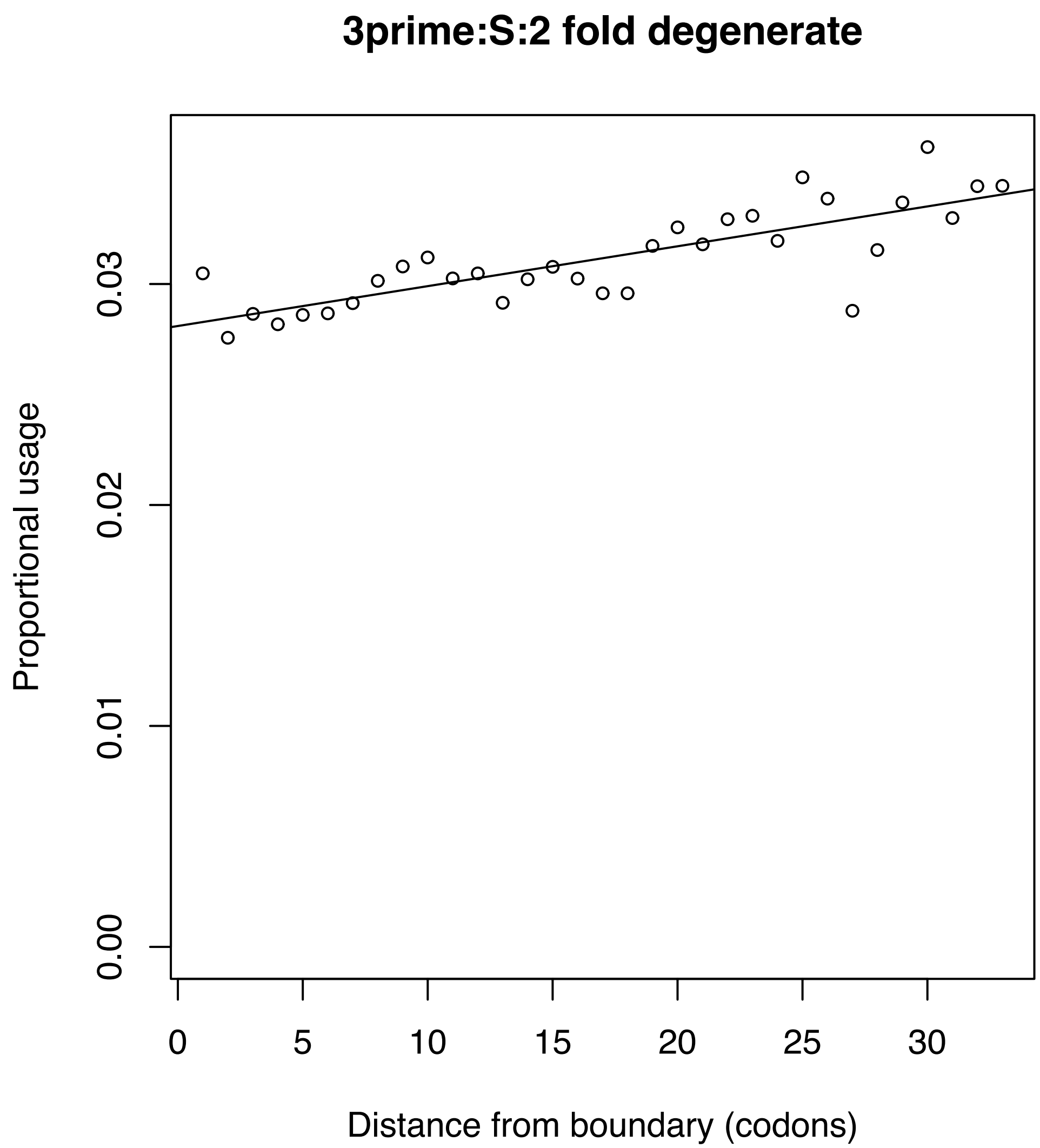
3prime:S:2 fold degenerate
Proportional usage
0.00
0.01
0.02
0.03
0
5
10
15
20
25
30
Distance from boundary (codons)

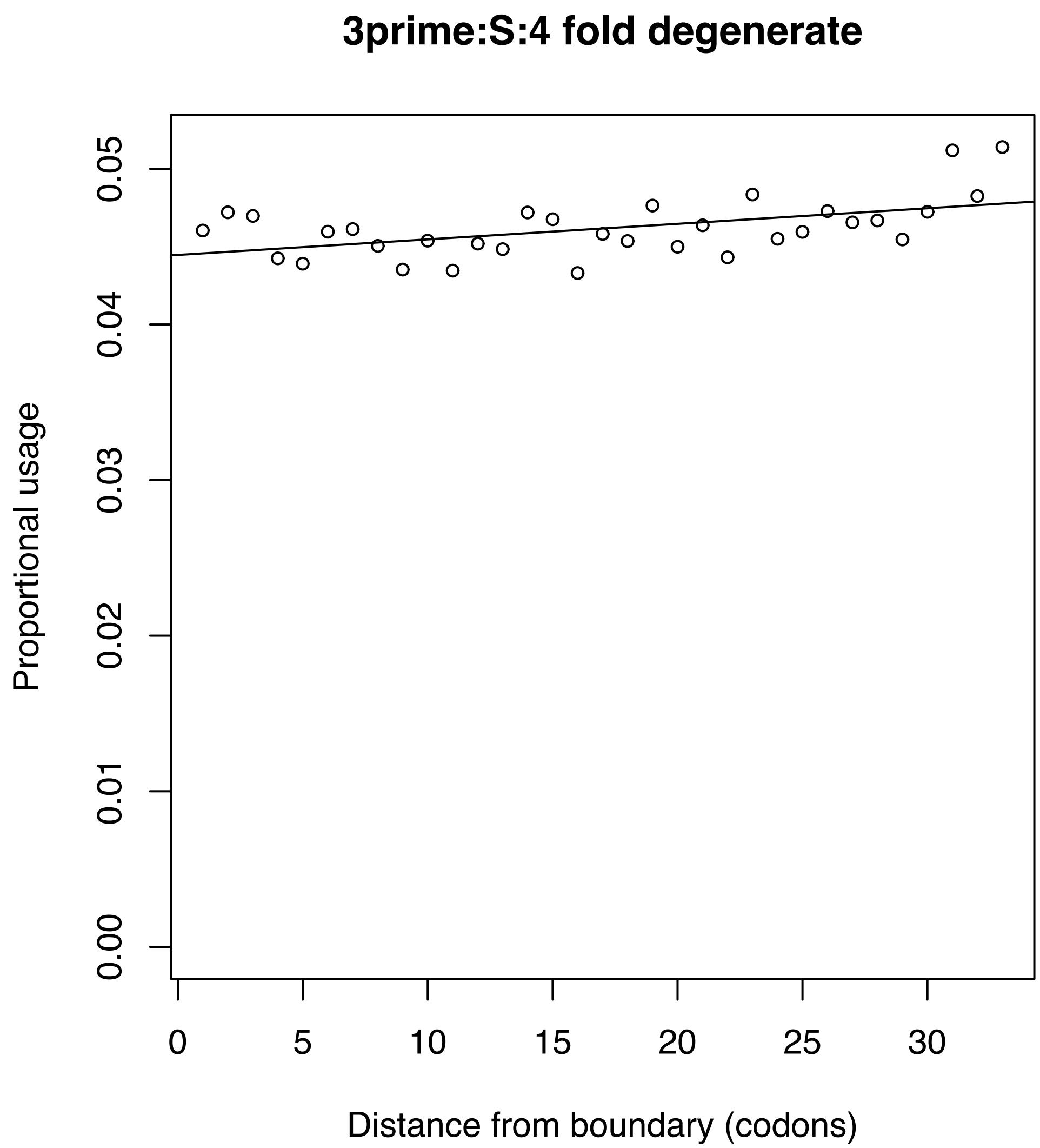
3prime:S:4 fold degenerate
Proportional usage
0.00
0.01
0.02
0.03
0.04
0.05
0
5
10
15
20
25
30
Distance from boundary (codons)

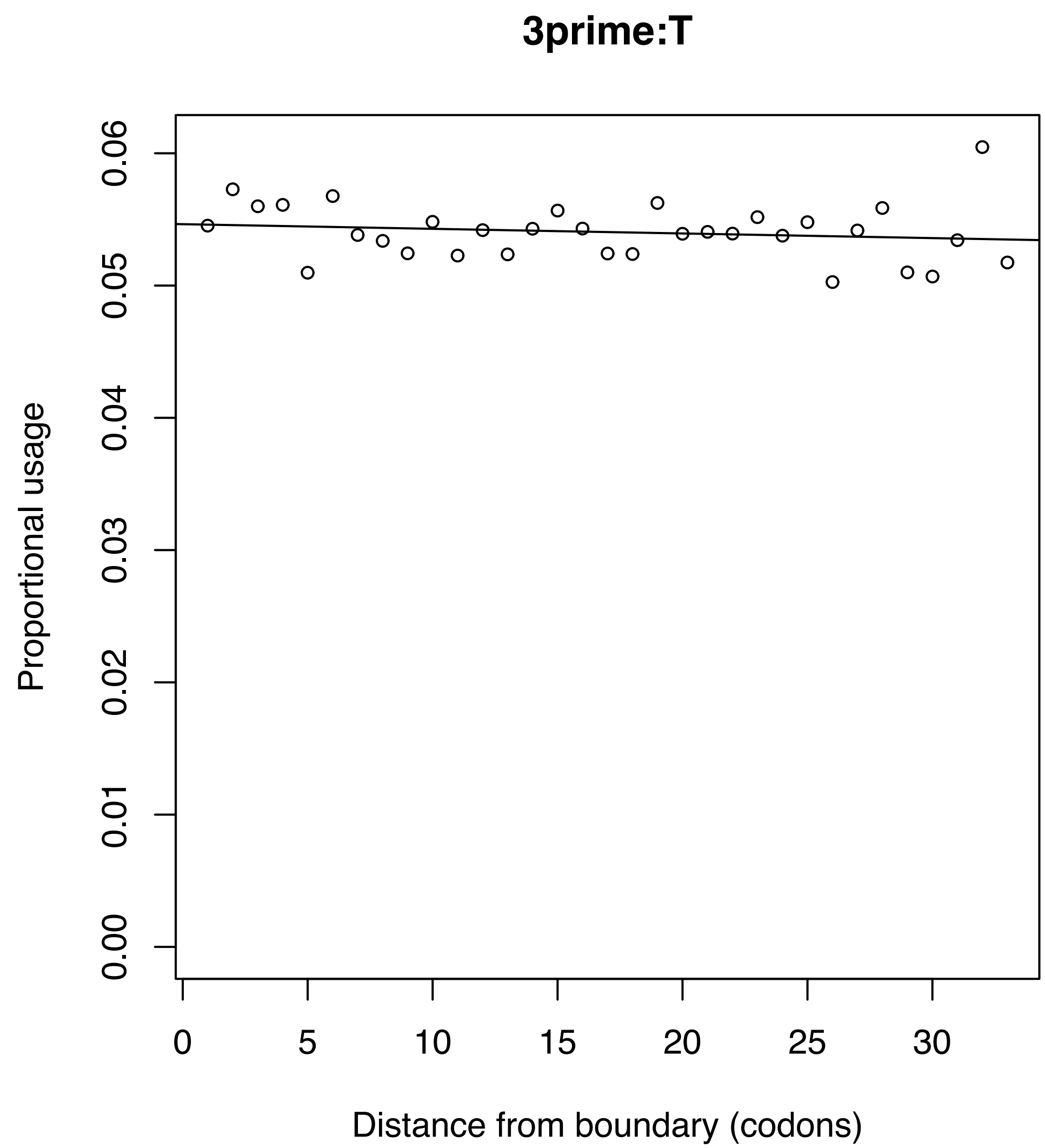
3prime:T
Proportional usage
0.00
0.01
0.02
0.03
0.04
0.05
0.06
0
5
10
15
20
25
30
Distance from boundary (codons)

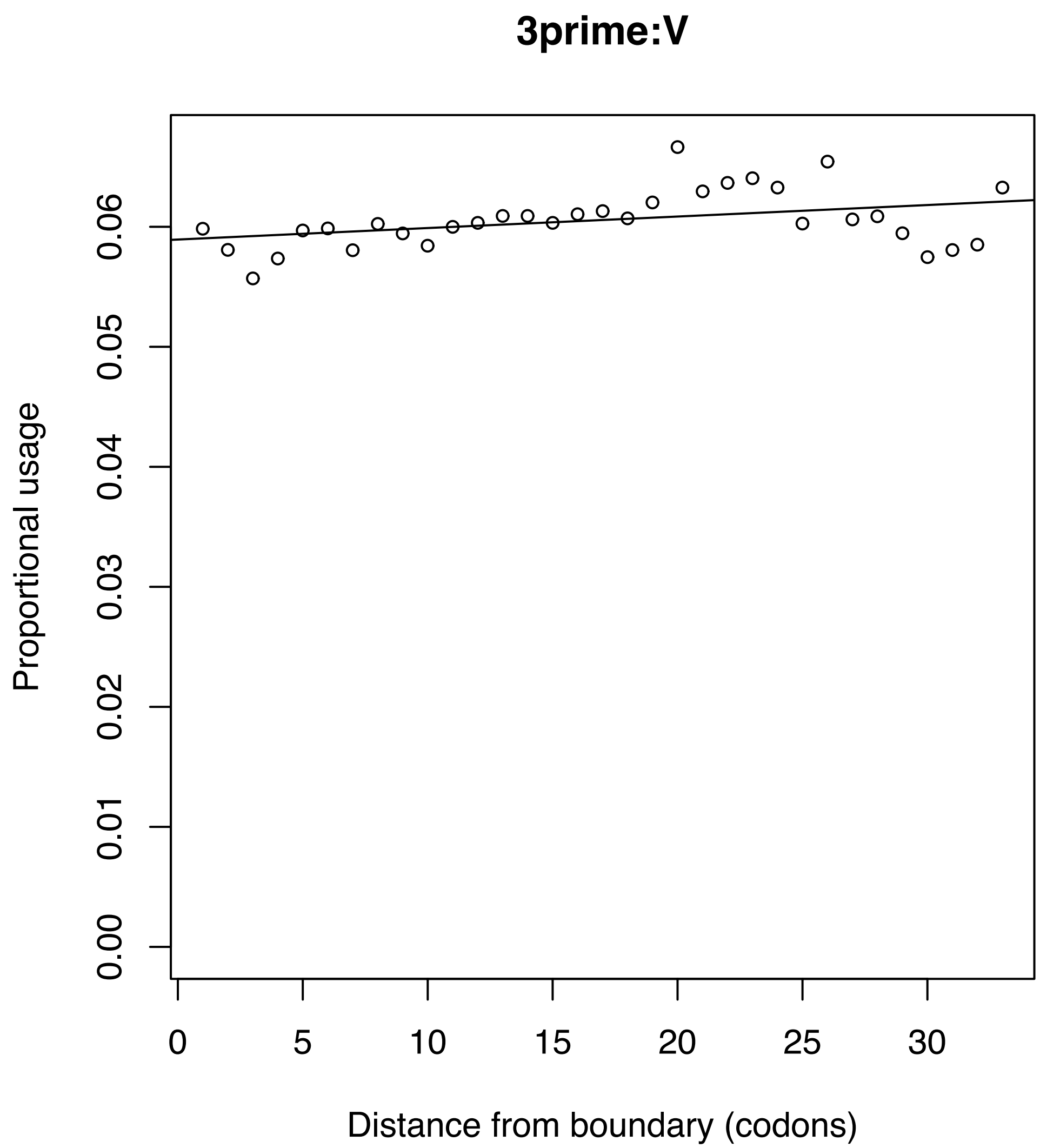
3prime:V
Proportional usage
0.00
0.01
0.02
0.03
0.04
0.05
0.06
0
5
10
15
20
25
30
Distance from boundary (codons)

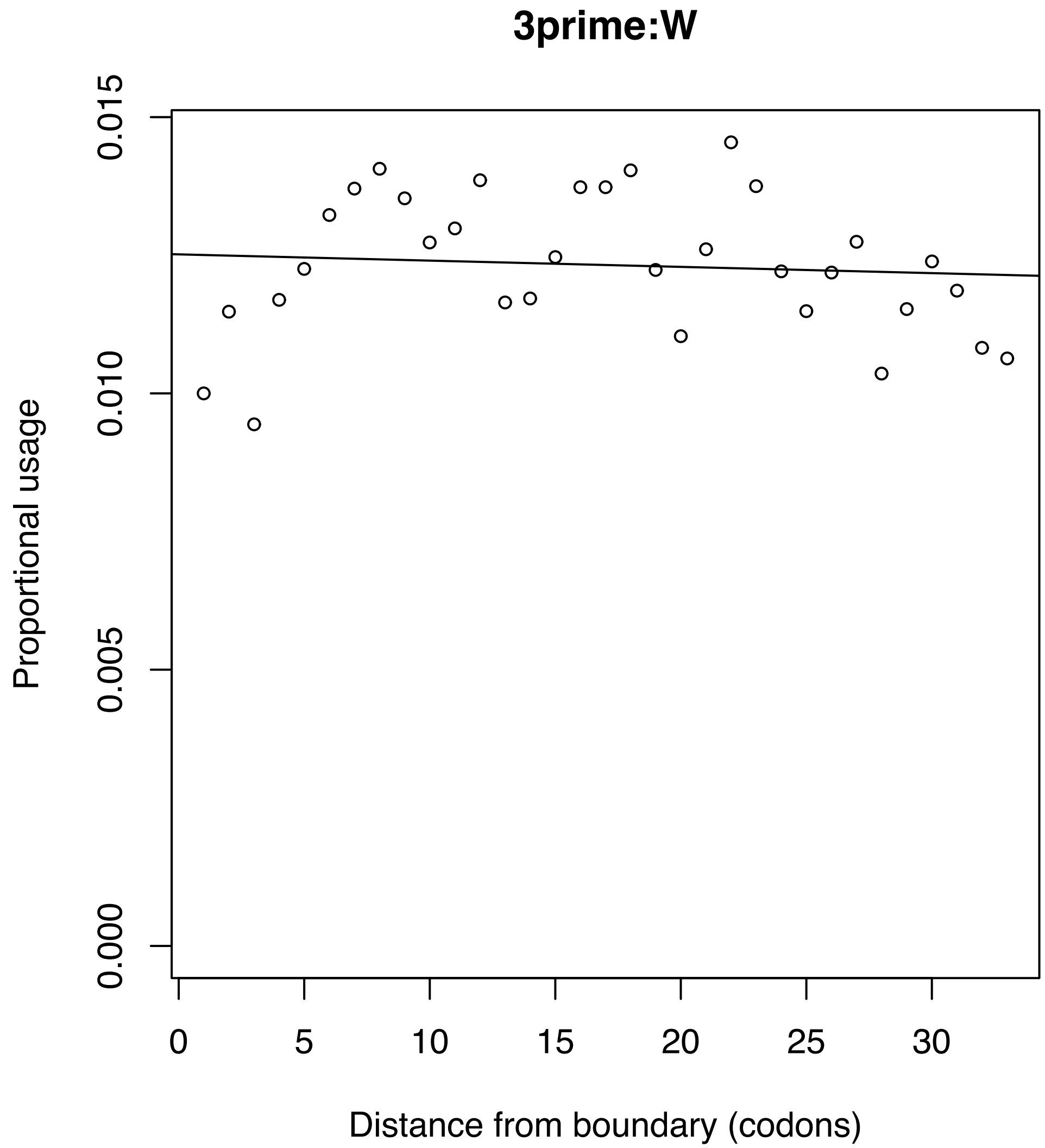

3prime:W
Proportional usage
0.000
0.005
0.010
0.015
0
5
10
15
20
25
30
Distance from boundary (codons)

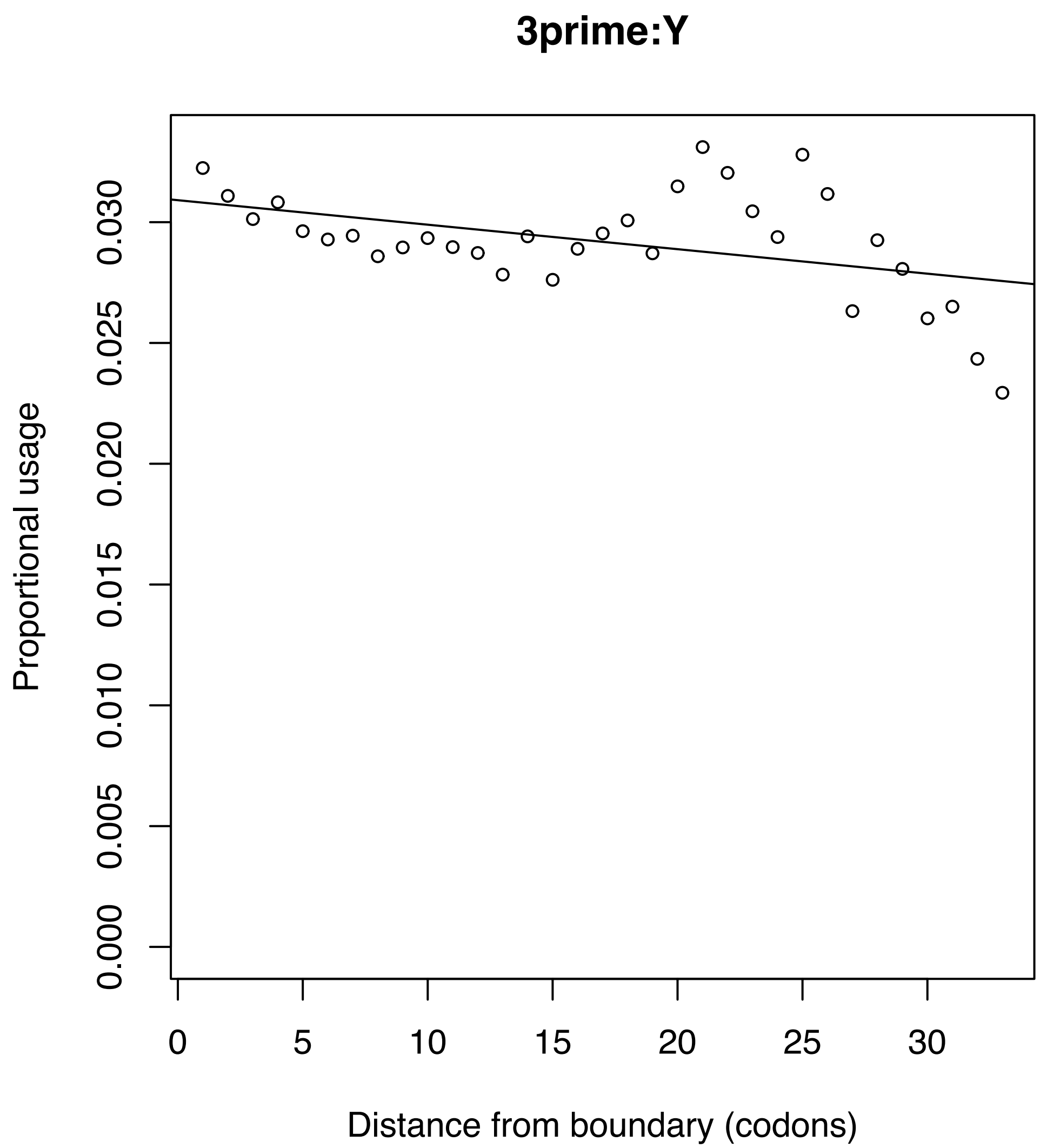
3prime:Y
Proportional usage
0.000
0.005
0.010
0.015
0.020
0.025
0.030
0
5
10
15
20
25
30
Distance from boundary (codons)

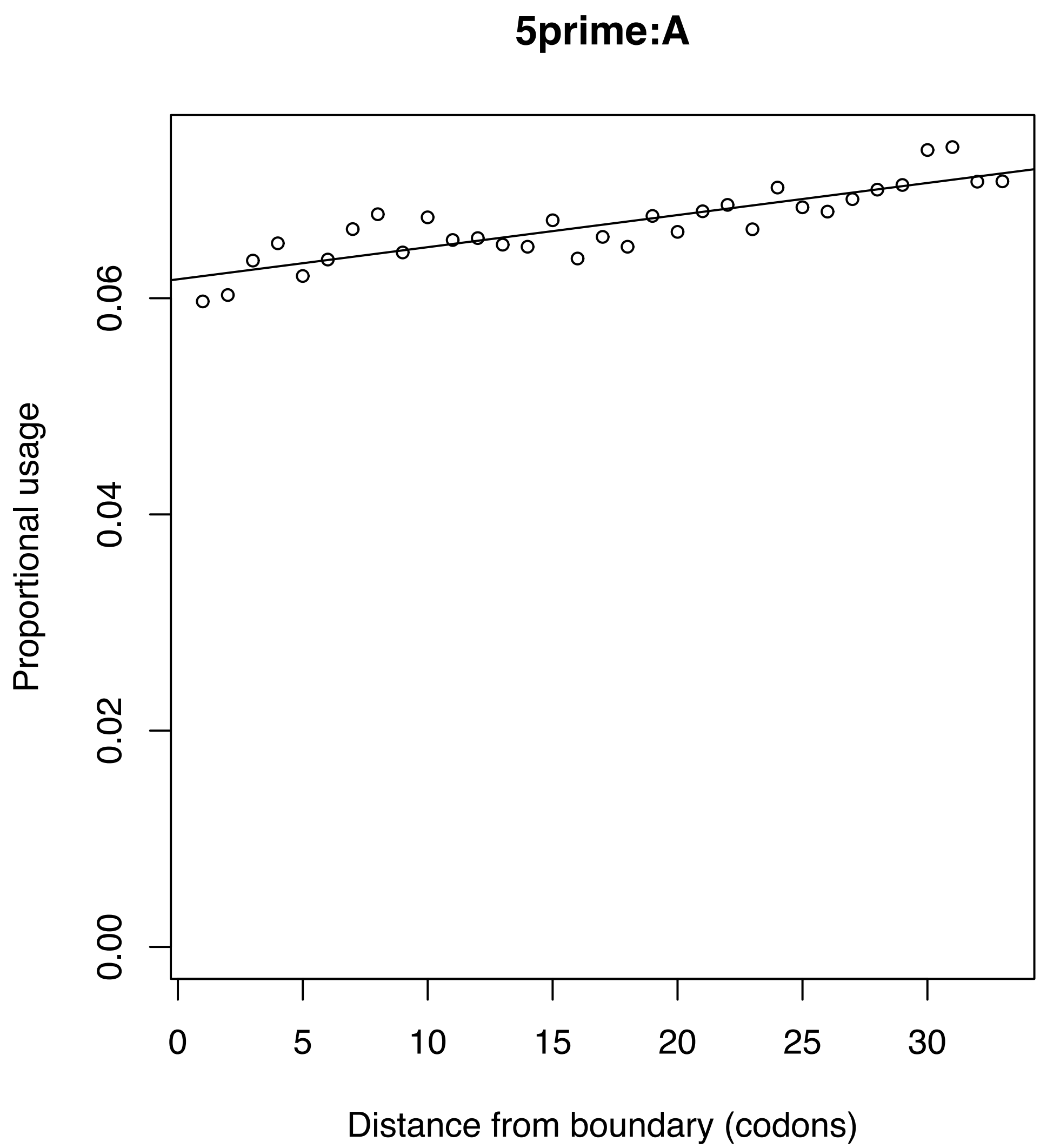
5prime:A
Proportional usage
0.00
0.02
0.04
0.06
0
5
10
15
20
25
30
Distance from boundary (codons)

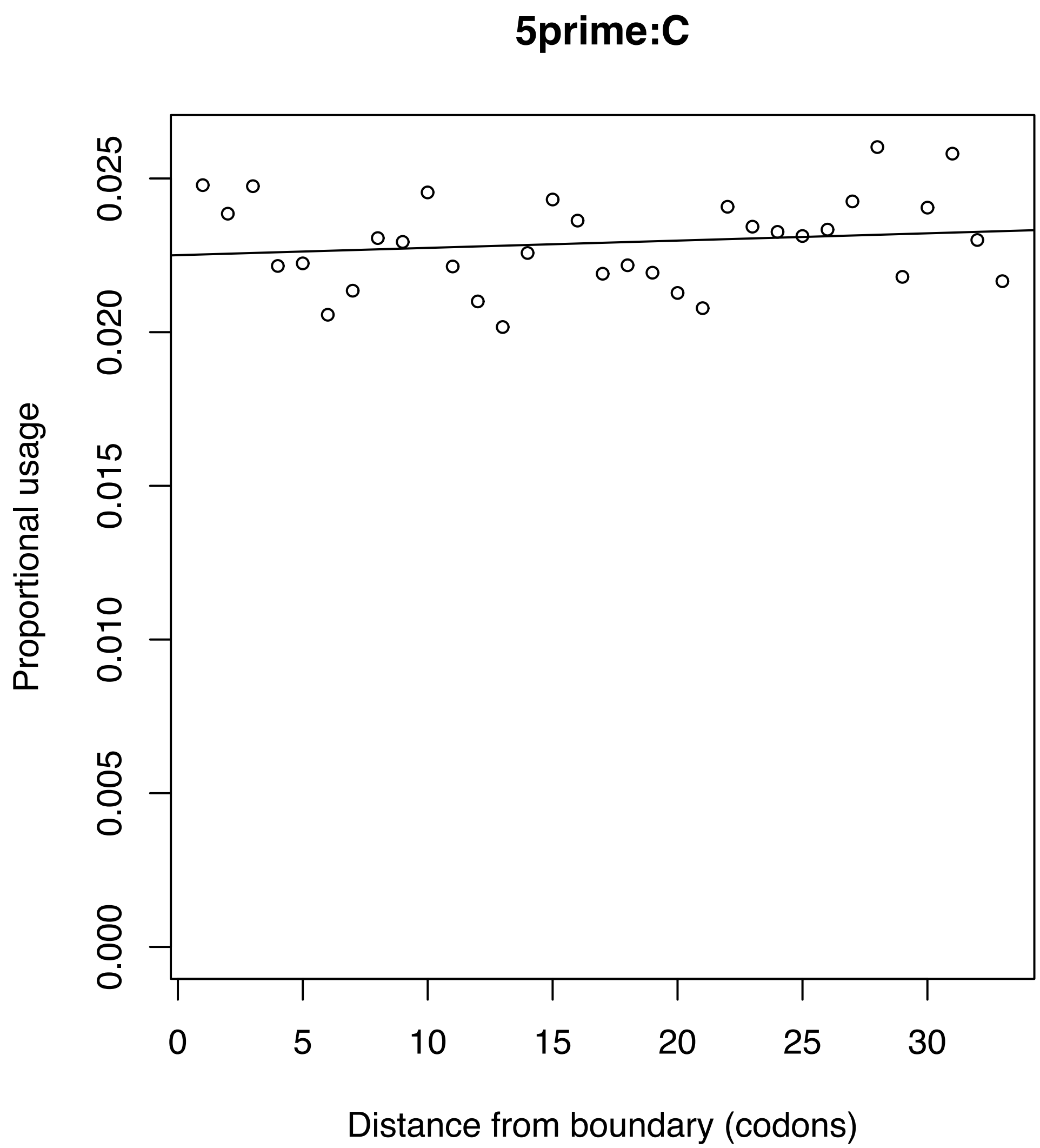
5prime:C
Proportional usage
0.000
0.005
0.010
0.015
0.020
0.025
0
5
10
15
20
25
30
Distance from boundary (codons)

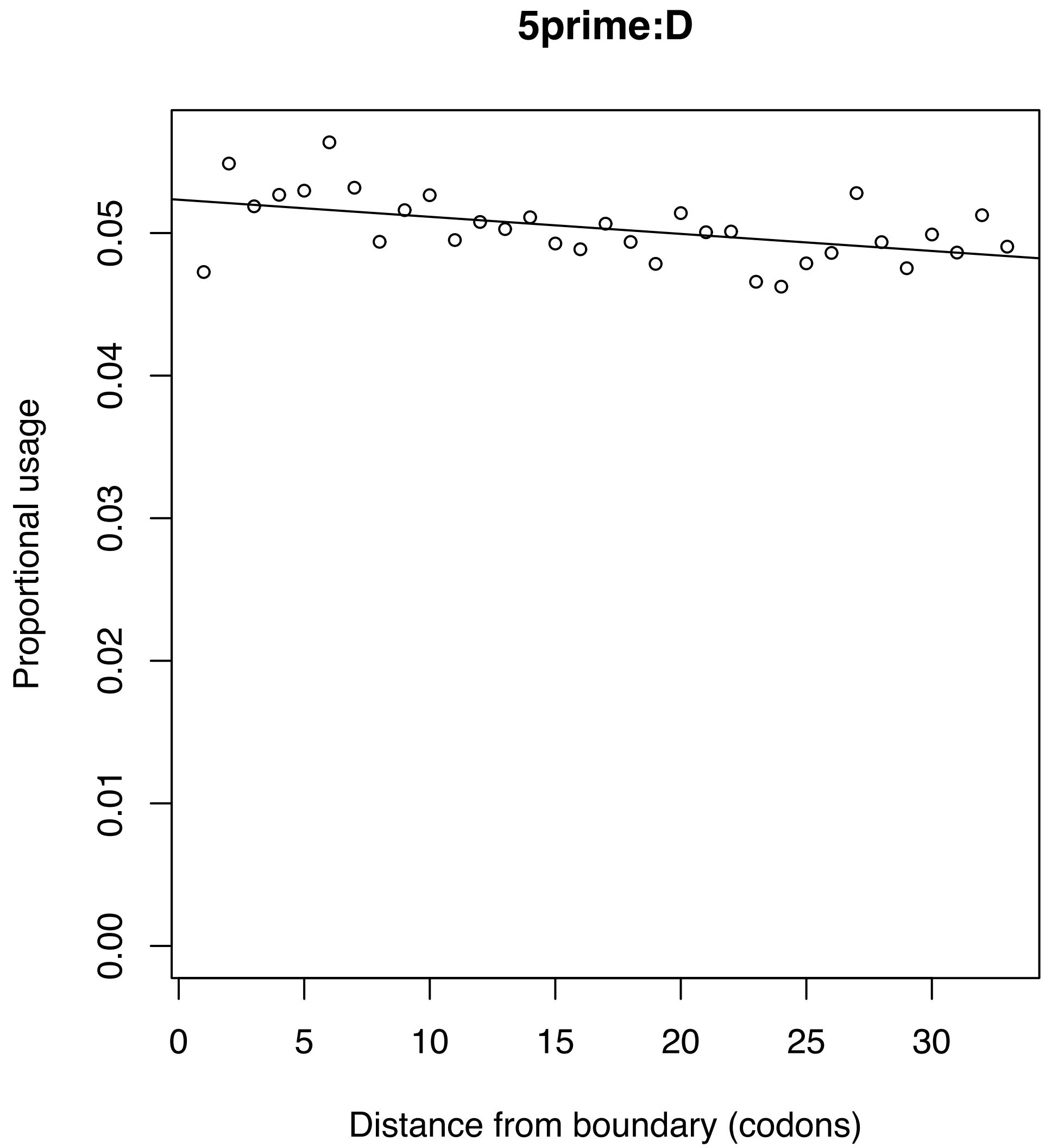
5prime:D
Proportional usage
0.00
0.01
0.02
0.03
0.04
0.05
0
5
10
15
20
25
30
Distance from boundary (codons)

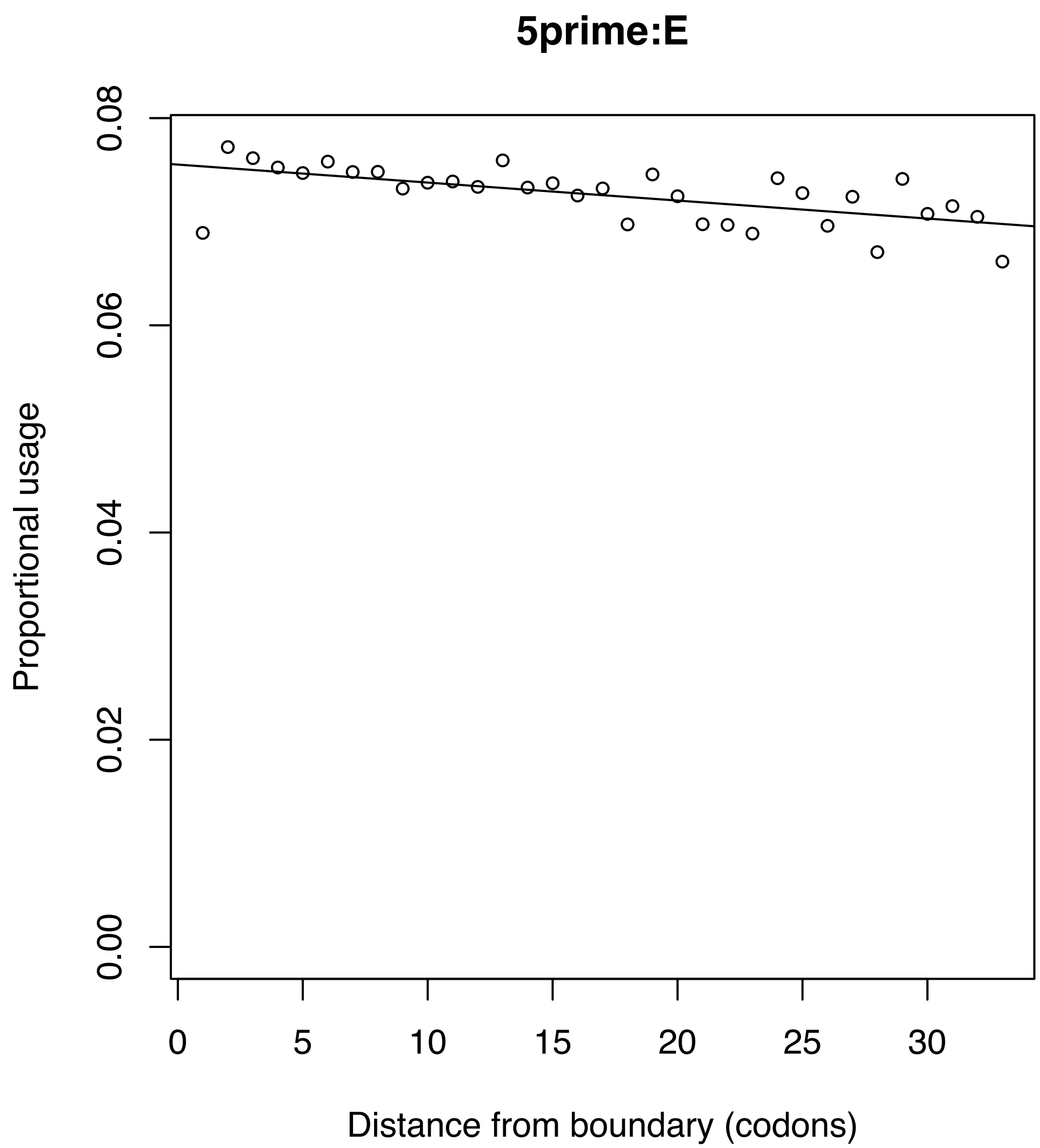
5prime:E
Proportional usage
0.00
0.02
0.04
0.06
0.08
0
5
10
15
20
25
30
Distance from boundary (codons)

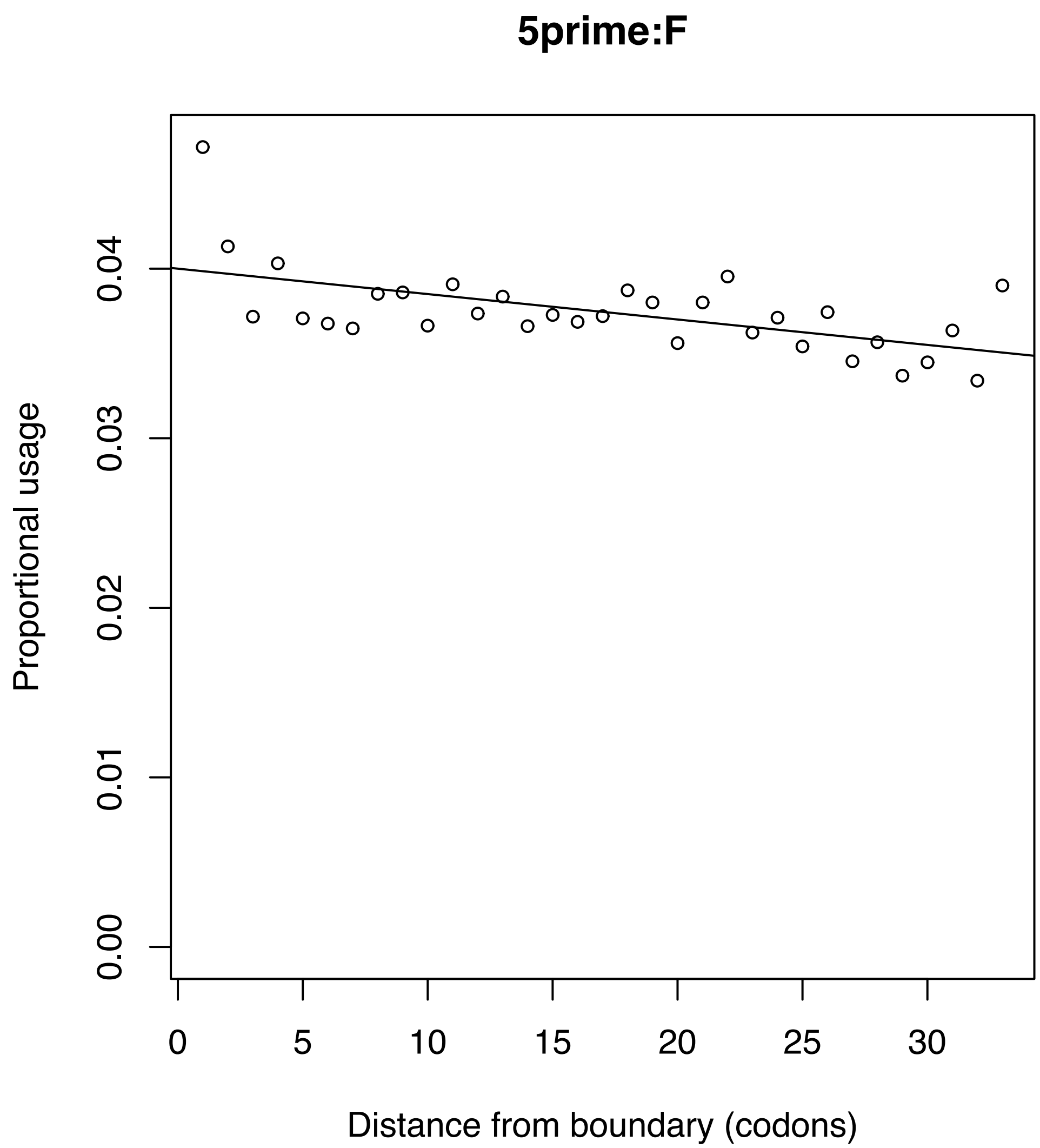
5prime:F
Proportional usage
0.00
0.01
0.02
0.03
0.04
0
5
10
15
20
25
30
Distance from boundary (codons)

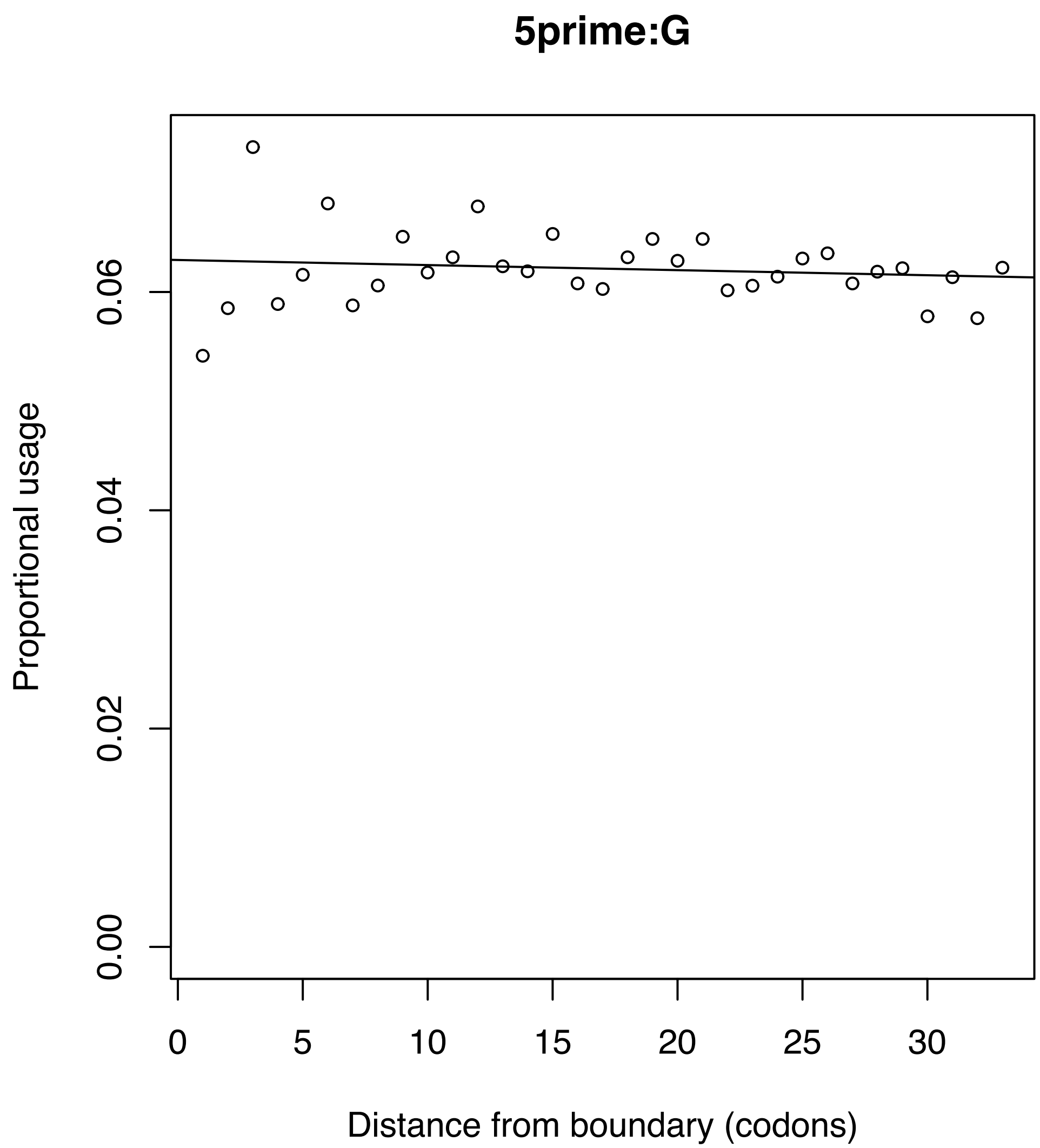
5prime:G
Proportional usage
0.00
0.02
0.04
0.06
0
5
10
15
20
25
30
Distance from boundary (codons)

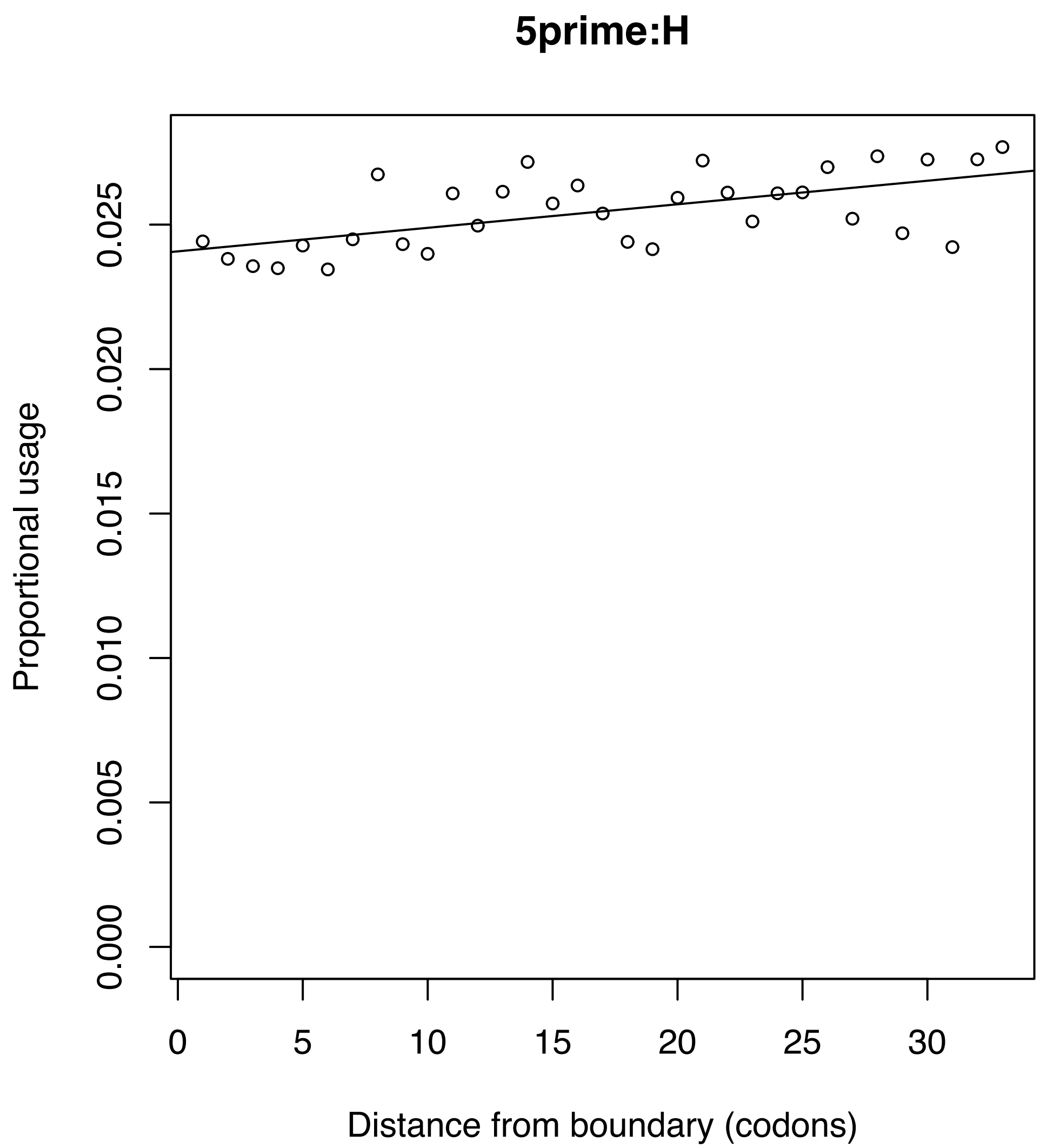
5prime:H
Proportional usage
0.000
0.005
0.010
0.015
0.020
0.025
0
5
10
15
20
25
30
Distance from boundary (codons)

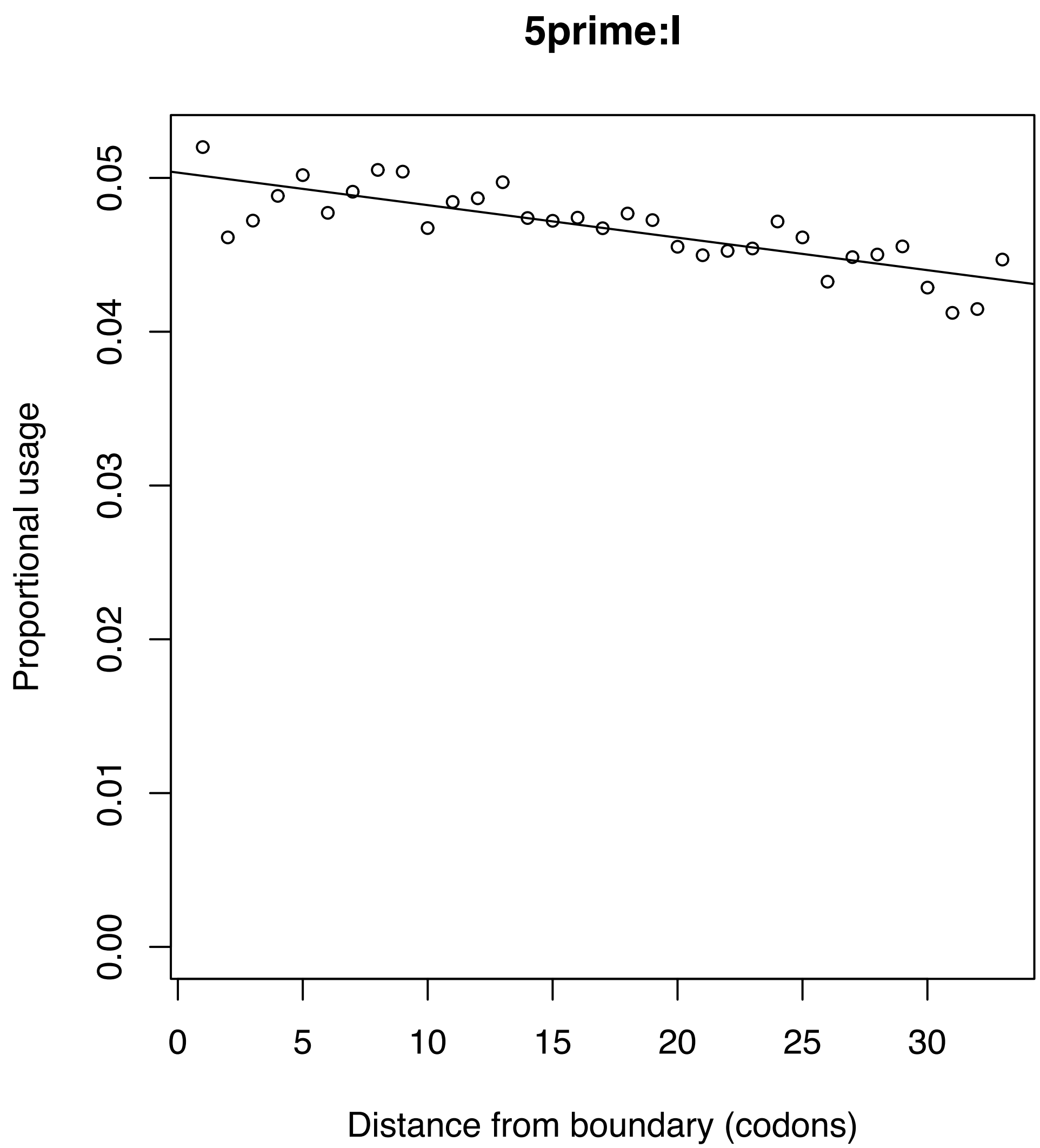
5prime:I
Proportional usage
0.00
0.01
0.02
0.03
0.04
0.05
0
5
10
15
20
25
30
Distance from boundary (codons)

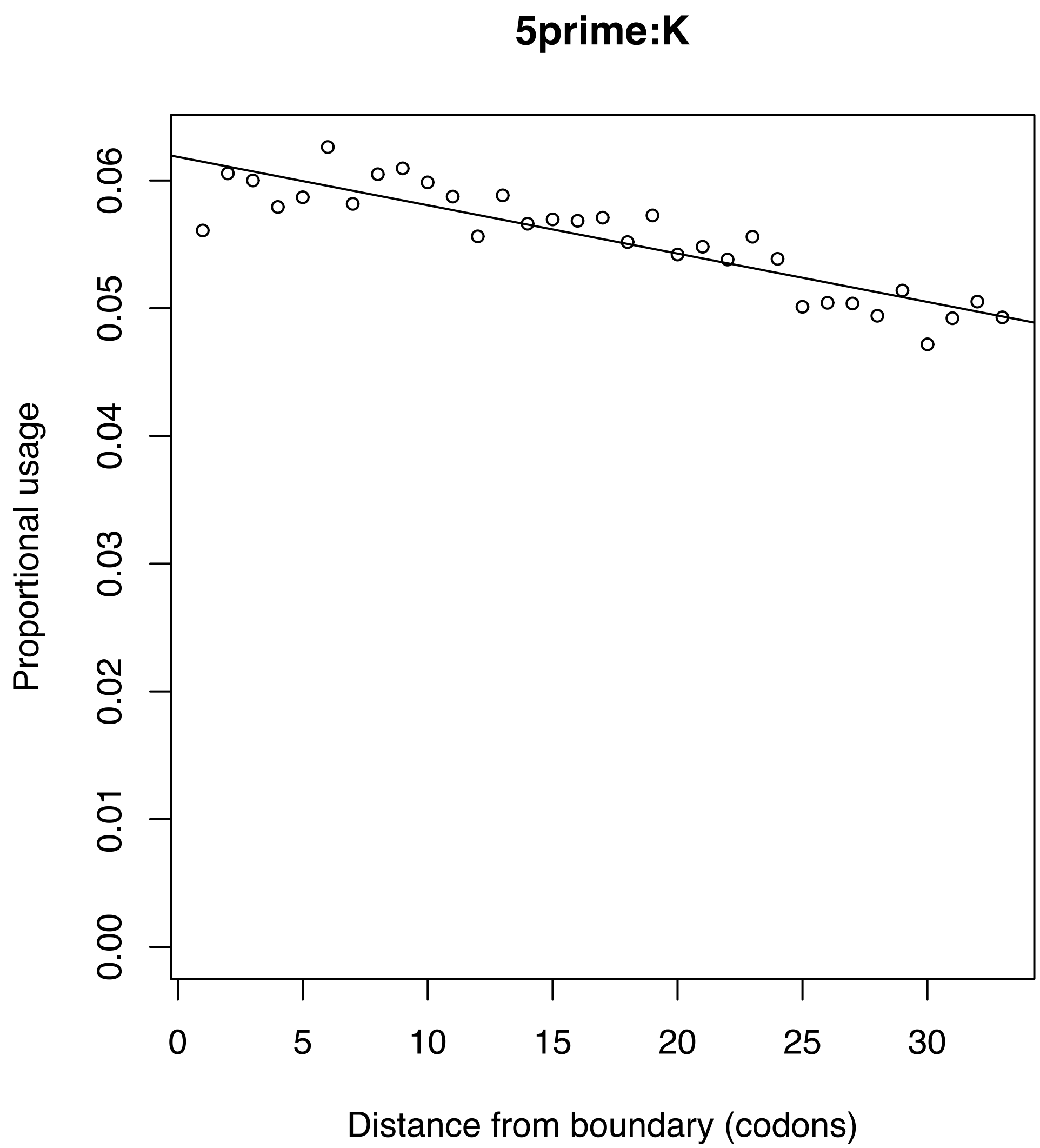
5prime:K
Proportional usage
0.00
0.01
0.02
0.03
0.04
0.05
0.06
0
5
10
15
20
25
30
Distance from boundary (codons)

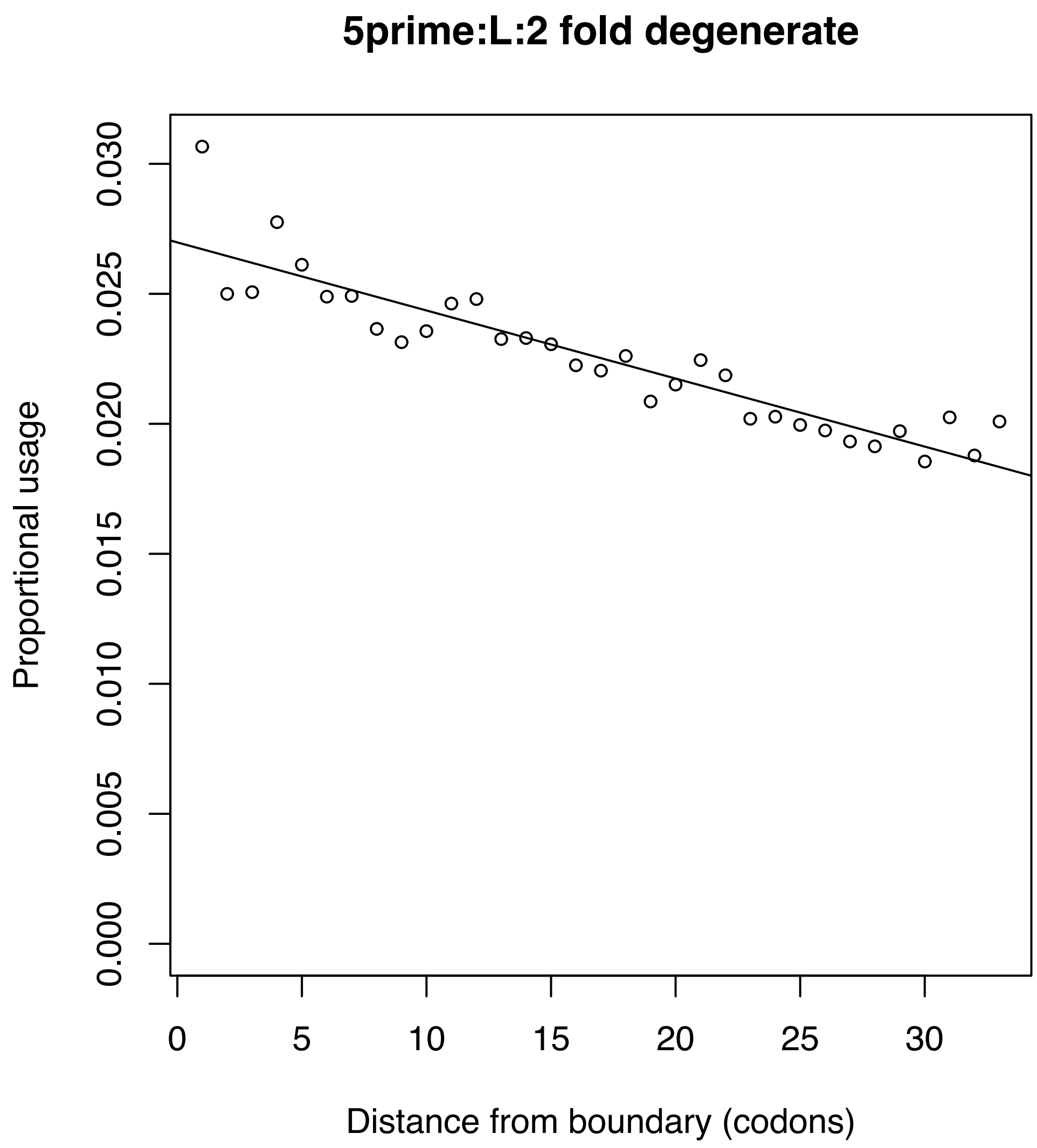
5prime:L:2 fold degenerate
Proportional usage
0.000
0.005
0.010
0.015
0.020
0.025
0.030
0
5
10
15
20
25
30
Distance from boundary (codons)

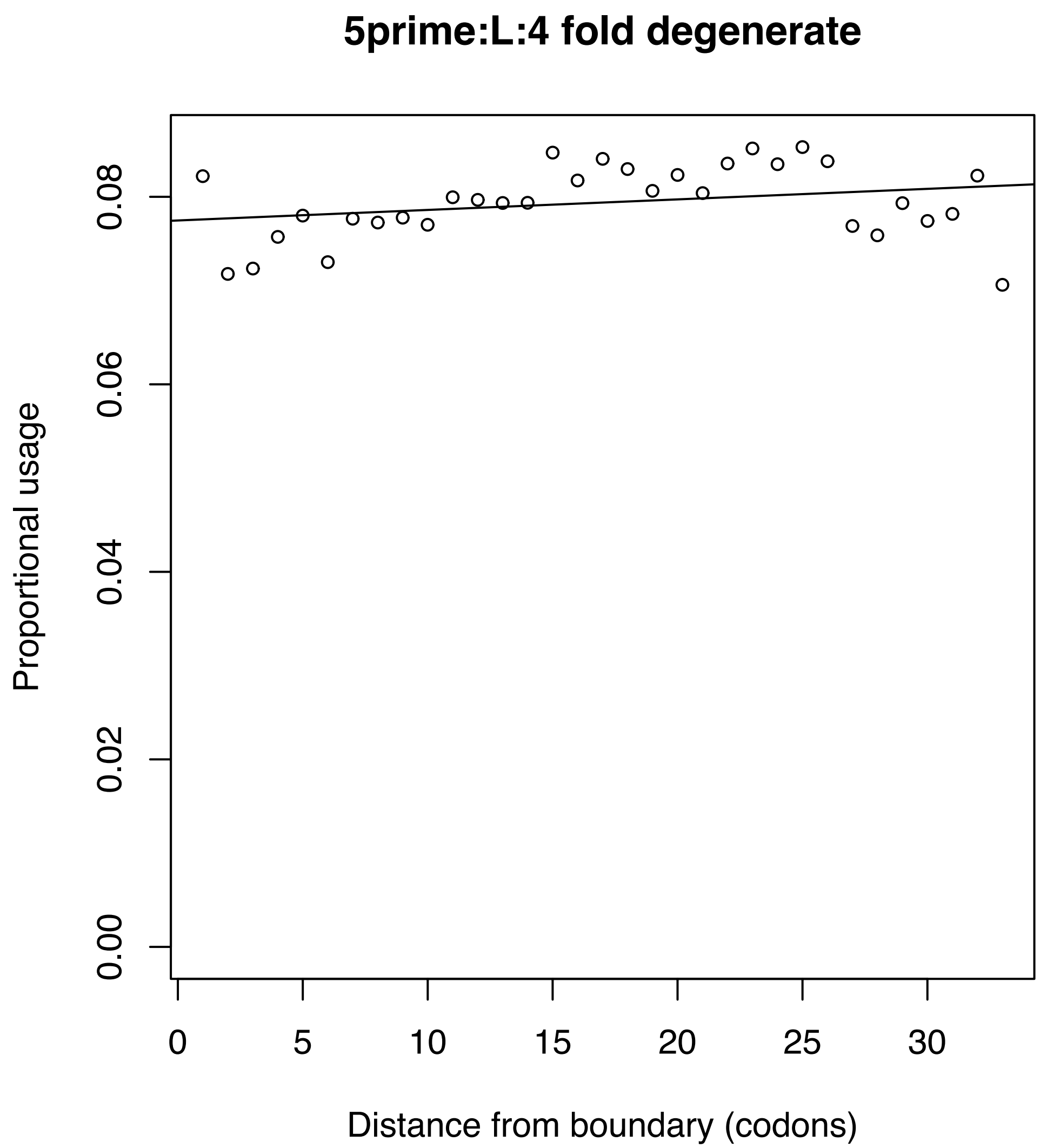

5prime:L:4 fold degenerate
Proportional usage
0.00
0.02
0.04
0.06
0.08
0
5
10
15
20
25
30
Distance from boundary (codons)

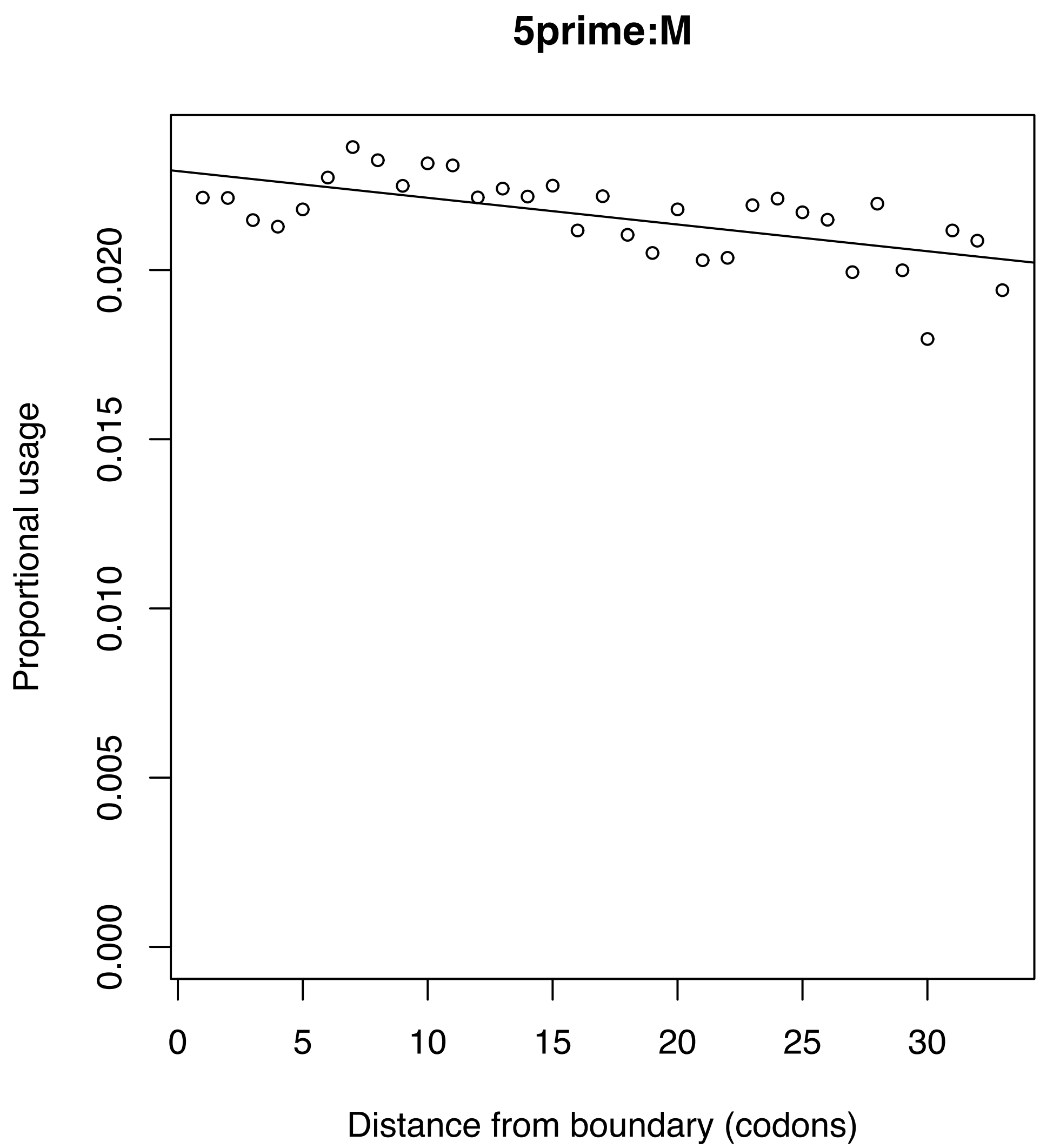
5prime:M
Proportional usage
0.000
0.005
0.010
0.015
0.020
0
5
10
15
20
25
30
Distance from boundary (codons)

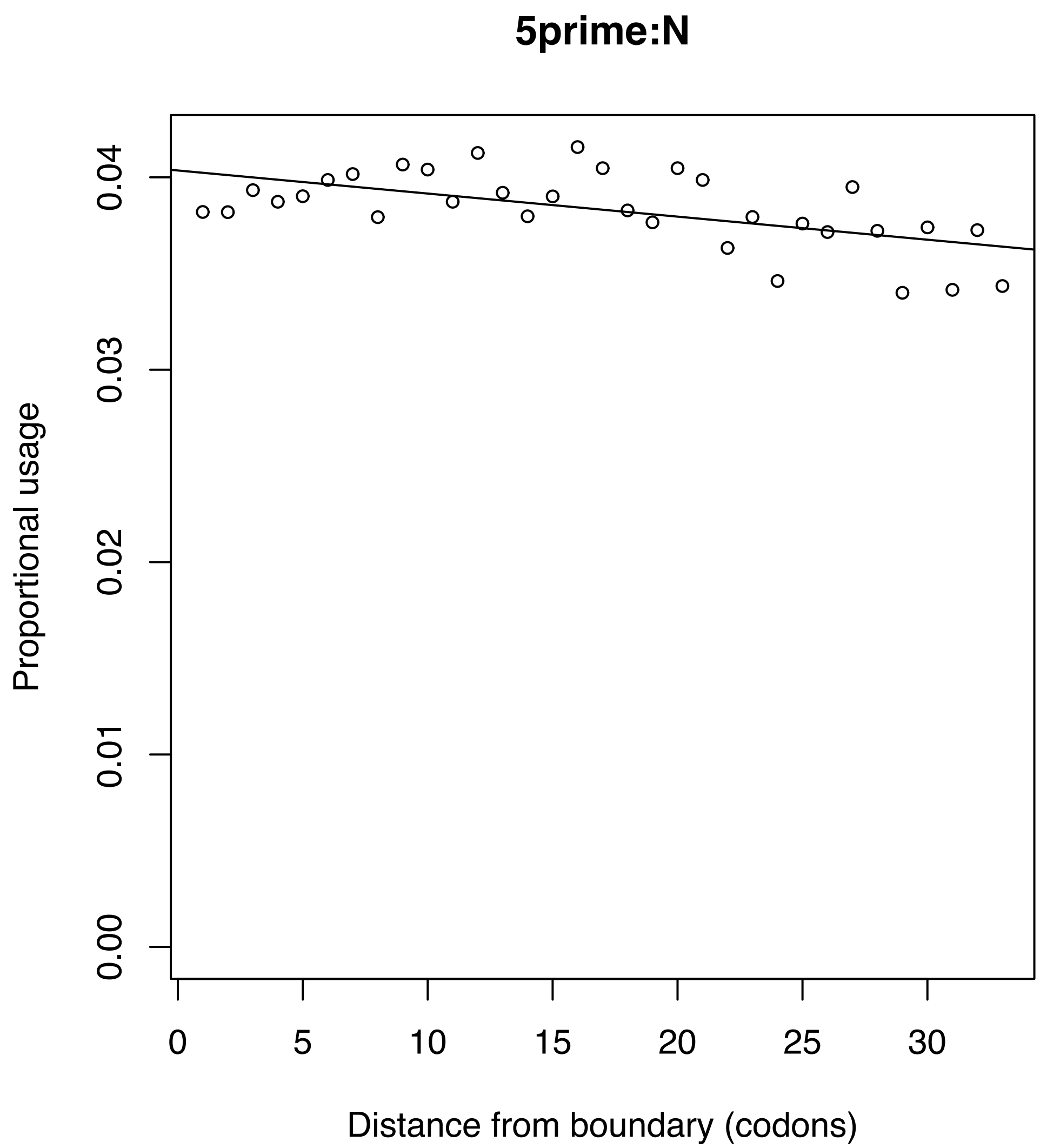
5prime:N
Proportional usage
0.00
0.01
0.02
0.03
0.04
0
5
10
15
20
25
30
Distance from boundary (codons)

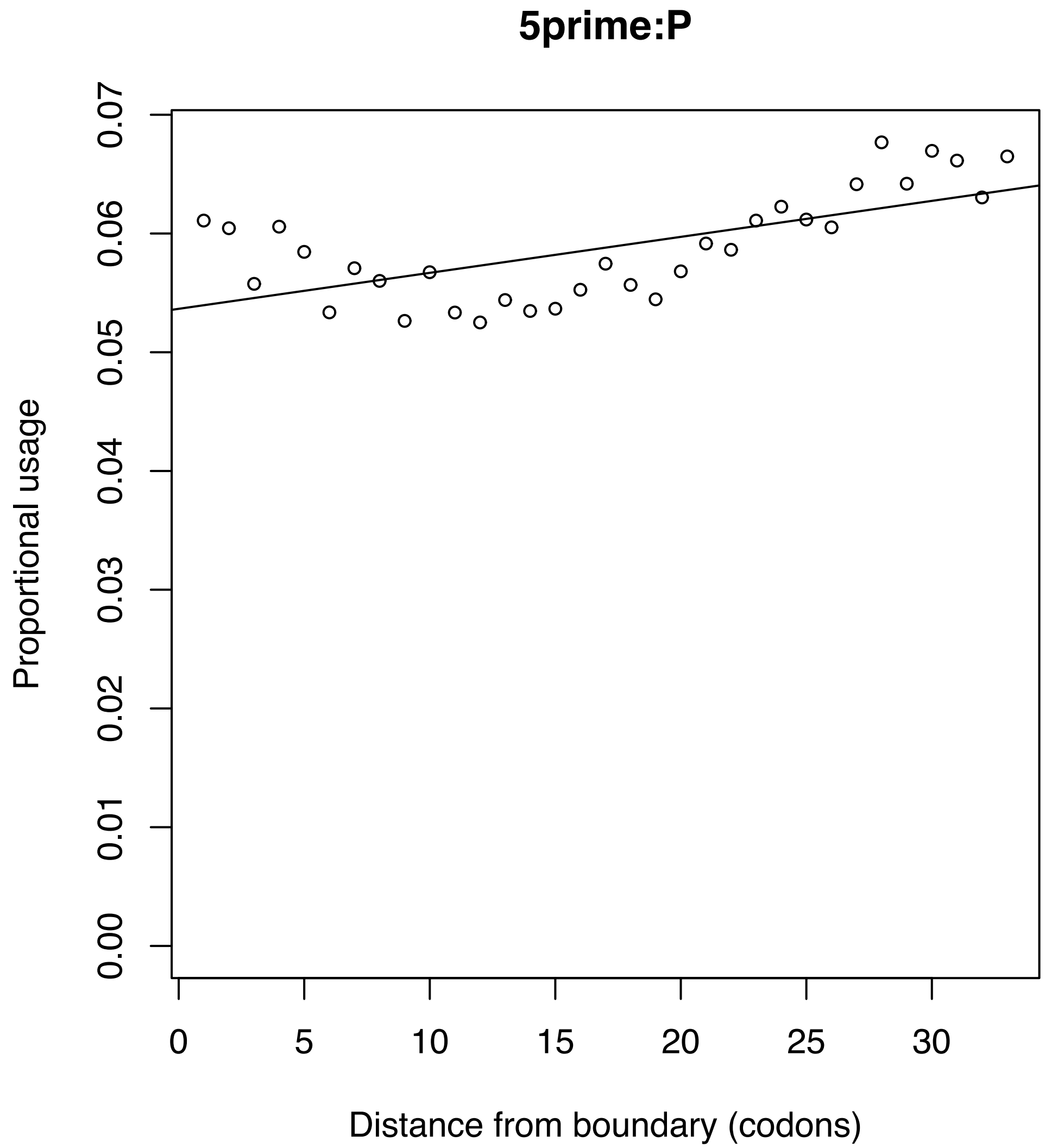

5prime:P
Proportional usage
0.00
0.01
0.02
0.03
0.04
0.05
0.06
0.07
0
5
10
15
20
25
30
Distance from boundary (codons)

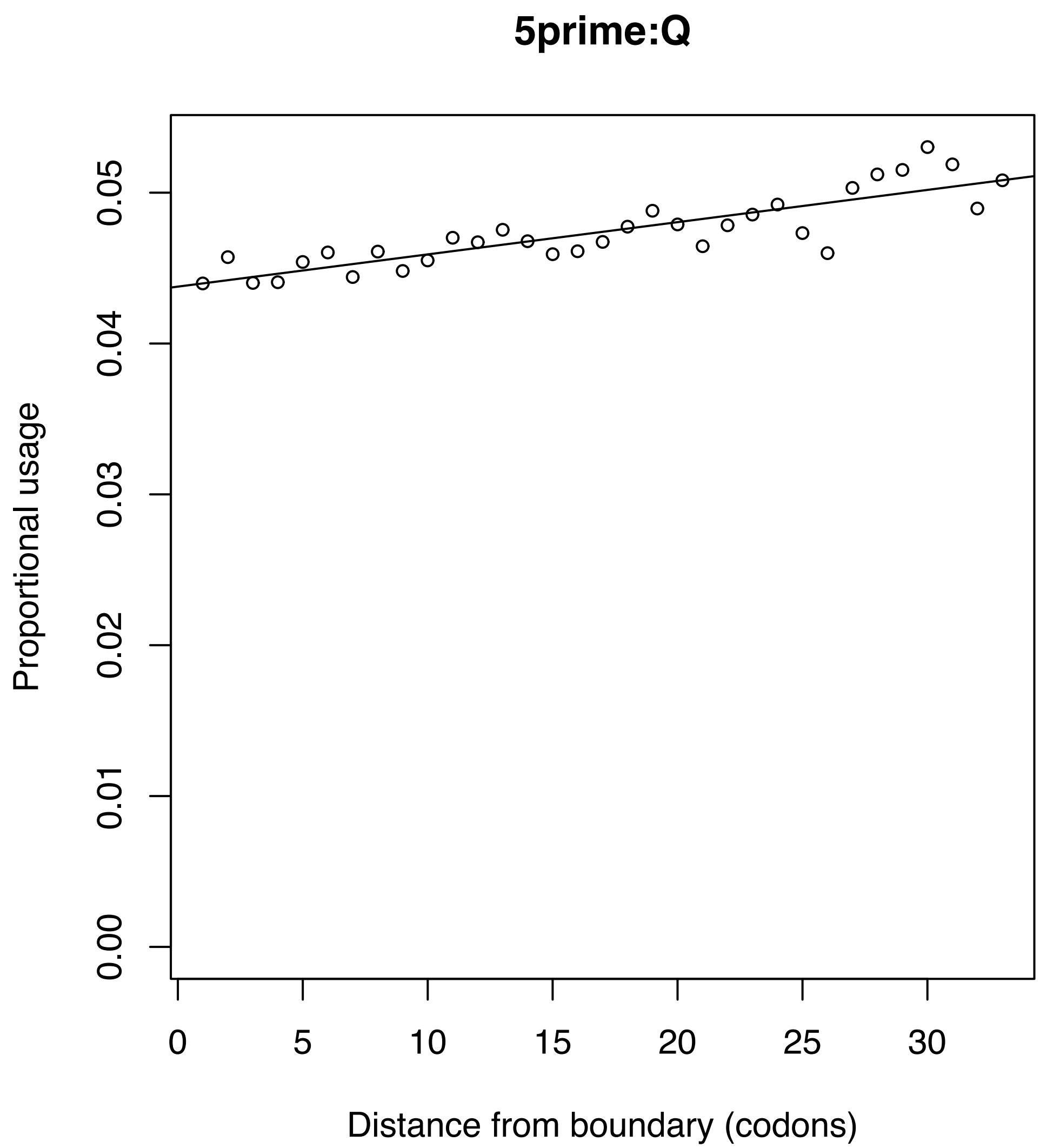

5prime:Q
Proportional usage
0.00
0.01
0.02
0.03
0.04
0.05
0
5
10
15
20
25
30
Distance from boundary (codons)

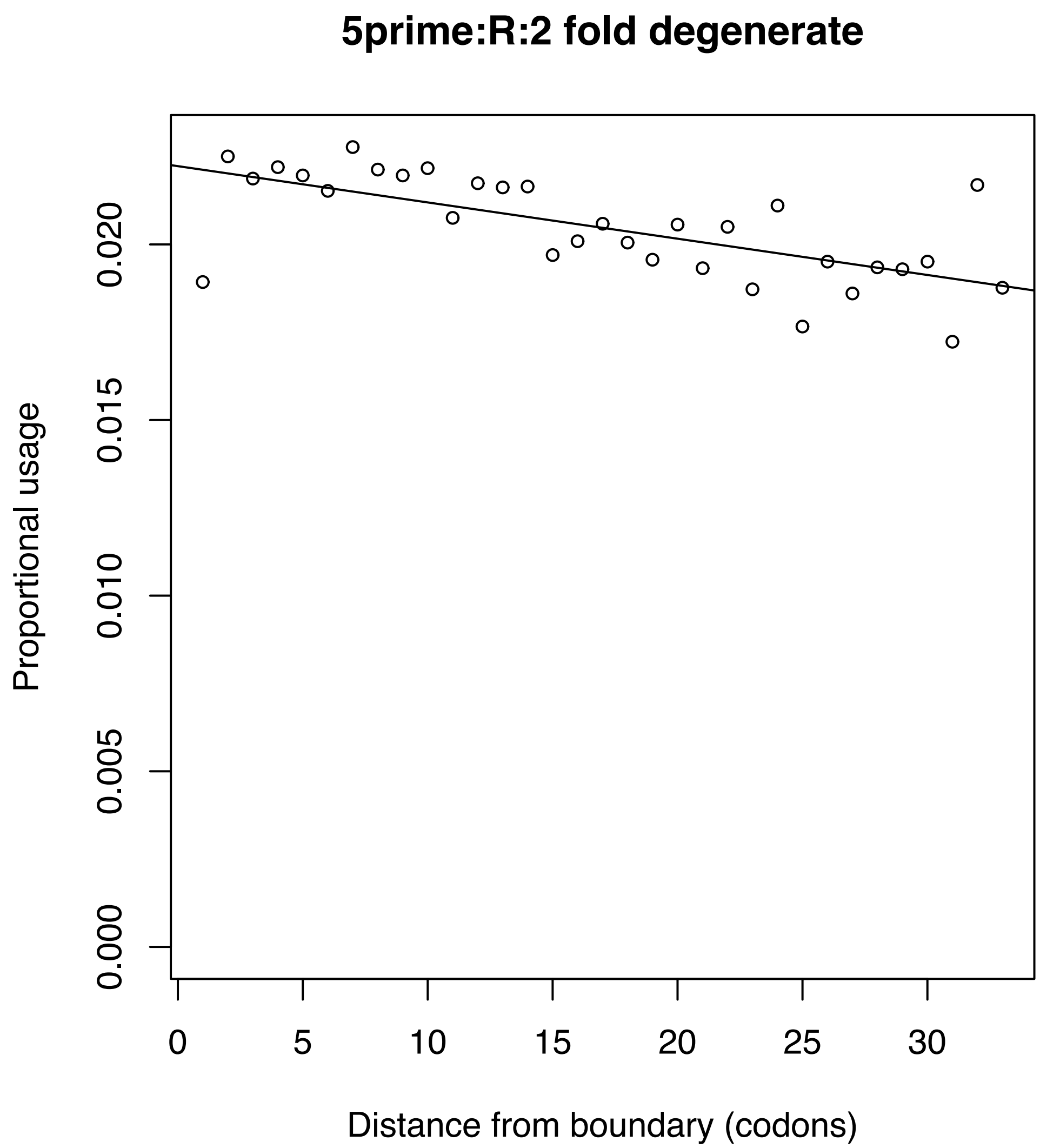
5prime:R:2 fold degenerate
Proportional usage
0.000
0.005
0.010
0.015
0.020
0
5
10
15
20
25
30
Distance from boundary (codons)

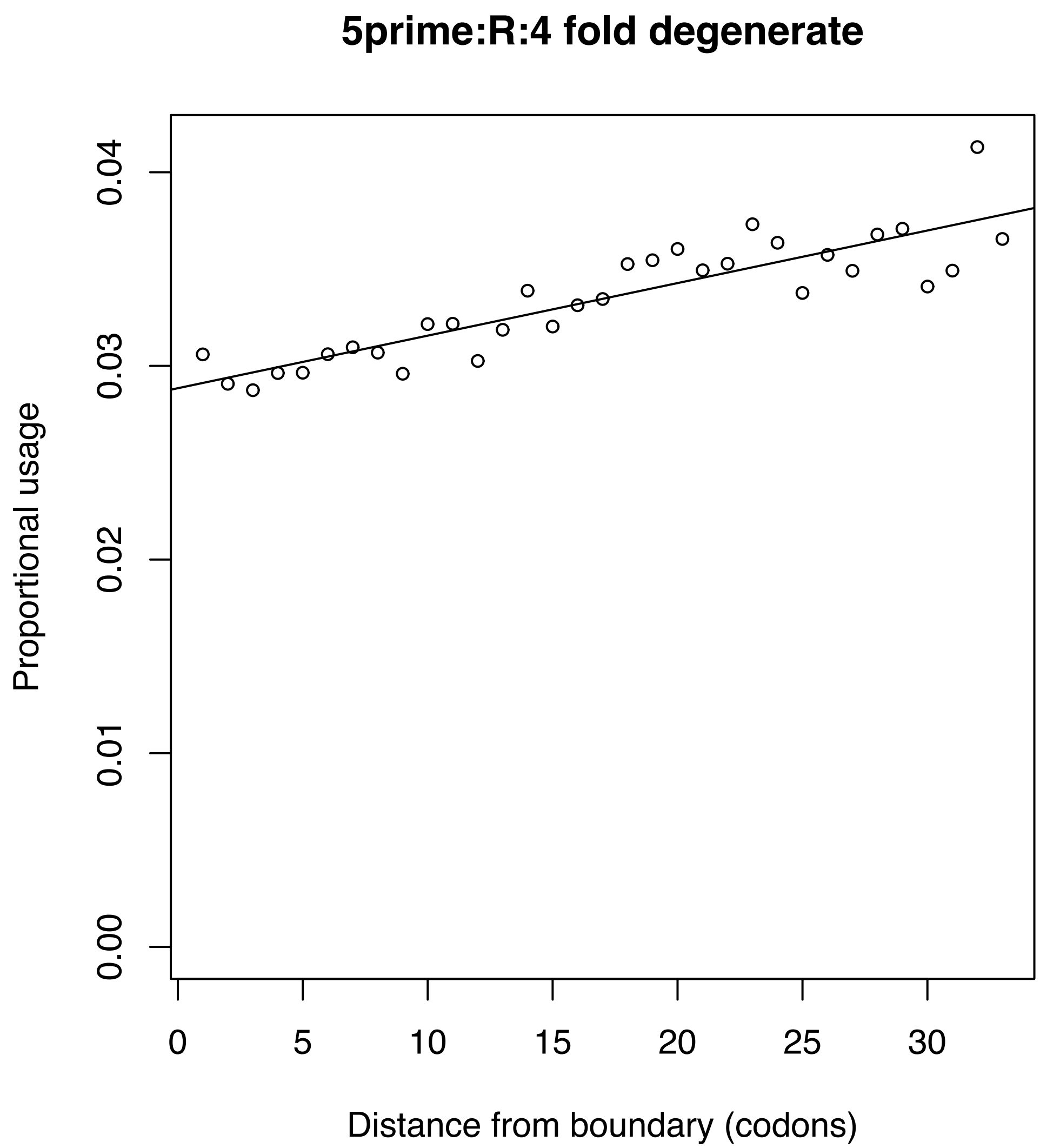

5prime:R:4 fold degenerate
Proportional usage
0.00
0.01
0.02
0.03
0.04
0
5
10
15
20
25
30
Distance from boundary (codons)

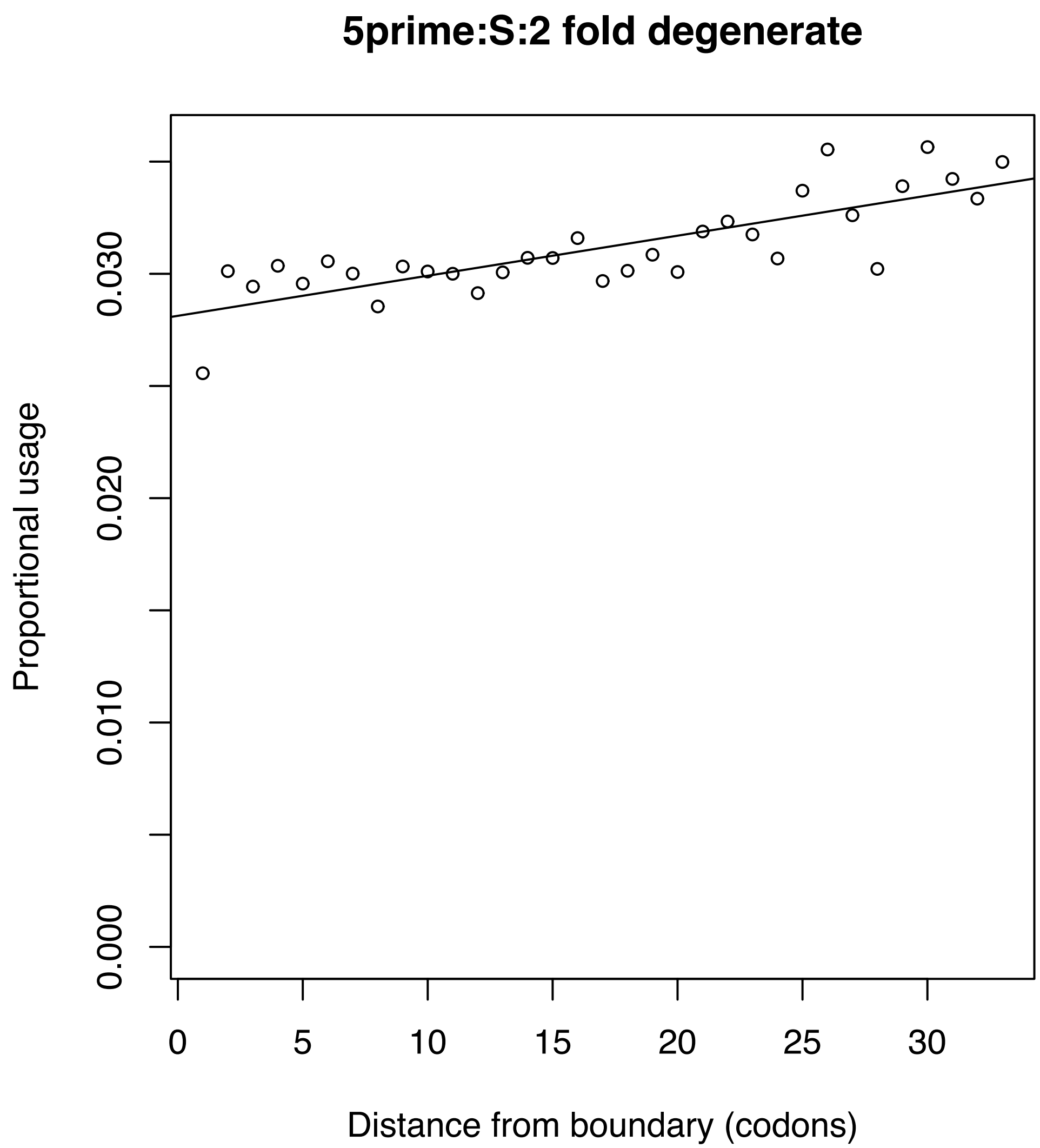
5prime:S:2 fold degenerate
Proportional usage
0.000
0.010
0.020
0.030
0
5
10
15
20
25
30
Distance from boundary (codons)

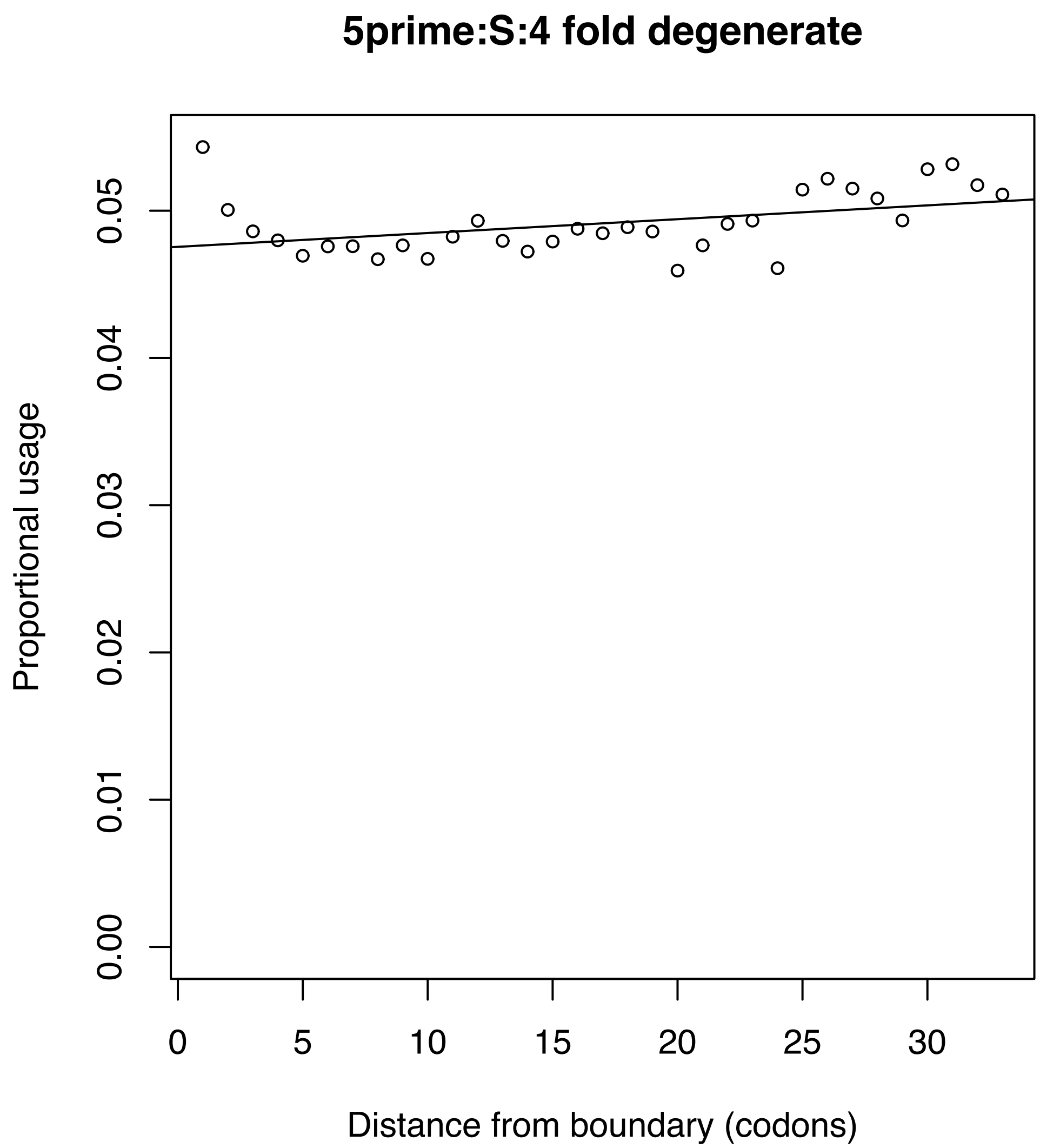
5prime:S:4 fold degenerate
Proportional usage
0.00
0.01
0.02
0.03
0.04
0.05
0
5
10
15
20
25
30
Distance from boundary (codons)

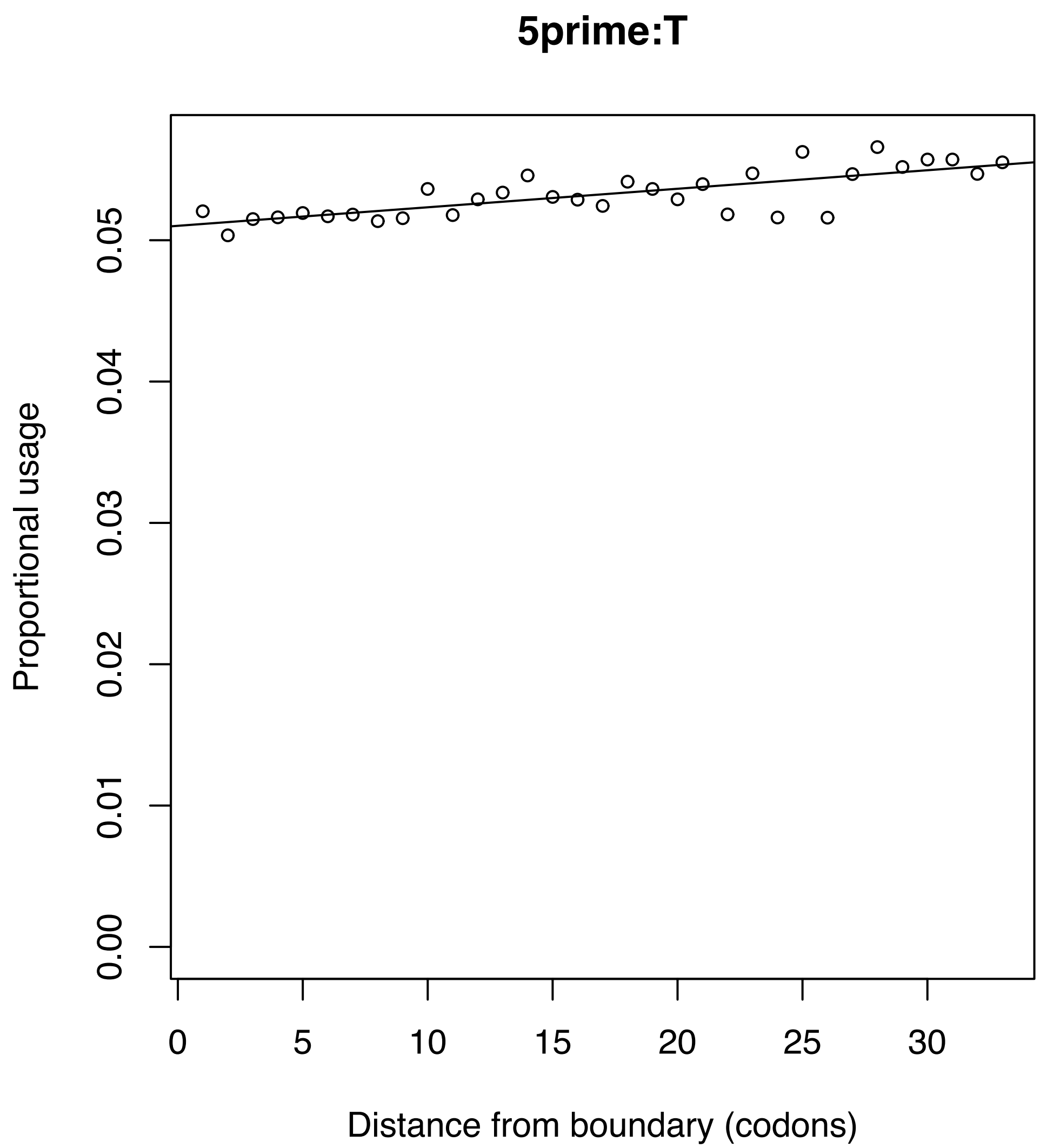

5prime:T
Proportional usage
0.00
0.01
0.02
0.03
0.04
0.05
0
5
10
15
20
25
30
Distance from boundary (codons)

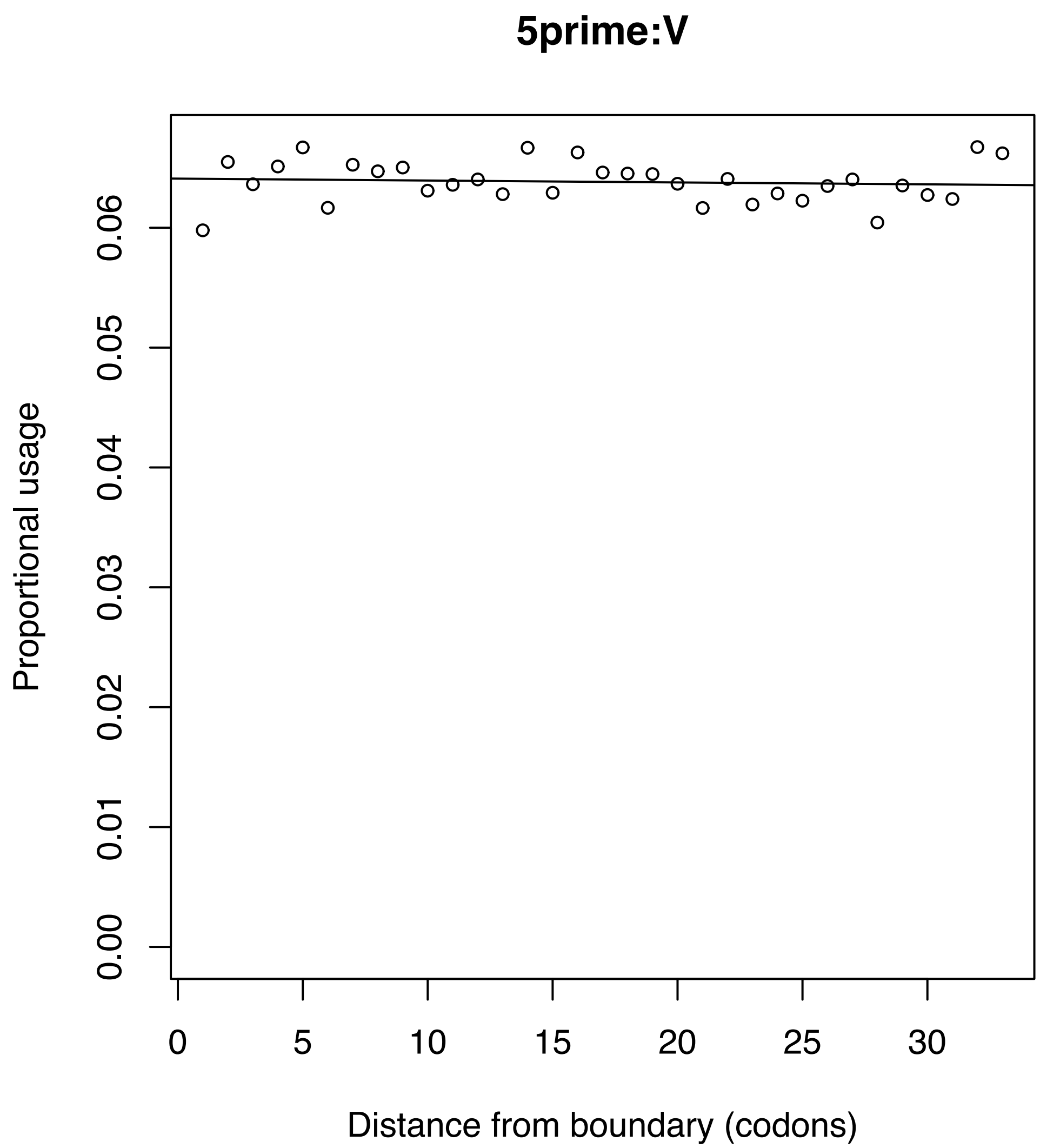
5prime:V
Proportional usage
0.00
0.01
0.02
0.03
0.04
0.05
0.06
0
5
10
15
20
25
30
Distance from boundary (codons)

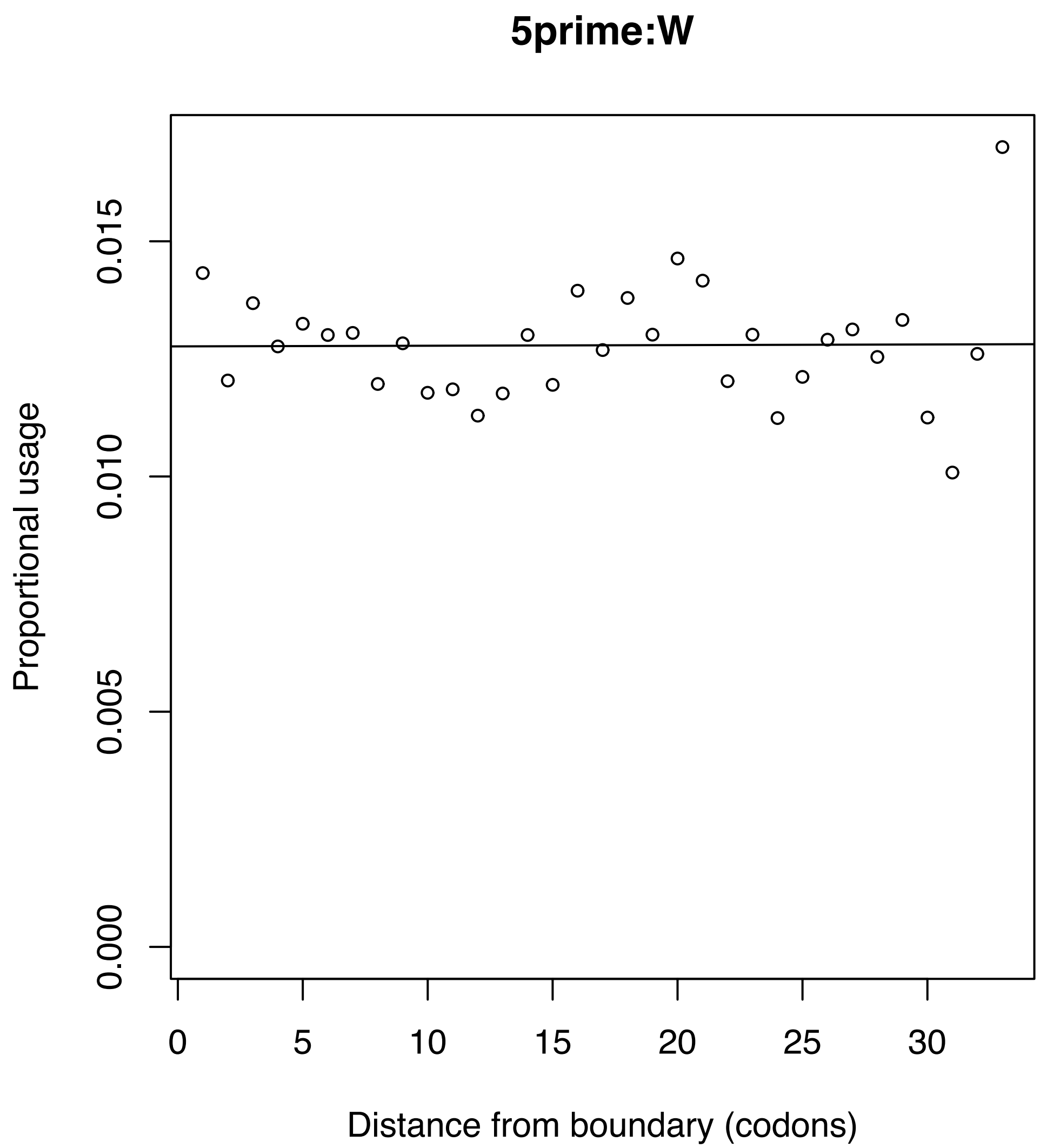
5prime:W
Proportional usage
0.000
0.005
0.010
0.015
0
5
10
15
20
25
30
Distance from boundary (codons)

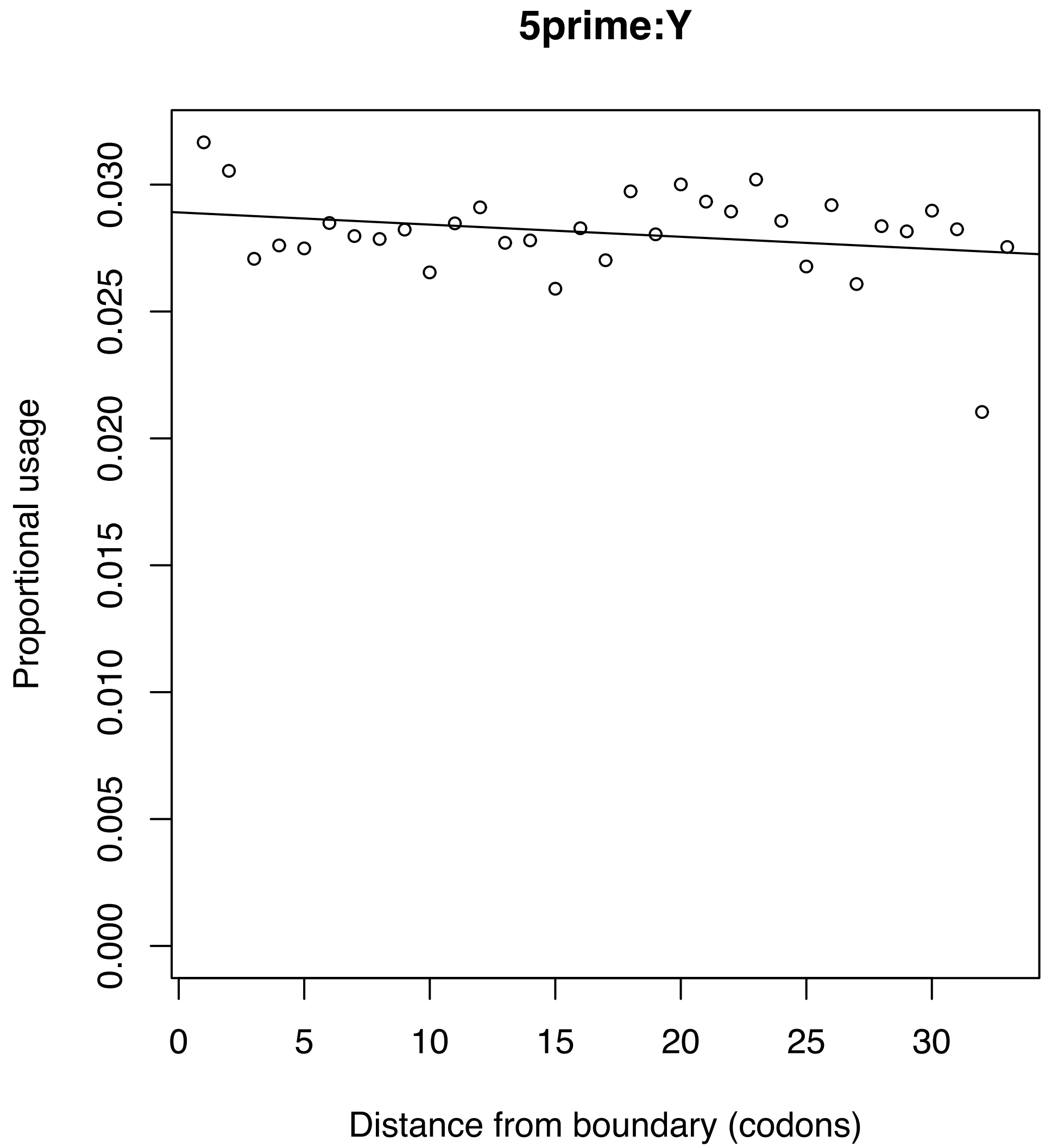
5prime:Y
Proportional usage
0.000
0.005
0.010
0.015
0.020
0.025
0.030
0
5
10
15
20
25
30
Distance from boundary (codons)
