# Supplementary material for: Splicing and the Evolution of Proteins in Mammals
Source: PLoS Biol. 2007 Feb 6;5(2):e14. doi: 10.1371/journal.pbio.0050014 (PMC1790955; doi:10.1371/journal.pbio.0050014)
Supplement: Supplementary file 2 [file pbio.0050014.sg002.doc]

**Figure S2.** Relationship between slope of regression line (between proportion of amino acid and distance from boundary) and HPI scores (a) 5’ and (b) 3’.

a.

(b)
