# Supplementary material for: Splicing and the Evolution of Proteins in Mammals
Source: PLoS Biol. 2007 Feb 6;5(2):e14. doi: 10.1371/journal.pbio.0050014 (PMC1790955; doi:10.1371/journal.pbio.0050014)
Supplement: Supplementary file 3 [file pbio.0050014.sg003.doc]

**Figure S3.** Rates of evolution in enhancer (circle) and non-enhancer (triangle) domains as a function of distance from the boundary for exons longer than 200 base pairs (i.e. in which all exons contribute equally to all data points). Here we merge 3’ and 5’ data.
