# Supplementary material for: Splicing and the Evolution of Proteins in Mammals
Source: PLoS Biol. 2007 Feb 6;5(2):e14. doi: 10.1371/journal.pbio.0050014 (PMC1790955; doi:10.1371/journal.pbio.0050014)
Supplement: Supplementary file 4 [file pbio.0050014.sg004.doc]

**Figure S4.** Plot of standardised residuals for the regression of AC content versus rho. Grey lines indicate top and bottom 95% confidence intervals.
