# Supplementary material for: Splicing and the Evolution of Proteins in Mammals
Source: PLoS Biol. 2007 Feb 6;5(2):e14. doi: 10.1371/journal.pbio.0050014 (PMC1790955; doi:10.1371/journal.pbio.0050014)
Supplement: Supplementary file 6 [file pbio.0050014.sg006.doc]

**Figure S6.** Glycine usage as a function of frame of exon and exonic end. The first number in the title is the exonic end (5 or 3), the second the phase (0,1 or 2).
