# Supplementary material for: Splicing and the Evolution of Proteins in Mammals
Source: PLoS Biol. 2007 Feb 6;5(2):e14. doi: 10.1371/journal.pbio.0050014 (PMC1790955; doi:10.1371/journal.pbio.0050014)
Supplement: Supplementary file 7 [file pbio.0050014.sg007.doc]

**Figure S7.** Periodicity analysis of glycine and proline.

For (a) Glycine and (b) Proline the distribution of homologous residues in the flanking sequence was determined. The first such residue in the sequence was taken as the reference point 0, once frequency data for the same amino acid was obtained, for the 150 flanking residues, the reference point moved along to the next homologous residue. The frequency of the residue in the flanking sequence was then determined by the absolute occurrence of the residue at this distance, divided by the number of informative sites. Glycine exhibits an unusual pattern where, following the use of a glycine, there is a preference for glycine to be used every third amino acid (top series of points in fig a). This is not an artefact of contamination by collagen transcripts (GPX_n_), as the distribution of proline indicates no such trend. This pattern in glycine usage is still strong over 500 residues away from the reference point.

a)

b)
