# Supplementary material for: Splicing and the Evolution of Proteins in Mammals
Source: PLoS Biol. 2007 Feb 6;5(2):e14. doi: 10.1371/journal.pbio.0050014 (PMC1790955; doi:10.1371/journal.pbio.0050014)
Supplement: Supplementary file 8 [file pbio.0050014.st001.doc]

Supplementary Table 1. Correlations and partial correlations between rate of protein evolution (*K*_A_ or *K*_A_ / *K*_S_), proportion of sequence within 50, 70 or 100 base pairs of an intron-exon junction and measures of expression of the relevant gene in mouse.

| X | Y | Z | *R*_XY_ | *R*_XY\|Z_ | *P*_XY\|Z_ | *R*_XZ_ | *R*_XZ\|Y_ | *P*_XY\|Z_ | *R*_YZ_ |
| --- | --- | --- | --- | --- | --- | --- | --- | --- | --- |
| Ka | Proportion 50 | Breadth | -0.1984 | -0.1594 | 1.00E-04 | -0.2159 | -0.1809 | 1.00E-04 | 0.2151 |
| Ka | Proportion 50 | Median | -0.2055 | -0.1964 | 1.00E-04 | -0.1355 | -0.1209 | 1.00E-04 | 0.0856 |
| Ka | Proportion 50 | Peak rate | -0.2055 | -0.2029 | 1.00E-04 | -0.0370 | -0.0172 | 0.20358 | 0.0988 |
| Ka/Ks | Proportion 50 | Breadth | -0.2064 | -0.1689 | 1.00E-04 | -0.2100 | -0.1733 | 1.00E-04 | 0.2151 |
| Ka/Ks | Proportion 50 | Median | -0.2175 | -0.2085 | 1.00E-04 | -0.1375 | -0.1222 | 1.00E-04 | 0.0856 |
| Ka/Ks | Proportion 50 | Peak rate | -0.2175 | -0.2145 | 1.00E-04 | -0.0467 | -0.0260 | 0.1077 | 0.0988 |
| Ka | Proportion 70 | Breadth | -0.2007 | -0.1620 | 1.00E-04 | -0.2159 | -0.1807 | 1.00E-04 | 0.2138 |
| Ka | Proportion 70 | Median rate | -0.2066 | -0.1964 | 1.00E-04 | -0.1355 | -0.1190 | 1.00E-04 | 0.0951 |
| Ka | Proportion 70 | Peak rate | -0.2066 | -0.2038 | 1.00E-04 | -0.0370 | -0.0135 | 0.2650 | 0.1160 |
| Ka/Ks | Proportion 70 | Breadth | -0.2115 | -0.1745 | 1.00E-04 | -0.2100 | -0.1725 | 1.00E-04 | 0.2138 |
| Ka/Ks | Proportion 70 | Median | -0.2219 | -0.2118 | 1.00E-04 | -0.1375 | -0.1199 | 1.00E-04 | 0.0951 |
| Ka/Ks | Proportion 70 | Peak rate | -0.2219 | -0.2182 | 1.00E-04 | -0.0467 | -0.0217 | 0.1500 | 0.1160 |
| Ka | Proportion 100 | Breadth | -0.2030 | -0.1617 | 1.00E-04 | -0.2159 | -0.1779 | 1.00E-04 | 0.2278 |
| Ka | Proportion 100 | Median | -0.2068 | -0.1933 | 1.00E-04 | -0.1355 | -0.1134 | 1.00E-04 | 0.1229 |
| Ka | Proportion 100 | Peak rate | -0.2068 | -0.2037 | 1.00E-04 | -0.0370 | -0.0063 | 0.37916 | 0.1495 |
| Ka/Ks | Proportion 100 | Breadth | -0.2138 | -0.1744 | 1.00E-04 | -0.2100 | -0.1695 | 1.00E-04 | 0.2278 |
| Ka/Ks | Proportion 100 | Median | -0.2227 | -0.2094 | 1.00E-04 | -0.1375 | -0.1138 | 1.00E-04 | 0.1229 |
| Ka/Ks | Proportion 100 | Peak rate | -0.2227 | -0.2184 | 1.00E-04 | -0.0467 | -0.0139 | 0.26057 | 0.1495 |
